# Supplementary material for: The Brazilian COVID-19 vaccination campaign: a modelling analysis of sociodemographic factors on uptake
Source: BMJ Open. 2024 Jan 16;14(1):e076354. doi: 10.1136/bmjopen-2023-076354 (PMC10806735; doi:10.1136/bmjopen-2023-076354)
Supplement: Supplementary data [file bmjopen-2023-076354supp001.pdf]

Mortality

Logistic regression analysis of mortality

2023-10-27

Table of contents

Packages . . . 1
Data files . . . 2
  Output files . . . 2
  Input files . . . 2
  Check data files exist . . . 2
Satellite cities and excluded municipality codes . . . 3
Socio-economic (census) data . . . 4
Vaccination campaign data . . . 5
  Old preprocessing steps . . . 5
Vaccination coverage data . . . 6
  Old preprocessing steps . . . 6
SIVEP-Gripe dataset . . . 7
  Preprocessing function . . . 7
  Preparing the data . . . 8
Combined dataframe . . . 9
GLM . . . 11
  LaTeX table summarising the model fit . . . 13
Session information . . . 14

Packages

```
library(dplyr)
library(readr)
library(lubridate)
library(car)
```


Li SL, *et al. BMJ Open* 2024; 14:e076354. doi: 10.1136/bmjopen-2023-076354

```
library(xtable)
```

- The `readr` package provides a function, `read_delim` that is faster than the built-in `read.csv` function and provides a progress bar.
- The `car` package provides the `vif` function which we use to check for a concerning about of covariation between the covariates of our model.
- The `xtable` package is needed to make a LaTeX table summarising the final model fit.

Data files

Output files

```
output_files <- list(
  final_dataset = "out/final-mortality-dataset.csv",
  final_model_table = "out/final-mortality-model.tex"
)
```

Input files

Data files live in `data/`.

```
input_data <- list(
  sivep_2020 = "data/sivep_2020.csv",
  sivep_2021 = "data/sivep_20092021.csv",
  socio_econ_2010 = "data/census2010_muni_covariates.csv",
  vaccination_dates = "out/vaccination-halfway-dates.rds",
  vaccination_coverage = "out/vaccination-age-muni-date-counts.rds"
  )
```

Check data files exist

```
assert_file_exists <- function(fp) {
  if (!file.exists(fp)) {
    stop(sprintf("Cannot find file: %s", fp))
  }
}
```

```
lapply(X = input_data, FUN = assert_file_exists)
```

```
$sivep_2020
NULL

$sivep_2021
NULL

$socio_econ_2010
NULL

$vaccination_dates
NULL

$vaccination_coverage
NULL
```

If you want to check that the files you have match the ones used, here are some checksums (which can be obtained with `sha256sum *` from within `data/`.

```
9116d21e84b8ab4d4e8b2437644248618dc39affc7f6101d8ac7c631950c2e8e  agesex_coverage_muni_new.csv
7a5a6d07ab0beadd1c015d087802192aa02a227fad72ab33d8e0299600db29b0  census2010_muni_covariates.csv
e7db11cdf762b65b51df22ab4a71bf6f57f20d503ec568f34788c62bd5e0073f  dates_50.rds
2ee48a45bb88d3b78fa9c5571918d5bce23aa135fc3234ff1e1cc3836cfb4f32  sivep_20092021.csv
4c7bfe45057871a354ef00ab7a905b1daafff844dbd0d89045f8ed8c5f06c67a  sivep_2020.csv
```

Satellite cities and excluded municipality codes

The following `aggregated_satellites` function is taken from the preprocessing script.

> We exclude the municipality with code 431453 as it has a transient existence and 999999 since it is a malformed value. Since the codes are variously integers or characters we will filter these out on a case by case basis.
>
> Municipality codes starting with 53 are cities from DF. They are cities but not municipalities (they are called satellite cities). We substitute Brasilia's code (530010) for all of them. The following function helps with this.

```
aggregated_satellites <- function(muni_codes) {
  code_class <- class(muni_codes)
```


Li SL, *et al. BMJ Open* 2024; 14:e076354. doi: 10.1136/bmjopen-2023-076354

```
  stopifnot(
    is.element(el = code_class, set = c("integer", "character"))
  )
  if (code_class == "integer") {
    ifelse(round(muni_codes / 10000) == 53, 530010, muni_codes)
  } else {
    gsub(x = muni_codes,
         pattern = "53[0-9]{4}",
         replacement = "530010")
  }
}
```

Socio-economic (census) data

Remove the final digit from the municipality code associated with the municipality. The first digits are sufficient to uniquely identify them.

This dataset contains the measurements of the following quantities:

- `unemployed`,
- `informal`,
- `edu_primary_lower`,
- `avg_household_income_capita`,
- and `patient_address_muni_code` which is used for the join.

```
socio_econ_df <-
  input_data$socio_econ_2010 |>
  read_csv() |>
  mutate(patient_address_muni_code = aggregated_satellites(
           gsub("[0-9]$", "", code_muni)
         )) |>
  filter(patient_address_muni_code != "431453",
         patient_address_muni_code != "999999") |>
  select(-code_muni)
```

```
Rows: 5565 Columns: 5
-- Column specification --------------------------------------------------------
Delimiter: ","
dbl (5): code_muni, unemployed, informal, edu_primary_lower, avg_household_i...
```


Li SL, *et al. BMJ Open* 2024; 14:e076354. doi: 10.1136/bmjopen-2023-076354

```
i Use `spec()` to retrieve the full column specification for this data.
i Specify the column types or set `show_col_types = FALSE` to quiet this message.
```

Vaccination campaign data

Because in the preprocessing we used integers to represent age ranges, and in this script we use strings describing the range, it is helpful to have a function to change from the integers to the strings

```
age_group_int_to_string <- function(age_groups) {
  case_when(age_groups == 19 ~ "ageunder20",
            age_groups == 20 ~ "age20to29",
            age_groups == 30 ~ "age30to39",
            age_groups == 40 ~ "age40to49",
            age_groups == 50 ~ "age50to59",
            age_groups == 60 ~ "age60to69",
            age_groups == 70 ~ "age70to79",
            age_groups == 80 ~ "age80_plus")
}
```

We can read the data of when the vaccination campaign reached half coverage (for an age group in a specific municipality) with the following and rename the columns to match the rest of the code.

```
vac_dates_df <-
  input_data$vaccination_dates |>
  readRDS() |>
  mutate(age_group = age_group_int_to_string(age_group),
         municipality_code = as.character(municipality_code)) |>
  rename(patient_address_muni_code = municipality_code,
         vac_start_date = half_vac_date)
```

N.b. since we already handled the questionable municipality codes in the preprocessing script we do not need to worry about this again.

Old preprocessing steps

Here is an older version of the code to do this processing step, but the values are coming out slightly different which is a bit concerning.

```
vac_dates_df <-
  "dates_50.rds" |>
  readRDS() |>
  filter(age.lower != 90) |>
  mutate(age_group = case_when(age.lower == "< 20" ~ "ageunder20",
                               age.lower == 20 ~ "age20to29",
                               age.lower == 30 ~ "age30to39",
                               age.lower == 40 ~ "age40to49",
                               age.lower == 50 ~ "age50to59",
                               age.lower == 60 ~ "age60to69",
                               age.lower == 70 ~ "age70to79",
                               age.lower == 80 ~ "age80_plus"),
         patient_address_muni_code = as.character(mun_vac)) |>
  rename(vac_start_date = t50) |>
  as.data.frame() |>
  select(age_group,
         patient_address_muni_code,
         vac_start_date)
```

We need to coerce this to a data frame towards the end because otherwise it complains that the grouping column `mun_vac` is no longer present, even though it is, we have just renamed it.

Vaccination coverage data

Because the vaccination coverage was calculated from estimates of population size it is possible that it takes values greater than `1.0`. When this happens we need to clip the values down to `1.0`.

```
vac_cov_df <-
  input_data$vaccination_coverage |>
  readRDS() |>
  mutate(age_group = age_group_int_to_string(age_group),
         municipality_code = as.character(municipality_code)) |>
  rename(patient_address_muni_code = municipality_code)
```

Old preprocessing steps

Here is an older version of the code to do this processing step:

```
vac_cov_df <-
  "data/agesex_coverage_muni_new.csv" |>
  read.csv() |>
  mutate(agesex_coverage = pmin(agesex_coverage, 1),
         patient_address_muni_code = as.character(patient_address_muni_code)) |>
  select(patient_address_muni_code,
         patient_sex,
         age_group,
         agesex_coverage)
```

SIVEP-Gripe dataset

Preprocessing function

There are a couple of transformations that we want to apply to both the 2020 and 2021 SIVEP-Gripe datasets, so we should abstract them into a function to do this. In the filter we have the following conditions:

- `CLASSI_FIN %in% c(4, 5, 9, NA)` to select the cases that are probably COVID-19. Note that an earlier version of the code excluded `9` even though they only make up a very small number of cases.
- `SEM_PRI >= 10` to only keep instances where the symptom onset is after epi-week 10.
- `SG_UF` means that we require the patient to have their address state known.

```
preprocess_sivep <- function(sivep_df) {
  sivep_df |>
    filter(CLASSI_FIN %in% c(4, 5, 9, NA),
           SEM_PRI >= 10,
           SG_UF != "") |>
    rename(patient_address_muni_code = CO_MUN_RES,
           patient_sex = CS_SEXO) |>
    mutate(date_symptoms = dmy(DT_SIN_PRI),
           died_boolean = case_when(EVOLUCAO == 2 ~ 1, TRUE ~ 0),
           age_group = case_when(
             NU_IDADE_N < 20 ~ "ageunder20",
             NU_IDADE_N  >= 20 & NU_IDADE_N < 30 ~ "age20to29",
             NU_IDADE_N  >= 30 & NU_IDADE_N < 40 ~ "age30to39",
             NU_IDADE_N  >= 40 & NU_IDADE_N < 50 ~ "age40to49",
             NU_IDADE_N  >= 50 & NU_IDADE_N < 60 ~ "age50to59",
             NU_IDADE_N  >= 60 & NU_IDADE_N < 70 ~ "age60to69",
             NU_IDADE_N  >= 70 & NU_IDADE_N < 80 ~ "age70to79",
```

```
              NU_IDADE_N  >= 80                   ~ "age80_plus"),
            patient_address_muni_code = aggregated_satellites(
              as.character(patient_address_muni_code)
            )) |>
     filter(patient_address_muni_code != "431453",
            patient_address_muni_code != "999999") |>
     select(age_group,
            date_symptoms,
            died_boolean,
            patient_address_muni_code,
            patient_sex)
}
```

N.b. we need to do the processing on the municipality codes again here because this is raw data that has not been processed at all yet.

Preparing the data

The only difference in the processing of these data is that for the 2021 dataset we limit ourselves to cases with a symptom onset before 7 September 2021. This preprocessing step takes about a minute on my laptop.

```
sivep_2020_df <-
  input_data$sivep_2020 |>
  read_delim(delim = ";", show_col_types = FALSE) |>
  preprocess_sivep()
```

```
Warning: One or more parsing issues, call `problems()` on your data frame for details,
e.g.:
  dat <- vroom(...)
  problems(dat)
```

```
sivep_2021_df <-
  input_data$sivep_2021 |>
  read_delim(delim = ";", show_col_types = FALSE) |>
  preprocess_sivep() |>
  filter(date_symptoms < ymd("2021-09-07"))
```

```
Warning: One or more parsing issues, call `problems()` on your data frame for details,
e.g.:
  dat <- vroom(...)
  problems(dat)
```

```
sivep_df <- rbind(sivep_2020_df, sivep_2021_df)
```

The data frames `sivep_2020_df` and `sivep_2021_df` are about 50Mb each, this isn't a huge strain on my laptop, but since I have managed to crash R using these data before, I think it is probably a good idea to remove them since they are no longer needed.

```
rm(sivep_2020_df)
rm(sivep_2021_df)
gc()
```

```
           used  (Mb) gc trigger   (Mb)  max used   (Mb)
Ncells   985279  52.7    2178226  116.4   1781855   95.2
Vcells 60690315 463.1  395988225 3021.2 641652642 4895.5
```

Combined dataframe

We want to be able to filter the cases based on the state of the vaccination campaign so we need to join these data frames so we have the information linked properly.

First we start by joining the data frame that contains a Boolean reflecting if at least 50% of the age group in that municipality had been vaccinated.

```
tmp <-
  left_join(
    sivep_df,
    vac_dates_df,
    by = c("patient_address_muni_code", "age_group")
  ) |>
  mutate(vac_campaign = ifelse(date_symptoms < vac_start_date,
                               "pre", "post"))
stopifnot(!any(is.na(tmp)))
```

The next step is to join in the vaccination coverage as a proportion of the age group in the municipality on the date of symptom onset.

Li SL, *et al. BMJ Open* 2024; 14:e076354. doi: 10.1136/bmjopen-2023-076354

```
vac_cov_renamed_df <- rename(vac_cov_df, date_symptoms = date)
tmp2 <- left_join(
  tmp, vac_cov_renamed_df,
  by = c("age_group", "patient_address_muni_code", "date_symptoms")
)
rm(vac_cov_renamed_df)
pre_vac_mask <- tmp2$date_symptoms <= ymd("2021-01-16")
tmp2[pre_vac_mask, ]$proportion_vaccinated <- 0.0
```

Because there are not going to be any coverages from before the start of vaccination and there will be cases from this time, those records will get an `NA` for that variable so we need to fill those in with zero because that is the true value in that case. There are only 134 so it is a extremely small amount of corrupted data.

```
print(nrow( filter(vac_cov_df, date < as.Date("2021-01-17"))))
```

```
[1] 134
```

Finally we put all of the data into a single data frame so we can give it to the `glm` function later.

```
final_data_df <-
  left_join(tmp2, socio_econ_df, by = c("patient_address_muni_code")) |>
  mutate(age_group = relevel(as.factor(age_group), ref = "ageunder20"),
         vac_campaign = relevel(as.factor(vac_campaign), ref = "pre"))
```

We will save a copy of this data frame, not to use in the analysis, but so there is a record of it in case we need to check it later.

```
write.table(x = final_data_df,
            file = output_files$final_dataset,
            sep = ",",
            row.names = FALSE)
```

For future reference, the hash of this file is given below.

```
52b1bb1ec523b67f093d5e8bd9cb5821221729921e0cc104526f2d015f2b6325  out/final-mortality-dataset.csv
```


Li SL, *et al. BMJ Open* 2024; 14:e076354. doi: 10.1136/bmjopen-2023-076354

GLM

We remove the `vac_campaign` variable because it is highly colinear with the proportion vaccinated. Including it flips the effect of vaccination because of the colinearity, this is a cleaner model.

```
logit_fit <- glm(died_boolean ~ age_group +
                   proportion_vaccinated +
                   ## vac_campaign +
                   edu_primary_lower +
                   avg_household_income_capita +
                   unemployed,
                 family=binomial(link = "logit"),
                 data=final_data_df)
```

We want a summary table and the confidence intervals on the estimates for the manuscript so we should print these.

```
summary(logit_fit)
```

```
Call:
glm(formula = died_boolean ~ age_group + proportion_vaccinated +
    edu_primary_lower + avg_household_income_capita + unemployed,
    family = binomial(link = "logit"), data = final_data_df)

Coefficients:
                              Estimate Std. Error z value Pr(>|z|)
(Intercept)                 -3.011e+00  2.015e-02 -149.41   <2e-16 ***
age_groupage20to29           1.071e+00  1.863e-02   57.46   <2e-16 ***
age_groupage30to39           1.432e+00  1.619e-02   88.50   <2e-16 ***
age_groupage40to49           1.846e+00  1.553e-02  118.83   <2e-16 ***
age_groupage50to59           2.275e+00  1.525e-02  149.16   <2e-16 ***
age_groupage60to69           2.763e+00  1.519e-02  181.87   <2e-16 ***
age_groupage70to79           3.087e+00  1.525e-02  202.42   <2e-16 ***
age_groupage80_plus          3.367e+00  1.531e-02  219.90   <2e-16 ***
proportion_vaccinated       -1.992e-01  4.498e-03  -44.29   <2e-16 ***
edu_primary_lower           -1.465e+00  3.226e-02  -45.40   <2e-16 ***
avg_household_income_capita -2.831e-04  6.329e-06  -44.73   <2e-16 ***
unemployed                   8.289e-01  3.390e-02   24.45   <2e-16 ***
---
Signif. codes:  0 '***' 0.001 '**' 0.01 '*' 0.05 '.' 0.1 ' ' 1
```


Li SL, *et al. BMJ Open* 2024; 14:e076354. doi: 10.1136/bmjopen-2023-076354

```
(Dispersion parameter for binomial family taken to be 1)

    Null deviance: 2561369  on 2263367  degrees of freedom
Residual deviance: 2338211  on 2263356  degrees of freedom
  (481 observations deleted due to missingness)
AIC: 2338235

Number of Fisher Scoring iterations: 6
```

Since we want to be able to report confidence intervals on these parameters we should also print these values.

```
confint.default(logit_fit)
```

```
                                   2.5 %        97.5 %
(Intercept)                -3.0501491223 -2.9711607418
age_groupage20to29          1.0342129643  1.1072583584
age_groupage30to39          1.4007062008  1.4641515127
age_groupage40to49          1.8152694329  1.8761546475
age_groupage50to59          2.2454608015  2.3052587953
age_groupage60to69          2.7331468864  2.7926970935
age_groupage70to79          3.0569069010  3.1166836845
age_groupage80_plus         3.3371796759  3.3972028349
proportion_vaccinated      -0.2079929412 -0.1903628320
edu_primary_lower          -1.5280528065 -1.4015799558
avg_household_income_capita -0.0002955328 -0.0002707235
unemployed                  0.7624772639  0.8953497544
```

Since some of the socio-economic variables in particular are candidates for having a concerning level of colinearity, we should print the variance inflation factors (as computed by the `car` package). These values all come out small enough that we don't need to worry further.

```
vif(logit_fit)
```

```
                                GVIF Df GVIF^(1/(2*Df))
age_group                   1.074374  7        1.005137
proportion_vaccinated       1.073312  1        1.036008
edu_primary_lower           2.096518  1        1.447936
avg_household_income_capita 2.507043  1        1.583365
unemployed                  1.810226  1        1.345447
```


Li SL, *et al. BMJ Open* 2024; 14:e076354. doi: 10.1136/bmjopen-2023-076354

LaTeX table summarising the model fit

The following function is useful for generating a LaTeX table summarising the fitted model to avoid transcribing the estimates by hand.

The following helper function is used to make sure that when we are generating the table a sensible format is used for the display of numbers.

```
fmt_est_and_ci <- function(est, ci_min, ci_max) {
  sci_mask <-
    abs(pmin(est, ci_min, ci_max)) < 0.001 | abs(pmax(est, ci_min, ci_max)) > 1000
  ifelse(sci_mask,
         sprintf("%.3e (%.3e, %.3e)", est, ci_min, ci_max),
         sprintf("%.3f (%.3f, %.3f)", est, ci_min, ci_max))
}
```

We can now use that function in the definition of the function that makes the `xtable` object.

```
glm_fit_as_xtable <- function(fit, ...) {

  ns <- c(var = "Variable",
          est_and_ci = "Estimate and CI (95%)",
          sig = "Significance",
          p_val = "Pr(>|z|)")

  fs_df <- as.data.frame(summary(fit)$coefficient)[, c(1,4)]

  tmp <- confint.default(fit)
  stopifnot(ns['p_val'] %in% names(fs_df))
  fs_df[[ns['var']]] <- rownames(fs_df)
  fs_df[[ns['est_and_ci']]] <-
    fmt_est_and_ci(fs_df$Estimate, tmp[,1], tmp[,2])
  fs_df[[ns['sig']]] <-
    ifelse(fs_df[[ns['p_val']]] < 0.05 , "*", "")

  rownames(fs_df) <- NULL

  # Return xtable object
  return(xtable(fs_df[ns[c(1,2,3)]],
                align = c("l", "l", "r", "c"),
                ...))
}
```


Li SL, et al. BMJ Open 2024; 14:e076354. doi: 10.1136/bmjopen-2023-076354

Then we can use this to write the table to disk.

```
print.xtable(glm_fit_as_xtable(logit_fit),
             include.rownames = FALSE,
             type = "latex",
             file = output_files$final_model_table)
```

Session information

To compile this document, the following command could be used:

```
quarto render analysis-mortality.qmd
```

We should record the session information for posterity.

```
sessionInfo()
```

```
R version 4.3.0 (2023-04-21)
Platform: x86_64-pc-linux-gnu (64-bit)
Running under: Ubuntu 22.04.3 LTS

Matrix products: default
BLAS:   /usr/local/lib/R/lib/libRblas.so
LAPACK: /usr/lib/x86_64-linux-gnu/lapack/liblapack.so.3.10.0

locale:
 [1] LC_CTYPE=en_AU.UTF-8       LC_NUMERIC=C
 [3] LC_TIME=en_AU.UTF-8        LC_COLLATE=en_AU.UTF-8
 [5] LC_MONETARY=en_AU.UTF-8    LC_MESSAGES=en_AU.UTF-8
 [7] LC_PAPER=en_AU.UTF-8       LC_NAME=C
 [9] LC_ADDRESS=C               LC_TELEPHONE=C
[11] LC_MEASUREMENT=en_AU.UTF-8 LC_IDENTIFICATION=C

time zone: Australia/Melbourne
tzcode source: system (glibc)

attached base packages:
[1] stats     graphics  grDevices utils     datasets  methods   base

other attached packages:
```


Li SL, *et al. BMJ Open* 2024; 14:e076354. doi: 10.1136/bmjopen-2023-076354

```
[1] xtable_1.8-4    car_3.1-2       carData_3.0-5   lubridate_1.9.2
[5] readr_2.1.4     dplyr_1.1.2

loaded via a namespace (and not attached):
 [1] crayon_1.5.2      vctrs_0.6.3       cli_3.6.1         knitr_1.43
 [5] rlang_1.1.1       xfun_0.39         generics_0.1.3    jsonlite_1.8.7
 [9] bit_4.0.5         glue_1.6.2        htmltools_0.5.5   hms_1.1.3
[13] fansi_1.0.4       rmarkdown_2.23    abind_1.4-5       evaluate_0.21
[17] tibble_3.2.1      tzdb_0.4.0        fastmap_1.1.1     yaml_2.3.7
[21] lifecycle_1.0.3   compiler_4.3.0    timechange_0.2.0  pkgconfig_2.0.3
[25] rstudioapi_0.15.0 digest_0.6.33     R6_2.5.1          tidyselect_1.2.0
[29] utf8_1.2.3        parallel_4.3.0    vroom_1.6.3       pillar_1.9.0
[33] magrittr_2.0.3    withr_2.5.0       bit64_4.0.5       tools_4.3.0
```


Li SL, *et al. BMJ Open* 2024; 14:e076354. doi: 10.1136/bmjopen-2023-076354
