# Supplementary material for: The Brazilian COVID-19 vaccination campaign: a modelling analysis of sociodemographic factors on uptake
Source: BMJ Open. 2024 Jan 16;14(1):e076354. doi: 10.1136/bmjopen-2023-076354 (PMC10806735; doi:10.1136/bmjopen-2023-076354)
Supplement: Supplementary data [file bmjopen-2023-076354supp002.pdf]

Hospitalisation

Negative binomial GLM of hospitalisation

2023-10-27

Table of contents

Packages . . . 1
Data files . . . 2
  Output files . . . 2
  Input files . . . 2
  Check data files exist . . . 2
(Previous) mortality analysis data . . . 3
(Previous) population data . . . 4
Aggregated socio-economic data . . . 5
Combined dataframe . . . 8
GLM . . . 10
  (Quasi-)Poisson regression . . . 11
  Negative binomial . . . 13
  LaTeX table summarising the model fit . . . 15
Session information . . . 16

Packages

```
library(MASS)
library(dplyr)
library(readr)
library(lubridate)
library(car)
library(xtable)
```


Li SL, et al. BMJ Open 2024; 14:e076354. doi: 10.1136/bmjopen-2023-076354

- The `readr` package provides a function, `read_delim` that is faster than the built-in `read.csv` function and provides a progress bar.
- The `car` package provides the `vif` function which we use to check for a concerning about of covariation between the covariates of our model.
- The `MASS` package is needed for the negative binomial GLM model.
- The `xtable` package is needed to make a LaTeX table summarising the final model fit.

Data files

Output files

```
output_files <- list(
  final_dataset = "out/final-hospitalisation-dataset.csv",
  final_nb_model_table = "out/final-hospitalisation-model.tex"
)
```

Input files

Data files live in `data/`. Note that we will use the data set prepared for the hospitalisation analysis since this is still at the individual level and this saves us a non-trivial amount of preprocessing.

Note the `pop_estimates_2020_muni.csv` came from the covariates ZIP XSLX and contains municipality level population estimates.

```
input_data <- list(
  socio_econ_2010 = "data/census2010_muni_covariates.csv",
  mortality_dataset = "out/final-mortality-dataset.csv",
  population_data = "out/cleaned-population.rds"
  )
```

Check data files exist

```
assert_file_exists <- function(fp) {
  if (!file.exists(fp)) {
    stop(sprintf("Cannot find file: %s", fp))
  }
  return(0)
```


Li SL, *et al. BMJ Open* 2024; 14:e076354. doi: 10.1136/bmjopen-2023-076354

```r
}

lapply(X = input_data, FUN = assert_file_exists)
```

```
$socio_econ_2010
[1] 0

$mortality_dataset
[1] 0

$population_data
[1] 0
```

If you want to check that the files you have match the ones used, here are some checksums (which can be obtained with `sha256sum *` from within `data/`.

```
9116d21e84b8ab4d4e8b2437644248618dc39affc7f6101d8ac7c631950c2e8e  agesex_coverage_muni_new.csv
7a5a6d07ab0beadd1c015d087802192aa02a227fad72ab33d8e0299600db29b0  census2010_muni_covariates.csv
e7db11cdf762b65b51df22ab4a71bf6f57f20d503ec568f34788c62bd5e0073f  dates_50.rds
2ee48a45bb88d3b78fa9c5571918d5bce23aa135fc3234ff1e1cc3836cfb4f32  sivep_20092021.csv
4c7bfe45057871a354ef00ab7a905b1daafff844dbd0d89045f8ed8c5f06c67a  sivep_2020.csv
```

For the population sizes in each of the municipalities there is `covariates.zip` in `data/`

```
e06625739f8c0a3e41c41a23a82a4cb3c6ca51782872a12d839d12483ecd7229  covariates.zip
```

which contains an XLSX which appears to have population sizes mapped to municipality codes. We save to CSV in `out/` which has the hash

```
bb947d91b30496b6252d4bee7ef6ff98eeef48bbc3bbd445068455a56b82275e  pop_estimates_2020_muni.csv
```

(Previous) mortality analysis data

Because we don't want to do all the preprocessing from the mortality analysis again, we can just read in the data set that we prepared in that analysis. We do remove the socio economic variables so that they cannot accidentally be used instead of the other versions of these which are aggregated up to the state level below.

Li SL, *et al. BMJ Open* 2024; 14:e076354. doi: 10.1136/bmjopen-2023-076354

```
mortality_df <- input_data$mortality_dataset |>
  read_csv(show_col_types = FALSE) |>
  mutate(patient_address_muni_code = as.character(patient_address_muni_code)) |>
  select(-died_boolean,
         -patient_sex,
         -proportion_vaccinated,
         -vac_campaign,
         -unemployed,
         -informal,
         -edu_primary_lower,
         -avg_household_income_capita)
```

(Previous) population data

We redefine this function because we need it to get the population data into a more convenient format.

```
age_group_int_to_string <- function(age_groups) {
  case_when(age_groups == 19 ~ "ageunder20",
            age_groups == 20 ~ "age20to29",
            age_groups == 30 ~ "age30to39",
            age_groups == 40 ~ "age40to49",
            age_groups == 50 ~ "age50to59",
            age_groups == 60 ~ "age60to69",
            age_groups == 70 ~ "age70to79",
            age_groups == 80 ~ "age80_plus")
}
```

The state code is the first two digits of the six digits used to identify the municipality. Rather than process the population data again, we just read this in from the RDS saved in the previous processing.

```
pop_df <-
  input_data$population_data |>
  readRDS() |>
  mutate(muni_code = as.character(muni_code),
         state_code = gsub("[0-9]{4}$", "", muni_code),
         age_group = age_group_int_to_string(grouped_age)) |>
  select(muni_code,
         age_group,
```


Li SL, et al. BMJ Open 2024; 14:e076354. doi: 10.1136/bmjopen-2023-076354

```
          population,
          state_code)
```

N.b. Note that this dataframe keeps the population values age structured.

Aggregated socio-economic data

We start be reading in and setting up the socio economic data at the municipality level, including the state population size. Including the population size here makes it easier to do a state level aggregation later.

```
tmp <- input_data$socio_econ_2010 |>
  read_csv(show_col_types = FALSE) |>
  mutate(muni_code = gsub("[0-9]{1}$", "", as.character(code_muni))) |>
  select(-code_muni)

tmp_muni_lvl_socio_econ_df <- left_join(pop_df, tmp, by = "muni_code")
rm(pop_df)
rm(tmp)
gc()
```

```
          used (Mb) gc trigger  (Mb) max used  (Mb)
Ncells   892917 47.7    1710237  91.4  1349802  72.1
Vcells 10990195 83.9   36463450 278.2 31318311 239.0
```

Some of the municipalities are missing socio-economic data, but this only happens for a small number (four) of states, so in these instances we will replace the missing values by the weighted state average.

The following painful loop goes across the states with a municipality with missing data and fills in the missing values with the weighted average of the value of that variable across the other municipalities for which the data is available. It also prints out a description of how many records it is modifying which comes in at a very small proportion.

```
for (state in unique(tmp_muni_lvl_socio_econ_df$state_code)) {
  state_mask <- tmp_muni_lvl_socio_econ_df$state_code == state
  state_df <- tmp_muni_lvl_socio_econ_df[state_mask, ]
  if (any(is.na(state_df))) {
    print(sprintf("working on state %s", state))
    na_unemployed_mask <- is.na(tmp_muni_lvl_socio_econ_df$unemployed)
```

```
    na_informal_mask <- is.na(tmp_muni_lvl_socio_econ_df$informal)
    na_edu_primary_lower_mask <- is.na(tmp_muni_lvl_socio_econ_df$edu_primary_lower)
    na_income_mask <- is.na(tmp_muni_lvl_socio_econ_df$avg_household_income_capita)

    tmp <- state_df |>
      group_by(muni_code) |>
      summarise(muni_pop = sum(population),
                unq_unemployed = unique(unemployed),
                unq_informal = unique(informal),
                unq_edu_primary_lower = unique(edu_primary_lower),
                unq_income = unique(avg_household_income_capita))

    wm_unemployed <- weighted.mean(x = tmp$unq_unemployed,
                                   w = tmp$muni_pop,
                                   na.rm = TRUE)
    wm_informal <- weighted.mean(x = tmp$unq_informal,
                                 w = tmp$muni_pop,
                                 na.rm = TRUE)
    wm_edu_primary_lower <- weighted.mean(x = tmp$unq_edu_primary_lower,
                                          w = tmp$muni_pop,
                                          na.rm = TRUE)
    wm_income <- weighted.mean(x = tmp$unq_income,
                               w = tmp$muni_pop,
                               na.rm = TRUE)

    tmp_muni_lvl_socio_econ_df[state_mask & na_unemployed_mask,]$unemployed <- wm_unemployed
    print(sprintf("  filling in %d missing values with %f for unemployed",
                  sum(state_mask & na_unemployed_mask), wm_unemployed))
    tmp_muni_lvl_socio_econ_df[state_mask & na_informal_mask,]$informal <- wm_informal
    print(sprintf("  filling in %d missing values with %f for informal",
                  sum(state_mask & na_informal_mask), wm_informal))
    tmp_muni_lvl_socio_econ_df[state_mask & na_edu_primary_lower_mask,]$edu_primary_lower <- wm_e
    print(sprintf("  filling in %d missing values with %f for edu_primary_lower",
                  sum(state_mask & na_edu_primary_lower_mask), wm_edu_primary_lower))
    tmp_muni_lvl_socio_econ_df[state_mask & na_income_mask,]$avg_household_income_capita <- wm_ir
    print(sprintf("  filling in %d missing values with %f for for avg_household_income_capita",
                  sum(state_mask & na_income_mask), wm_income))
    }
}
```

```
[1] "working on state 15"
```

```
[1] "  filling in 8 missing values with 0.235284 for unemployed"
[1] "  filling in 8 missing values with 0.092493 for informal"
[1] "  filling in 8 missing values with 0.307297 for edu_primary_lower"
[1] "  filling in 8 missing values with 424.206534 for for avg_household_income_capita"
[1] "working on state 42"
[1] "  filling in 16 missing values with 0.112102 for unemployed"
[1] "  filling in 16 missing values with 0.038951 for informal"
[1] "  filling in 16 missing values with 0.289139 for edu_primary_lower"
[1] "  filling in 16 missing values with 979.963089 for for avg_household_income_capita"
[1] "working on state 43"
[1] "  filling in 8 missing values with 0.137998 for unemployed"
[1] "  filling in 8 missing values with 0.050790 for informal"
[1] "  filling in 8 missing values with 0.280631 for edu_primary_lower"
[1] "  filling in 8 missing values with 947.221227 for for avg_household_income_capita"
[1] "working on state 50"
[1] "  filling in 8 missing values with 0.199913 for unemployed"
[1] "  filling in 8 missing values with 0.062054 for informal"
[1] "  filling in 8 missing values with 0.263784 for edu_primary_lower"
[1] "  filling in 8 missing values with 788.500330 for for avg_household_income_capita"
```

```
stopifnot(!any(is.na(tmp_muni_lvl_socio_econ_df)))
```

These values are very similar to the country wide unweighted averages shown by the following:

```
tmp_muni_lvl_socio_econ_df |>
  select(unemployed,
         informal,
         edu_primary_lower,
         avg_household_income_capita ) |>
  colMeans()
```

```
         unemployed                    informal
         0.24181327                  0.06512755
  edu_primary_lower avg_household_income_capita
         0.36311907                484.56685182
```

Since we do not have the socio economic data at an age group level, we need to aggregate this up to be able to work at the state level. We will do this respecting the population sizes in each municipality.

Li SL, *et al. BMJ Open* 2024; 14:e076354. doi: 10.1136/bmjopen-2023-076354

```
tmp_state_age_socio_econ_df <-
  tmp_muni_lvl_socio_econ_df |>
  group_by(muni_code, age_group) |>
  summarise(muni_pop = sum(population),
            state_code = unique(state_code),
            unemployed = unique(unemployed),
            informal = unique(informal),
            edu_primary_lower = unique(edu_primary_lower),
            avg_household_income_capita = unique(avg_household_income_capita),
            .groups = "drop") |>
  group_by(state_code, age_group) |>
  summarise(state_pop = sum(muni_pop),
            s_unemployed = weighted.mean(x = unemployed,
                                         w = muni_pop),
            s_informal = weighted.mean(x = informal,
                                       w = muni_pop),
            s_edu_primary_lower = weighted.mean(x = edu_primary_lower,
                                                w = muni_pop),
            s_avg_household_income_capita = weighted.mean(x = avg_household_income_capita,
                                                          w = muni_pop),
            .groups = "keep")
```

Combined dataframe

When combining up to state level we compute the average vaccination start date across each age group within each state. This will introduce some bias but it captures the intended effect. We want to have a dataframe with one record for each hospitalisation but it also needs to have the age group and a shared vaccination start date for this cohort so we have a sensible shared pre/post vaccination divide.

```
disaggregated_data_df <-
  left_join(
    mortality_df,
    rename(tmp_muni_lvl_socio_econ_df,
           patient_address_muni_code = muni_code),
    by = c("patient_address_muni_code", "age_group")
  ) |>
  group_by(age_group, state_code) |>
  summarise(date_symptoms = date_symptoms,
            patient_address_muni_code = patient_address_muni_code,
```

```
            vac_start_date = rep(mean(vac_start_date), length(date_symptoms))) |>
  mutate(vac_campaign =  ifelse(date_symptoms < vac_start_date,
                                "pre", "post"))
```

```
Warning: Returning more (or less) than 1 row per `summarise()` group was deprecated in
dplyr 1.1.0.
i Please use `reframe()` instead.
i When switching from `summarise()` to `reframe()`, remember that `reframe()`
  always returns an ungrouped data frame and adjust accordingly.
```

```
`summarise()` has grouped output by 'age_group', 'state_code'. You can override
using the `.groups` argument.
```

N.b. The populations in this dataframe are the municipality populations. And there are some municipalities that are appear in the socio economic data but not in the case data so these get removed in the left join above. So we do not rely on this join to produce a sensible population size.

N.b. Don't worry about the warning here, provided you are using a version of `dplyr` that has not removed the deprecated function this should work fine.

Now we need to aggregate these hospitalisations.

```
aggregated_data_df <-
  disaggregated_data_df |>
  group_by(age_group,
           ## patient_address_muni_code,
           state_code,
           vac_campaign) |>
  summarise(num_hosp = length(patient_address_muni_code),
            vac_start_date = unique(vac_start_date),
            .groups = "drop") |>
  left_join(tmp_state_age_socio_econ_df, by = c("state_code", "age_group")) |>
  mutate(age_group = relevel(as.factor(age_group), ref = "ageunder20"))
```

N.b. there are no "post" vaccination records for the under 20s because vaccination hadn't reached the halfway mark by the end of the study period. As a result, quick attempts to count the population size by grouping by state codes will give a under estimate, you need to select just the records from the pre-vaccination time epoch.

Li SL, *et al. BMJ Open* 2024; 14:e076354. doi: 10.1136/bmjopen-2023-076354

Finally, we need to include the duration across which we are aggregating so that we can offset by both the population and number of people so that the regression gives us the rate of hospitalisations per person per day.

```
pre_vac_mask <- aggregated_data_df$vac_campaign == "pre"
final_data_df <- aggregated_data_df
final_data_df$obs_duration <- NA
tmp <- ymd(final_data_df$vac_start_date)
final_data_df[pre_vac_mask,]$obs_duration <-
  as.numeric(tmp[pre_vac_mask] - ymd("2020-03-01"))
final_data_df[!pre_vac_mask,]$obs_duration <-
  as.numeric(ymd("2021-09-06") - tmp[!pre_vac_mask])
```

Because it makes more sense to talk about the change in rate in response to substantial vaccination levels, we make the "pre" interval as the reference.

```
final_data_df$vac_campaign <- relevel(as.factor(final_data_df$vac_campaign), ref = "pre")
```

We will save a copy of this data frame, not to use in the analysis, but so there is a record of it in case we need to check it later.

```
write.table(x = final_data_df,
            file = output_files$final_dataset,
            sep = ",",
            row.names = FALSE)
```

GLM

The GLM uses a log-link function with the (log) population size and the (log) observation duration as offsets. This means that the expected number of hospitalisations, μ is modelled by

$$\mu = N_{\text{pop}} T_{\text{days}} \exp\{\mathbf{X}\beta\}$$

where N_{pop} is the relevant population size, T_{days} is the number of days during which the hospitalisations were observed and $\mathbf{X}\beta$ is a linear combination of the remaining covariates.

The advantage of including these variables as offsets in this way is that we get a *daily per capita* rate out of the model.

Li SL, *et al. BMJ Open* 2024; 14:e076354. doi: 10.1136/bmjopen-2023-076354

(Quasi-)Poisson regression

Because unemployment has substantial correlations with the average household income variable, we remove this to avoid issues with colinearity. This was discovered via the presence of a large VIF.

```r
q_poisson_fit <- glm(num_hosp ~ age_group +
                       vac_campaign +
                       s_avg_household_income_capita +
                       s_edu_primary_lower +
                       ## s_unemployed +
                       offset(log(obs_duration)) +
                       offset(log(state_pop)),
                     data = final_data_df,
                     family = quasipoisson())
```

```r
summary(q_poisson_fit)
```

```

Call:
glm(formula = num_hosp ~ age_group + vac_campaign + s_avg_household_income_capita +
    s_edu_primary_lower + offset(log(obs_duration)) + offset(log(state_pop)),
    family = quasipoisson(), data = final_data_df)

Coefficients:
                                Estimate Std. Error t value Pr(>|t|)
(Intercept)                   -1.251e+01  2.225e-01 -56.199  < 2e-16 ***
age_groupage20to29             1.285e-01  7.827e-02   1.642  0.10139
age_groupage30to39             9.107e-01  6.406e-02  14.218  < 2e-16 ***
age_groupage40to49             1.424e+00  6.013e-02  23.685  < 2e-16 ***
age_groupage50to59             1.849e+00  5.828e-02  31.722  < 2e-16 ***
age_groupage60to69             2.201e+00  5.877e-02  37.453  < 2e-16 ***
age_groupage70to79             2.657e+00  6.057e-02  43.867  < 2e-16 ***
age_groupage80_plus            3.196e+00  6.260e-02  51.061  < 2e-16 ***
vac_campaignpost              -9.467e-02  3.530e-02  -2.682  0.00763 **
s_avg_household_income_capita  5.901e-04  8.338e-05   7.077 6.82e-12 ***
s_edu_primary_lower           -7.536e-01  5.965e-01  -1.263  0.20722
---
Signif. codes:  0 '***' 0.001 '**' 0.01 '*' 0.05 '.' 0.1 ' ' 1

(Dispersion parameter for quasipoisson family taken to be 381.1353)
```


Li SL, et al. BMJ Open 2024; 14:e076354. doi: 10.1136/bmjopen-2023-076354

```
    Null deviance: 2304187  on 403  degrees of freedom
Residual deviance:  151951  on 393  degrees of freedom
AIC: NA

Number of Fisher Scoring iterations: 4
```

```
confint.default(q_poisson_fit)
```

```
                                    2.5 %        97.5 %
(Intercept)                  -12.942330792 -1.207001e+01
age_groupage20to29            -0.024887847  2.819238e-01
age_groupage30to39             0.785202963  1.036297e+00
age_groupage40to49             1.306210093  1.541899e+00
age_groupage50to59             1.734511728  1.962961e+00
age_groupage60to69             2.085776824  2.316136e+00
age_groupage70to79             2.538251013  2.775678e+00
age_groupage80_plus            3.073515518  3.318888e+00
vac_campaignpost              -0.163856323 -2.548826e-02
s_avg_household_income_capita  0.000426641  7.534905e-04
s_edu_primary_lower           -1.922664681  4.155395e-01
```

Since some of the socio-economic variables in particular are candidates for having a concerning level of colinearity, we should print the variance inflation factors (as computed by the `car` package). These values all come out small enough that we don't need to worry further.

```
vif(q_poisson_fit)
```

```
                                  GVIF Df GVIF^(1/(2*Df))
age_group                     1.109441  7        1.007446
vac_campaign                  1.076342  1        1.037469
s_avg_household_income_capita 2.847989  1        1.687599
s_edu_primary_lower           2.842682  1        1.686026
```

However, we don't want to use the Poisson model so we will also fit a negative binomial model.

Li SL, *et al. BMJ Open* 2024; 14:e076354. doi: 10.1136/bmjopen-2023-076354

Negative binomial

To address the substantial amount of overdispersion in the dataset, while sticking to a likelihood framework, we will use the negative binomial distribution to model.

Recall that negative binomial GLM regression is provided by the `MASS` package.

```
nb_fit <- glm.nb(num_hosp ~ age_group +
                   vac_campaign +
                   s_avg_household_income_capita +
                   s_edu_primary_lower +
                   ## s_unemployed +
                   offset(log(obs_duration)) +
                   offset(log(state_pop)),
                 data = final_data_df)
```

```
summary(nb_fit)
```

```
Call:
glm.nb(formula = num_hosp ~ age_group + vac_campaign + s_avg_household_income_capita +
    s_edu_primary_lower + offset(log(obs_duration)) + offset(log(state_pop)),
    data = final_data_df, init.theta = 5.432893292, link = log)

Coefficients:
                                Estimate Std. Error z value Pr(>|z|)
(Intercept)                   -1.311e+01  2.553e-01 -51.361  < 2e-16 ***
age_groupage20to29             9.368e-02  1.048e-01   0.894    0.371
age_groupage30to39             8.491e-01  1.040e-01   8.163 3.26e-16 ***
age_groupage40to49             1.400e+00  1.038e-01  13.481  < 2e-16 ***
age_groupage50to59             1.917e+00  1.038e-01  18.469  < 2e-16 ***
age_groupage60to69             2.493e+00  1.037e-01  24.032  < 2e-16 ***
age_groupage70to79             3.008e+00  1.037e-01  29.005  < 2e-16 ***
age_groupage80_plus            3.558e+00  1.038e-01  34.291  < 2e-16 ***
vac_campaignpost              -3.732e-01  4.460e-02  -8.368  < 2e-16 ***
s_avg_household_income_capita  8.038e-04  1.032e-04   7.786 6.92e-15 ***
s_edu_primary_lower            5.821e-01  6.858e-01   0.849    0.396
---
Signif. codes:  0 '***' 0.001 '**' 0.01 '*' 0.05 '.' 0.1 ' ' 1

(Dispersion parameter for Negative Binomial(5.4329) family taken to be 1)
```


Li SL, *et al. BMJ Open* 2024; 14:e076354. doi: 10.1136/bmjopen-2023-076354

```
    Null deviance: 3054.42  on 403  degrees of freedom
Residual deviance:  427.16  on 393  degrees of freedom
AIC: 6446.3

Number of Fisher Scoring iterations: 1

              Theta:  5.433
          Std. Err.:  0.387

 2 x log-likelihood:  -6422.323
```

```
confint.default(nb_fit)
```

```
                                   2.5 %        97.5 %
(Intercept)                  -1.361435e+01 -12.613486653
age_groupage20to29           -1.116569e-01   0.299016717
age_groupage30to39            6.452518e-01   1.053000486
age_groupage40to49            1.196463e+00   1.603540085
age_groupage50to59            1.713302e+00   2.120111970
age_groupage60to69            2.289520e+00   2.696123827
age_groupage70to79            2.805180e+00   3.211764274
age_groupage80_plus           3.354731e+00   3.761466202
vac_campaignpost             -4.606533e-01  -0.285816886
s_avg_household_income_capita  6.014233e-04   0.001006087
s_edu_primary_lower          -7.620310e-01   1.926271435
```

Since some of the socio-economic variables in particular are candidates for having a concerning level of colinearity, we should print the variance inflation factors (as computed by the `car` package). These values all come out small enough that we don't need to worry further.

```
vif(nb_fit)
```

```
                                  GVIF Df GVIF^(1/(2*Df))
age_group                     1.077055  7        1.005316
vac_campaign                  1.066380  1        1.032657
s_avg_household_income_capita 1.772904  1        1.331504
s_edu_primary_lower           1.785122  1        1.336084
```


Li SL, *et al. BMJ Open* 2024; 14:e076354. doi: 10.1136/bmjopen-2023-076354

LaTeX table summarising the model fit

The following function is useful for generating a LaTeX table summarising the fitted model to avoid transcribing the estimates by hand.

The following helper function is used to make sure that when we are generating the table a sensible format is used for the display of numbers.

```
fmt_est_and_ci <- function(est, ci_min, ci_max) {
  sci_mask <-
    abs(pmin(est, ci_min, ci_max)) < 0.001 | abs(pmax(est, ci_min, ci_max)) > 1000
  ifelse(sci_mask,
         sprintf("%.3e (%.3e, %.3e)", est, ci_min, ci_max),
         sprintf("%.3f (%.3f, %.3f)", est, ci_min, ci_max))
}
```

We can now use that function in the definition of the function that makes the `xtable` object.

```
glm_fit_as_xtable <- function(fit, ...) {

  ns <- c(var = "Variable",
          est_and_ci = "Estimate and CI (95%)",
          sig = "Significance",
          p_val = "Pr(>|z|)")

  fs_df <- as.data.frame(summary(fit)$coefficient)[, c(1,4)]

  tmp <- confint.default(fit)
  stopifnot(ns['p_val'] %in% names(fs_df))
  fs_df[[ns['var']]] <- rownames(fs_df)
  fs_df[[ns['est_and_ci']]] <-
    fmt_est_and_ci(fs_df$Estimate, tmp[,1], tmp[,2])
  fs_df[[ns['sig']]] <-
    ifelse(fs_df[[ns['p_val']]] < 0.05 , "*", "")

  rownames(fs_df) <- NULL

  # Return xtable object
  return(xtable(fs_df[ns[c(1,2,3)]],
                align = c("l", "l", "r", "c"),
                ...))
}
```

Then we can use this to write the table to disk.

```
print.xtable(glm_fit_as_xtable(nb_fit),
             include.rownames = FALSE,
             type = "latex",
             file = output_files$final_nb_model_table)
```

Session information

To compile this document, the following command could be used:

```
quarto render analysis-hospitalisation.qmd
```

We should record the session information for posterity.

```
sessionInfo()
```

```
R version 4.3.0 (2023-04-21)
Platform: x86_64-pc-linux-gnu (64-bit)
Running under: Ubuntu 22.04.3 LTS

Matrix products: default
BLAS:   /usr/local/lib/R/lib/libRblas.so
LAPACK: /usr/lib/x86_64-linux-gnu/lapack/liblapack.so.3.10.0

locale:
 [1] LC_CTYPE=en_AU.UTF-8       LC_NUMERIC=C
 [3] LC_TIME=en_AU.UTF-8        LC_COLLATE=en_AU.UTF-8
 [5] LC_MONETARY=en_AU.UTF-8    LC_MESSAGES=en_AU.UTF-8
 [7] LC_PAPER=en_AU.UTF-8       LC_NAME=C
 [9] LC_ADDRESS=C               LC_TELEPHONE=C
[11] LC_MEASUREMENT=en_AU.UTF-8 LC_IDENTIFICATION=C

time zone: Australia/Melbourne
tzcode source: system (glibc)

attached base packages:
[1] stats     graphics  grDevices utils     datasets  methods   base

other attached packages:
```

```
[1] xtable_1.8-4    car_3.1-2       carData_3.0-5   lubridate_1.9.2
[5] readr_2.1.4     dplyr_1.1.2     MASS_7.3-58.4

loaded via a namespace (and not attached):
 [1] bit_4.0.5         jsonlite_1.8.7    compiler_4.3.0    crayon_1.5.2
 [5] tidyselect_1.2.0  parallel_4.3.0    yaml_2.3.7        fastmap_1.1.1
 [9] R6_2.5.1          generics_0.1.3    knitr_1.43        tibble_3.2.1
[13] pillar_1.9.0      tzdb_0.4.0        rlang_1.1.1       utf8_1.2.3
[17] xfun_0.39         bit64_4.0.5       timechange_0.2.0  cli_3.6.1
[21] withr_2.5.0       magrittr_2.0.3    digest_0.6.33     vroom_1.6.3
[25] rstudioapi_0.15.0 hms_1.1.3         lifecycle_1.0.3   vctrs_0.6.3
[29] evaluate_0.21     glue_1.6.2        abind_1.4-5       fansi_1.0.4
[33] rmarkdown_2.23    tools_4.3.0       pkgconfig_2.0.3   htmltools_0.5.5
```


Li SL, *et al. BMJ Open* 2024; 14:e076354. doi: 10.1136/bmjopen-2023-076354
