# Supplementary material for: The Brazilian COVID-19 vaccination campaign: a modelling analysis of sociodemographic factors on uptake
Source: BMJ Open. 2024 Jan 16;14(1):e076354. doi: 10.1136/bmjopen-2023-076354 (PMC10806735; doi:10.1136/bmjopen-2023-076354)
Supplement: Supplementary data [file bmjopen-2023-076354supp003.pdf]

Supplementary Information: The Brazilian COVID-19 vaccination campaign: A modelling analysis of socio-demographic factors on uptake

Sabrina L. Li[1,2,*,=], Carlos A. Prete Jr[3,=], Alexander E. Zarebski[4,5,=], Andreza A. de Souza Santos[6], Ester C. Sabino[7], Vitor H. Nascimento[3], Chieh-Hsi Wu[8], and Jane P. Messina[1,9]

[1]School of Geography and the Environment, University of Oxford, Oxford, UK
[2]School of Geography, University of Nottingham, Nottingham, UK
[3]Department of Electronic Systems Engineering, University of São Paulo, São Paulo, Brazil
[4]Department of Biology, University of Oxford, Oxford, UK
[5]School of Mathematics and Statistics, University of Melbourne, Melbourne, Australia
[6]Oxford School of Global and Area Studies, Latin American Centre, University of Oxford, Oxford, UK
[7]Departamento de Molestias Infecciosas e Parasitarias & Instituto de Medicina Tropical da Faculdade de Medicina da Universidade de São Paulo, São Paulo, Brazil
[8]Mathematical Sciences, University of Southampton, Southampton, UK
[9]Oxford School of Global and Area Studies, University of Oxford, Oxford, UK
[=]These authors contributed equally
[*]Corresponding author: `Sabrina.Li@nottingham.ac.uk`

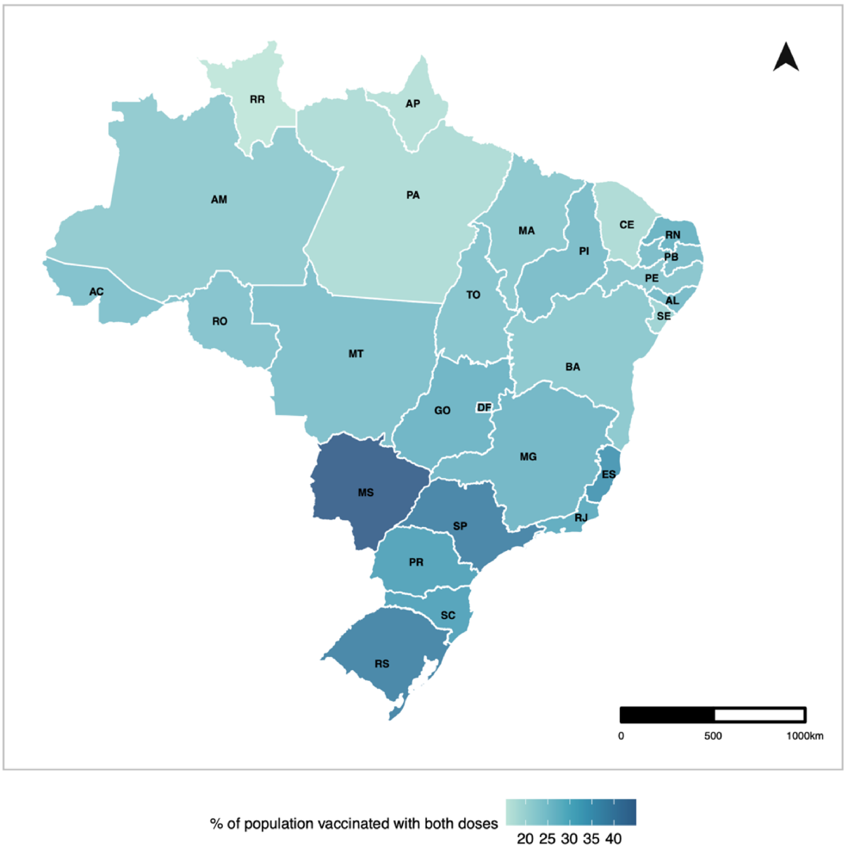

Figure S1: Proportion of population fully vaccinated.

Li SL, *et al. BMJ Open* 2024; 14:e076354. doi: 10.1136/bmjopen-2023-076354

Risk of COVID-19 death by age-group

The equation describing the log-odds of death among SARI patients is expressed as

$$\begin{aligned}\text{logit}(p_i) = \beta_0 &+ \beta_1 \cdot (\text{Age group of } i) \\ &+ \beta_2 \cdot (\text{Vaccination coverage in age group of } i \text{ in their municipality}) \\ &+ \beta_3 \cdot (\text{Low education attainment in municipality of } i) \\ &+ \beta_4 \cdot (\text{Average income per capita in municipality of } i) \\ &+ \beta_5 \cdot (\text{Unemployment in municipality of } i) + \epsilon_i,\end{aligned} \tag{1}$$

where $p_i = \mathbb{P}(Y_i = 1)$ is the log-odds of death where Y_i is an indicator variable for whether the ith SARI patient's outcome: dead = 1, and alive = 0. The β_j are the coefficients of the model and ϵ_i is the error term. For reference, the logit function is defined as $\text{logit}(p) = \log(p/(1-p))$.

As a form of sensitivity analysis, we fit the model at the individual level by including only patients that had a recorded vaccination status (e.g. vaccinated with at least one dose, or no vaccination). Here we included vaccination status as a binary indicator in the model to replace the age-group specific vaccination coverage. While this avoids the potential for an ecological fallacy (i.e. assuming population level vaccination levels are indicative of the individuals represented in the data), we cannot rule out the possibility that data may not be missing at random, which may introduce biases in the estimates.

The resulting parameter estimates from this model and their confidence intervals are given in Table S1.

Variable	Estimate and CI (95%)	Significance
Intercept	-3.011 (-3.050, -2.971)	*
Age group#: 20–29	1.071 (1.034, 1.107)	*
Age group: 30–39	1.432 (1.401, 1.464)	*
Age group: 40–49	1.846 (1.815, 1.876)	*
Age group: 50–59	2.275 (2.245, 2.305)	*
Age group: 60–69	2.763 (2.733, 2.793)	*
Age group: 70–79	3.087 (3.057, 3.117)	*
Age group: ≥ 80	3.367 (3.337, 3.397)	*
Proportion vaccinated	-0.199 (-0.208, -0.190)	*
Primary education or lower	-1.465 (-1.528, -1.402)	*
Income per capita (US$ PPP)	-3.930×10^{-4} (-4.101×10^{-4}, -3.757×10^{-4})	*
Unemployed	0.829 (0.762, 0.895)	*

Baseline age group for comparison is under 20 years old
* statistically significant p-value < 0.05

Table S1: Logistic regression model analysis — death risk of SARI patient given age group, age group-specific vaccination coverage, and socioeconomic indicators.

Li SL, *et al. BMJ Open* 2024; 14:e076354. doi: 10.1136/bmjopen-2023-076354

Risk of COVID-19 hospitalisation by age group

The equation describing the expected number of hospitalisations in this model is

$$\begin{aligned}\log(\mathbb{E}[Y_i]) &= \log(N_i) + \log(T_i) \\ &+ \beta_0 + \beta_1 \cdot (\text{Age group for } i) \\ &+ \beta_2 \cdot (\text{Campaign status indicator for age group of } i \text{ in that state}) \\ &+ \beta_3 \cdot (\text{Average income per capita in state of } i) \\ &+ \beta_4 \cdot (\text{Low education attainment in state of } i) + \epsilon_i\end{aligned} \quad (2)$$

where Y_i is the number of hospitalisations in a particular age group within a particular state during the period of time before (or after) approximately half of that age group was vaccinated in that state. The distribution of Y_i given the mean is assumed to follow a negative binomial distribution (with estimated dispersion parameter of 5.43).

The variables N_i and T_i represent the number of people in that age group in that state, and the number of days in that period respectively. These offset variables are included so that the result can be interpreted as a daily per capita risk.

The resulting parameter estimates from this model and their confidence intervals are given in Table S2. The state level average unemployment was not included as a covariate because it was strongly correlated with the over socio-economic variables (as measured by VIF). This colinearity was less substantial at the municipality level.

Variable	Estimate and CI (95%)	Significance
Intercept	-13.114 (-13.614, -12.613)	*
Age group#: 20–29	0.094 (-0.112, 0.299)	
Age group: 30–39	0.849 (0.645, 1.053)	*
Age group: 40–49	1.400 (1.196, 1.604)	*
Age group: 50–59	1.917 (1.713, 2.120)	*
Age group: 60–69	2.493 (2.290, 2.696)	*
Age group: 70–79	3.008 (2.805, 3.212)	*
Age group: ≥ 80	3.558 (3.355, 3.761)	*
Vaccination campaign (yes)	-0.373 (-0.461, -0.286)	*
Income per capita (US$ PPP)	11.157×10^{-4} (8.345×10^{-4}, 1.396×10^{-3})	*
Primary education or lower	0.582 (-0.762, 1.926)	

Baseline age group for comparison is under 20 years old
* statistically significant p-value < 0.05

Table S2: Negative binomial regression model analysis — hospitalisation risk given age group, socioeconomic indicators, and indication of age-group specific vaccination campaign

Li SL, *et al. BMJ Open* 2024; 14:e076354. doi: 10.1136/bmjopen-2023-076354
