# Supplementary material for: The Brazilian COVID-19 vaccination campaign: a modelling analysis of sociodemographic factors on uptake
Source: BMJ Open. 2024 Jan 16;14(1):e076354. doi: 10.1136/bmjopen-2023-076354 (PMC10806735; doi:10.1136/bmjopen-2023-076354)
Supplement: Author's manuscript [file bmjopen-2023-076354.draft_revisions.pdf]

BMJ Open

BMJ Open is committed to open peer review. As part of this commitment we make the peer review history of every article we publish publicly available.

When an article is published we post the peer reviewers' comments and the authors' responses online. We also post the versions of the paper that were used during peer review. These are the versions that the peer review comments apply to.

The versions of the paper that follow are the versions that were submitted during the peer review process. They are not the versions of record or the final published versions. They should not be cited or distributed as the published version of this manuscript.

BMJ Open is an open access journal and the full, final, typeset and author-corrected version of record of the manuscript is available on our site with no access controls, subscription charges or pay-per-view fees (http://bmjopen.bmj.com).

If you have any questions on BMJ Open's open peer review process please email info.bmjopen@bmj.com

BMJ Open

The Brazilian COVID-19 vaccination campaign: A modelling analysis of socio-demographic factors on uptake

Journal:	*BMJ Open*
Manuscript ID	bmjopen-2023-076354
Article Type:	Original research
Date Submitted by the Author:	05-Jun-2023
Complete List of Authors:	Li, Sabrina; University of Nottingham,<br>Prete Junior, Carlos Augusto; Universidade de São Paulo,<br>Zarebski, Alexander; Oxford University; The University of Melbourne<br>Santos, Andreza ; University of Oxford<br>Sabino, Ester; Universidade de São Paulo<br>Nascimento, Vitor; Universidade de Sao Paulo<br>Wu, Chieh-Hsi ; University of Southampton,<br>Messina, Jane; University of Oxford,
Keywords:	COVID-19, PUBLIC HEALTH, Health Equity

SCHOLARONE™
Manuscripts

I, the Submitting Author has the right to grant and does grant on behalf of all authors of the Work (as defined in the below author licence), an exclusive licence and/or a non-exclusive licence for contributions from authors who are: i) UK Crown employees; ii) where BMJ has agreed a CC-BY licence shall apply, and/or iii) in accordance with the terms applicable for US Federal Government officers or employees acting as part of their official duties; on a worldwide, perpetual, irrevocable, royalty-free basis to BMJ Publishing Group Ltd ("BMJ") its licensees and where the relevant Journal is co-owned by BMJ to the co-owners of the Journal, to publish the Work in this journal and any other BMJ products and to exploit all rights, as set out in our licence.

The Submitting Author accepts and understands that any supply made under these terms is made by BMJ to the Submitting Author unless you are acting as an employee on behalf of your employer or a postgraduate student of an affiliated institution which is paying any applicable article publishing charge ("APC") for Open Access articles. Where the Submitting Author wishes to make the Work available on an Open Access basis (and intends to pay the relevant APC), the terms of reuse of such Open Access shall be governed by a Creative Commons licence – details of these licences and which Creative Commons licence will apply to this Work are set out in our licence referred to above.

Other than as permitted in any relevant BMJ Author's Self Archiving Policies, I confirm this Work has not been accepted for publication elsewhere, is not being considered for publication elsewhere and does not duplicate material already published. I confirm all authors consent to publication of this Work and authorise the granting of this licence.

For peer review only

The Brazilian COVID-19 vaccination campaign: A modelling analysis of socio-demographic factors on uptake

Abstract

Background: The COVID-19 pandemic has caused over half a million deaths in Brazil, and public healthcare nearly collapsed. Vaccination differs between states and demographics. Dose shortages delayed access.

Methods: In this cross-sectional study, data were retrieved from the Brazilian Ministry of Health databases published since 17 January 2021, respectively. We developed a campaign optimality index to characterize inequality in vaccination access caused by age due to premature vaccination towards younger populations before older and vulnerable populations were fully vaccinated. We assessed geographical inequalities in full vaccination coverage and dose by age, sex, race, and socioeconomic status. Generalized linear regression was used to investigate the risk of death and hospitalization by age group, socioeconomic status, and vaccination coverage.

Results: Vaccination coverage is higher in the wealthier South and Southeast. Men, people of colour, and low- income groups were more likely to be only partially vaccinated due to missing or delaying a second dose. Vaccination started prematurely for age groups under 50 years and may have hindered uptake of older age groups. Vaccination coverage was associated with a lower risk of death, especially in older age groups (OR: 10.5–34.8, 95% CI: (10.2, 35.9)). Risk of hospitalisation was greater in areas with higher vaccination rates due to higher access to care and reporting.

Conclusions: Vaccination inequality persists between states, age and demographic groups despite increasing uptake. The association between hospitalisation rates and vaccination is attributed to preferential delivery to areas of greater transmission and access to healthcare.

For peer review only - http://bmjopen.bmj.com/site/about/guidelines.xhtml

Strengths and Limitations

- A key strength of this study is that data were collected from the Brazilian Ministry of Health's national immunization system which included information on every individual that participated in the vaccination programme.
- We combined spatial data on vaccination, cases on severe acute respiratory illnesses and confirmed COVID-19 cases (due to low rates of COVID-19 testing in Brazil), socioeconomic conditions, and recent population estimates from 2020 to account for excess COVID-19 deaths.
- We built a spatially explicit model to robustly infer vaccination uptake and health risk across Brazil.
- We developed a vaccination Campaign Optimality Index (COI) which describes how vaccination was rolled out, estimating the extent to which older groups were and were not prioritized during the campaign.
- A limitation is we were unable to retrieve the vaccination status of individuals infected with COIVD-19 and therefore relied on overall vaccination for each age group.

For peer review only - http://bmjopen.bmj.com/site/about/guidelines.xhtml

Introduction

Brazil has experienced the third highest number of confirmed COVID-19 deaths globally. Despite having a universal healthcare system that is experienced with managing public health emergencies(Domingues 2021), Brazil was unable to contain COVID-19(Li et al. 2021) and intensive care unit (ICU) beds consistently exceeded capacity in many states(Hallal and Victora 2021). This was complicated by the emergence of Gamma (P.1) and spread of Omicron (B.1.1.529) variants(Naveca et al. 2022). The COVID-19 vaccination campaign was launched across Brazil on January 17, 2021 and initially prioritised healthcare workers, individuals aged 90 years or older, indigenous communities, and institutionalised individuals. Four types of vaccines have been offered to date. However only Coronavac (Sinovac, China) and Covishield AZD1222 (Oxford- AstraZeneca, UK) vaccines were offered during the early phase of the campaign. Vaccine shortages and delays have caused unanticipated interruptions in the administration of doses after the first dose(Confederação Nacional de Municípios 2021).

During the campaign, dose allocation was determined by the total population of each state without adjusting for demographic differences. Given the increasing risk of SARS-CoV-2 infection and death with age(O'Driscoll et al. 2021), characterising vaccine uptake across age groups is critical for reducing inequalities and optimizing the distribution of limited vaccine doses. Little is known about how the vaccination programme impacts age-specific hospitalisations and COVID-19- related death rates(Hitchings et al. 2021). To optimise vaccine distribution, further investigation is needed to understand the age-specific reduction in the risk of severe COVID-19 and death, especially among those most vulnerable.

We investigated inequalities in vaccination coverage across Brazil in 2021 and assessed how potential driving factors, such as age, sex, race, and socioeconomic status, influenced coverage. We found several states delayed full vaccination for older populations to prioritise allocation for younger individuals. Among the population that was vaccinated, we estimated the proportion of individuals that did not take the second dose, showing large variation with income, race, sex, and across states. We also estimated the effect of

For peer review only - http://bmjopen.bmj.com/site/about/guidelines.xhtml

vaccination coverage on the risk of hospitalisation and death from COVID-19 and its associations with socioeconomic conditions. We further assessed the effects of vaccination coverage on death across age groups.

Methods

Data sources

Vaccination information

Patient level vaccination data notified between 17 January and 6 September, 2021 were retrieved from the Brazilian Ministry of Health's national immunization system, Sistema de Informação do Programa Nacional de Imunizações (SI-PNI). For every individual that received a dose, we retrieved their age, sex, municipality and state of residence, vaccination facility, dose number (e.g. first or second), and the vaccine type. We did not consider the booster dose campaign in our study, which started on September 15, 2021 with individuals of age 70 years and older.

Cases of severe acute respiratory illness

We retrieved data on hospitalisations and deaths in patients with severe acute respiratory illness (SARI) between 1 March, 2020 and 6 September, 2021, from the SRAG (Vigilância de Síndrome Respiratória Aguda Grave) database, which is curated by the Ministry of Health. Patient information on age, sex, and municipality of residence were included. All SARI cases and deaths were notified in the SRAG database regardless of hospitalisation. SARI can be caused by SARS-CoV-2 and is defined by the Ministry of Health as influenza-like illness plus one of the following: dyspnoea, persistent chest pain or hypoxia. We included both confirmed COVID-19 cases and SARI cases with unknown etiology, as those were likely related to COVID-19 but not lab-confirmed due to low rates of COVID-19 testing in Brazil and socioeconomic bias in testing(de Souza et al. 2020)(Li et al. 2021). All SARI cases confirmed to be caused by other respiratory viruses were excluded from the analysis. From

For peer review only

104 the remaining cases, we filtered for (1) all hospitalisations and, (2) all deaths (e.g., hospitalised deaths and other).

Socioeconomic conditions and population size

Measurements of socioeconomic indicators at the municipality level were retrieved from the latest population census (2010) compiled by the Brazilian Institute of Geography and Statistics(Censo Demográfico IBGE 2020). We selected indicators based on their relevance to the social determinants of health, such as household income per capita, proportion of residents with only a primary education or lower, and unemployment rates. Projected distributions of age and sex for the population in 2020 were obtained for each municipality and state(de Araújo Freire, Gonzaga, and Gomes 2020).

Data analysis

Age-group specific vaccination coverage

We estimated the proportion of the population vaccinated with at least one dose using the SI-PNI data and the projected population in each municipality as a whole, and for each age group within that municipality: under 20, 20–29, ... , 70–79, and 80 years and above(de Araújo Freire, Gonzaga, and Gomes 2020). We used the population estimates to account for the excess deaths caused by COVID-19.

To compare the levels of first dose coverage between older and younger populations, we defined a *campaign optimality index* (COI). The COI for age group A at threshold T is denoted as $\eta(A,T)$. The COI measures the proportion of the subsequent (older) age group at the time when the age group A first reached its threshold coverage of T. To compute the optimality index for threshold T and age group $A = [a_{\min}, a_{\max}]$ (which includes individuals aged between $a_{\min}$ and $a_{\max}$, we calculated the date when the coverage in that age group reached the threshold T and then defined $\eta(A,T)$ as the coverage of the subsequent age group $A_{\text{older}} = [a_{\min} + 10, a_{\max} + 10]$.

If vaccination was only offered to an age group A when everyone in the subsequent age group was vaccinated, then the COI would be 1. Smaller values of the COI for a particular

For peer review only - http://bmjopen.bmj.com/site/about/guidelines.xhtml

age group indicates that fewer people in the older age group were vaccinated. In an ideal scenario where the vaccination campaign for younger groups only begins after older groups achieve high coverage, the coverage of the older group A_{older} should be close to 100% when the coverage of the younger group reaches T, in which case $\eta(A,T) = 1$. The campaign optimality index was computed for all age groups and using thresholds of 25%, 50% and 75%.

Population that missed their second dose

We estimated the proportion of the population that delayed the second dose, i.e. the proportion did not receive their second dose within the suggested timeframe (21–28 day interval for Coronavac, and 90 days for all other approved 2-dose COVID-19 vaccines). To estimate the proportion of the population that missed the second dose entirely, we determined (i) the proportion of all first dose vaccinated individuals that did not have a second dose recorded, and (ii) from this, selected only those for which the time interval between the first dose and the date of the last entry in the dataset (September 06, 2021) is higher than the suggested time interval between doses for the corresponding vaccine plus a tolerance of 30 days. In other words, this is the proportion of individuals that did not take the second dose and whose second dose is delayed by at least 30 days. Both proportions were estimated for each age, sex, race and municipality. We excluded the single-dose vaccine of Janssen-Cilag from this analysis.

Risk of COVID-19 death by age-group

We used logistic regression to estimate the probability that a SARI patient would die with COVID-19. Due to substantial amount of missing vaccination data (54% of all SARI patients with symptoms onset before September 6th, reaching as high as 87% before March 1st), and uncertainty about the reason the data was missing, we used age group and sex specific vaccination coverage of the individual's municipality of residence as a proxy. For each SARI patient, we used their age group, the socioeconomic conditions, and vaccination coverage of their municipality of residence. We also included a binary indicator for whether the SARI case was hospitalised before the start of the vaccination campaign for their age group. See the SI for further details on the model used.

For peer review only

For peer review only - http://bmjopen.bmj.com/site/about/guidelines.xhtml

Risk of COVID-19 hospitalisation by age group

We used quasi-Poisson regression (GLM with a log-link function) to model the number of SARI hospitalisations in each state adjusting for age group, whether the hospitalisations occurred before or after the start of the vaccination campaign (for each age group and state), and each state's average age group specific vaccination coverage and socioeconomic factors such as the level of unemployment, education, and per capita income. Given data availability, we used the aggregated values for socioeconomic status across each state as a proxy for the individual's values. See the SI for further details on the model used.

Age distribution of death by vaccination coverage

To assess the potential protective effect of vaccination coverage by age, we looked at the distribution of ages among COVID-19 deaths in the SARI database. We modelled the ages as a sample from a multinomial distribution with parameter $\theta = (\theta_1,\ldots,\theta_n)$ where θ_j is the probability of a death belonging to the jth age group. The posterior distribution of θ is a Dirichlet distribution with parameters $\alpha = (1 + D_1,\ldots,1 + D_n)$, where D_j is the number of deaths observed in age group j, assuming a non-informative Dirichlet prior distribution with parameters (1,…,1). Sampling from the posterior distribution provided the marginal credible intervals for the proportion of COVID-19 deaths in each age group at four points in the epidemic: Before January 17, January 17 to March 16, March 17 to May 16, and May 17 to September 16, 2021.

Results

Geographical inequalities in vaccination coverage

Between January 17 and September 6, 2021, 133.4 million people were recorded with a first dose, corresponding to 84.4% of the population above 18 years of age. We determined the proportion of the population vaccinated with at least one dose in each municipality and mapped the distribution of coverage across states (Figure 1A). Similar trends can be observed for the proportion of the population that is fully vaccinated (Figure S1). At the

For peer review only - http://bmjopen.bmj.com/site/about/guidelines.xhtml

national level, older age groups have higher vaccination coverage, which decreases with age (Figure 1B). A greater proportion of women were vaccinated than men; this difference is more evident among younger age groups below the age of 50 years. Figure 1C shows that there is a clear regional divide in vaccination coverage, with the South and Southeast states having the highest coverage, and the North and Northeast states having the lowest coverage, although there is a substantial amount of variability between the municipalities within each state.

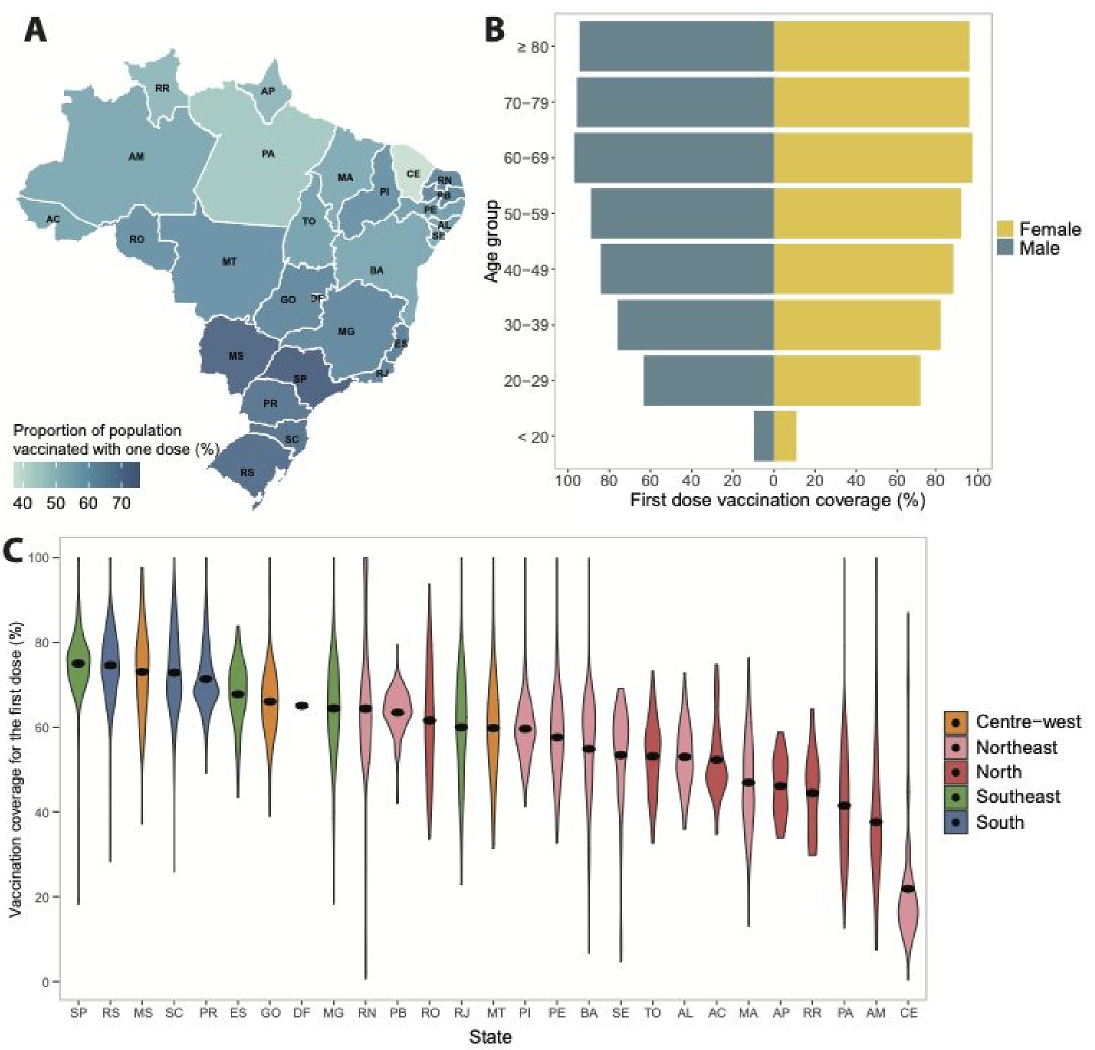

Figure 1. Inequalities in COVID-19 vaccination coverage across Brazil from January 17–September 6, 2021. (A) Percentage of population vaccinated with at least one dose across states as of September 6, 2021. (B) Distribution of vaccination uptake for the first dose

between men and women by age group. (C) Vaccination coverage across municipalities (black dot represents the mean coverage).

Premature advancement of the vaccination campaign towards younger groups

Figure 2A shows the proportion of the population vaccinated with a first dose in Brazil, by age group and sex over time. Due to uncertainty in total population size, coverage exceeds 100% for some groups. Similarly, coverages smaller than 100% may not imply the remaining population is not immunized. At the start of the campaign, both healthcare workers and older populations were prioritised for vaccination, hence delayed vaccination for younger groups. The vaccination coverage by sex shows the coverage for men remained consistently lower than women in all age groups over time.

We observed an unanticipated increase in coverage among younger age groups before older groups were fully vaccinated (Figure 2B). This pattern is accentuated in populations below 60 years of age. Using the Campaign Optimality Index (COI), we defined the coverage of older groups when the coverage of younger groups reached a threshold coverage of 25%, 50% and 75%. Larger values indicate a more staggered distribution indicating stronger prioritisation towards older age groups, while lower values indicate that age groups are being vaccinated together (i.e. weaker prioritisation). In Figure 2C, COI is higher for age groups above 50 years old, and drops substantially for younger groups. The difference between age groups is greatest for the threshold of 25% and decreases with higher thresholds, with COI being above 80% for all age groups for the threshold of 75%. Even though the vaccination campaign for younger groups started prematurely, the coverage of younger groups crossed the threshold of 75% when coverage for older groups was higher than 80%.

For peer review only - http://bmjopen.bmj.com/site/about/guidelines.xhtml

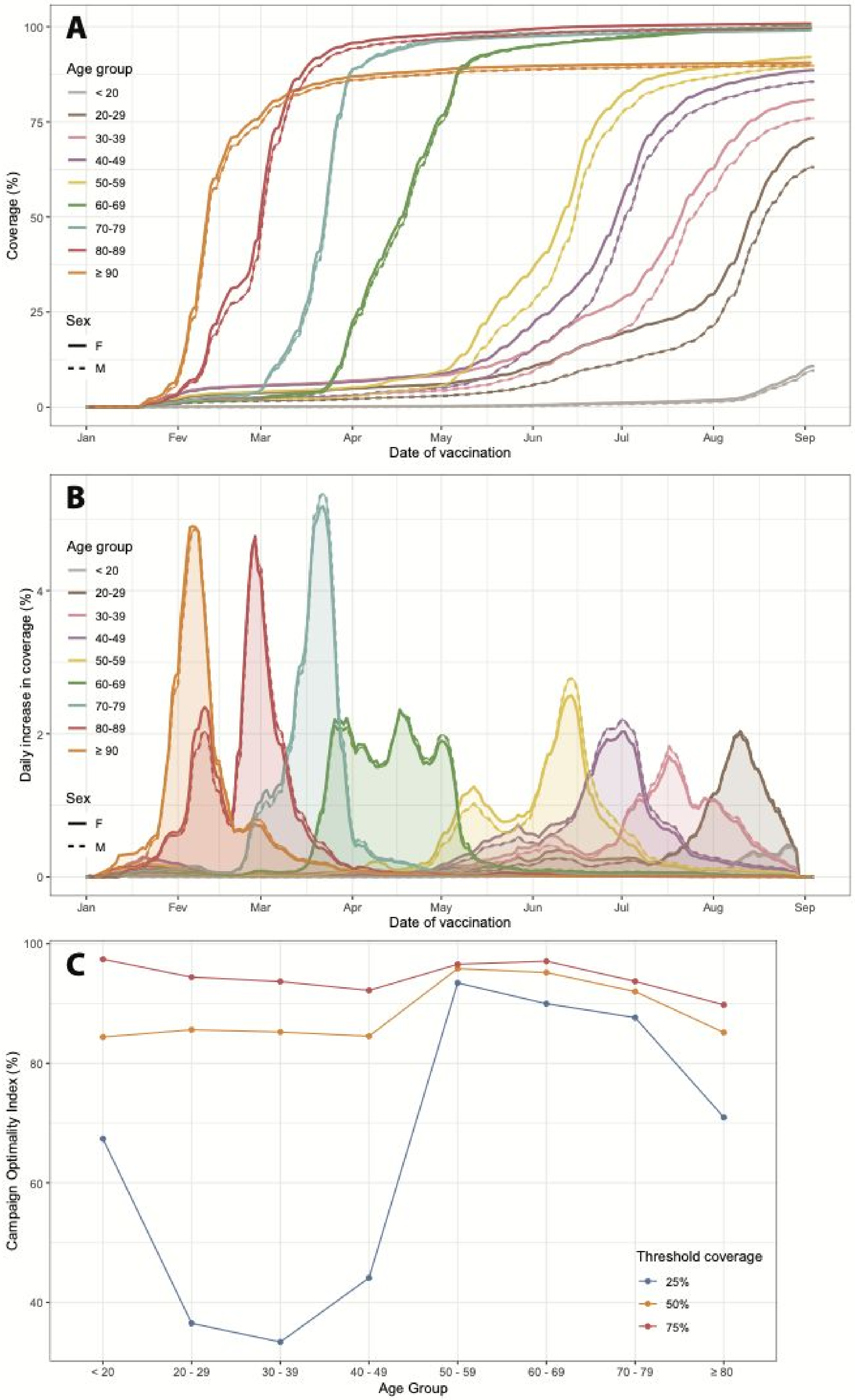
A
Age group
< 20
20-29
30-39
40-49
50-59
60-69
70-79
80-89
≥ 90
Sex
F
M
Coverage (%)
Date of vaccination
Jan
Fev
Mar
Apr
May
Jun
Jul
Aug
Sep
B
Daily increase in coverage (%)
C
Campaign Optimality Index (%)
Age Group
< 20
20 - 29
30 - 39
40 - 49
50 - 59
60 - 69
70 - 79
≥ 80
Threshold coverage
25%
50%
75%

220 1

Figure 2. COVID-19 vaccination uptake over time among age groups. (A) Vaccination coverage for the first dose over time. (B) Daily change in vaccination coverage by age group and sex. (C) Campaign optimality index (COI) by age group using threshold vaccination coverage of 25%, 50% and 75%. This index could not be calculated for the age group 20–29 using the threshold 75% because this group had not reached a coverage of 75% by September 6th 2021.

Probability of missing the second dose varies by sex, race, and income

By September 6, 2021, 11.9% and 10.2% of the individuals vaccinated with the first dose had their second dose, respectively, delayed and missed. Only 14.3% of individuals that did not take the second dose after 30 days past the recommended date ended up taking a delayed second dose. Among the individuals that were vaccinated with a first dose of Coronavac, 8.78% (95% CI 8.77%–8.80%) missed their second dose. This is low compared to the 12.27% of individuals that missed the second dose (95% CI 12.25%–12.29%) of other vaccines (Covishield and Pfizer), where the optimal interval between the first and second dose is 90 days.

The probability of missing the second dose by race and sex is shown in Figure 3A. Men were more likely to miss (Relative Risk(RR) 1.065, 95% CI 1.063–1.067) the second dose than women. Indigenous Brazilians were also 2.64 (95% CI 2.62–2.66) times more likely to miss the second dose than White Brazilians, followed by East Asian Brazilians (RR 1.68, 95% CI 1.68–1.69), Pardos (RR 1.65, 95% CI 1.65–1.65) and Black Brazilians (RR 1.51, 95% CI 1.50–1.51).

The probability of missing the second dose varies with income per capita (Figure 3B). Individuals in municipalities with a lower income per capita are more likely to miss their second dose. The median income per capita for municipalities with a 2 % probability of skipping the second dose is R$546,80 (IQR R$421,60–R$650,50). This decreases to R$270,40 (IQR R$212,60–R$420,80) when the probability of skipping is more than 10%. Figure 3C shows the variation in the probability of missing the second dose by state.

For peer review only - http://bmjopen.bmj.com/site/about/guidelines.xhtml

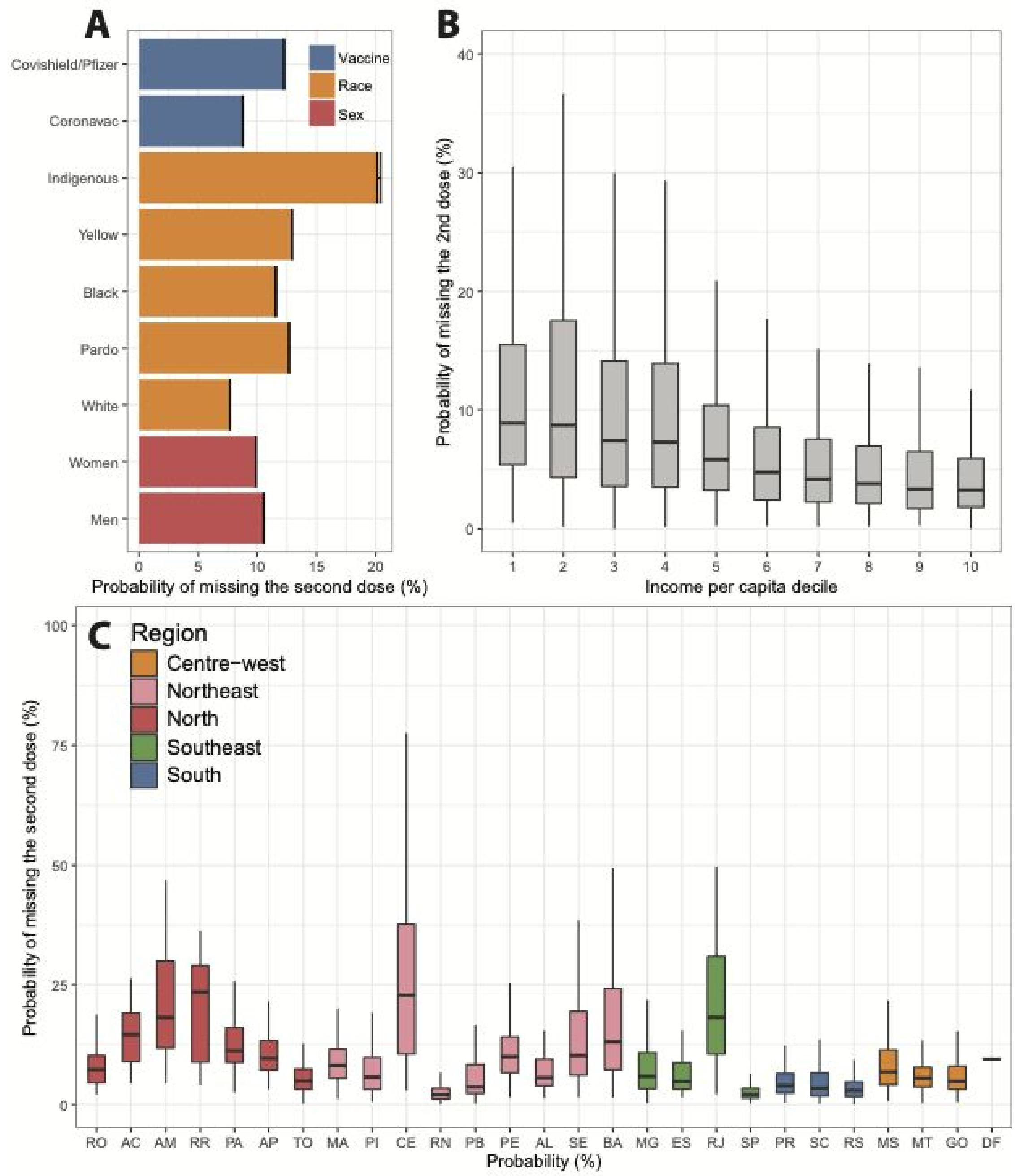

Figure 3. Probability of missing the second dose of the COVID-19 vaccine. (A) Probability of missing the second dose based on three factors: vaccine type, race and sex. (B) Probability of having a second dose missed calculated at municipality level in terms of the income per capita decile. (C) Probability of missing the second dose calculated for each municipality and grouped by state.

Vaccination decreases age-adjusted risk of death

Vaccination decreases the risk of COVID-19 death after adjusting for age group and socioeconomic conditions. Using 20 years of age and under as a baseline, the risk of COVID-19 death increased with age. For instance, the odds of death are at least ten times greater for someone above the age of 50 years (OR: 10.5–34.8, 95% CI: 10.2, 35.9) than an individual under 20 years of age. Patients living in municipalities with higher income per capita (OR: 0.919, 95% CI: 0.917, 0.920) and lower education attainment (OR: 0.934, 95% CI: 0.932, 0.936), respectively, had reduced risk of death. On the contrary, patients from municipalities with higher unemployment rates were slightly more likely to be at risk of death (OR: 1.009, 95% CI: 1.007, 1.010). When the vaccination campaign was accounted for, patients were at a lower risk of death after the start of the vaccination campaign (OR: 0.834, 95% CI: 0.832, 0.837); patients living in municipalities with higher vaccination coverage also had lower risk (OR: 0.996, 95% CI: 0.994, 0.997). Our sensitivity analysis at the individual level which includes only patients with a recorded vaccination status, showed that patients were at a lower risk of death after being vaccinated with at least one dose. While similar patterns for age groups were observed for hospitalisations, higher unemployment was associated with lower risk of hospitalisation. Detailed information on model outputs can be found in Table S1 and Table S2.

To verify the relationship between vaccination coverage and risk of death by age groups, we plotted the probability of a patient dying from COVID-19 being in a particular age group prior to January 17, 2021 and after the start of the vaccination campaign (including and after January 17, 2021) (Figure 4A). There is a higher probability that those dying before the start of the vaccination campaign were from an older age group. After vaccination began, those dying were more likely to be from age groups with ages below 60 years. This can be attributed to vaccination, where the proportion of vaccination is much higher among people in older age groups due to earlier uptake (Figure 4B). However, individuals under 20 years of age showed a reverse trend, where the proportion of deaths was higher prior to the vaccination campaign.

For peer review only

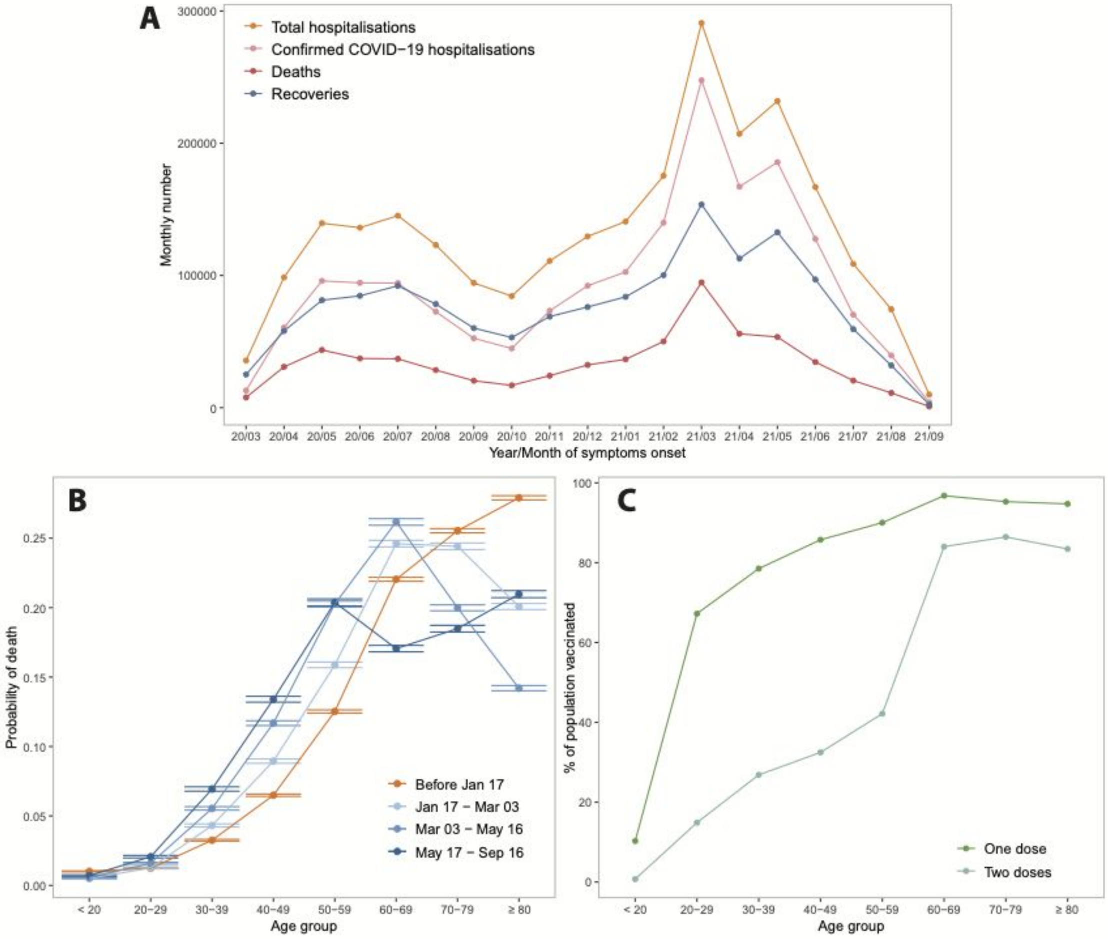

Figure 4. Risk of SARI death and vaccination coverage by age distribution. (A) Distribution of SARI hospitalisations over time (March 2020–September 2021). (B) The distribution of age among SARI-linked deaths at four points in time indicates that as the epidemic progressed SARI deaths were more likely to be younger individuals. (C) Proportion of population vaccinated with at least one dose by age group during study period.

Discussion

Inequalities exist in COVID-19 vaccination coverage, especially across states and different age, sex, and socioeconomic groups. Among patients diagnosed with COVID-19, the riskof hospitalisation and death are significantly greater among older individuals and those with lower socioeconomic status. Increasing vaccination reduces the risk of death, especially

For peer review only - http://bmjopen.bmj.com/site/about/guidelines.xhtml

among older age groups. Vaccine uptake is greater in areas where hospitalisation rates are high, suggesting better access to healthcare and improved reporting of infections.

Variation in coverage between population groups was mainly driven by the unequal distribution of vaccines across states. States from the North and Northeast regions, such as Amazonas, Amapá, and Ceará, were prioritised for vaccination in January 2021 due to high transmission rates of the P.1 (Gamma) variant, which was first detected in Manaus (the capital of Amazonas) in January 2021(Buss et al. 2021). Despite this, lower overall coverage for the first dose was observed in the North and Northeast regions of Brazil. These regions are economically less developed and have experienced scare hospital resources during the pandemic(Rocha et al. 2021). Delays in notification of vaccinations had an important impact on coverage between states. Even though we determined a 37.5% first-dose coverage for the northeastern state of Ceará, official statistics obtained directly from the Brazilian state secretariats on September 6, 2021 found this was 59.2% (G1 Globo, 2022). The low COI obtained with a threshold of 25% for age groups younger than 50 shows the vaccination campaign started prematurely for these groups, which may have delayed the vaccination of older groups. It is worth noting that the proposed COI only considers age as a priority criterion and does not include other criteria such as comorbidities or employment (e.g. healthcare worker).

Inequalities in uptake are evident between the sexes. Women were more likely to have received both doses than men. This may be owing to the early stages of the vaccination campaign, when healthcare workers were prioritised: women make up a significant proportion of healthcare workers in Brazil, accounting for 62.5% of all vaccinated healthcare workers, which corresponds to 10.6% of all vaccinated individuals in our dataset. Moreover, women seek out healthcare more frequently than men. Based on a survey study with over 170,000 responses, men showed greater hesitancy towards COVID-19 vaccines, indicating the main reason being fear of severe clinical reactions(Moore et al. 2021). We also found a longer delay for taking the second dose in municipalities with lower income per capita, which is aligned with previous findings that low incomes were associated with higher rates of vaccine hesitancy during the pandemic(Moore et al. 2021). This has implications for death risk, as our findings show that SARI patients from poor

For peer review only - http://bmjopen.bmj.com/site/about/guidelines.xhtml

municipalities are more likely to die from COVID-19 than those from wealthier municipalities.

Vaccination reduces the risk of death. The positive association between vaccination and hospitalisation appears to be driven by greater accessibility of both in wealthier areas. To further investigate whether vaccination can prevent COVID-19 related deaths for each age group, we considered the age distribution of those that died, across multiple periods of time. We observed a reduction in the proportion of mortality in older age groups once vaccination became available, since it was the elderly that were prioritised for vaccination. This trend was the least evident with young people under the age of 20. This was likely owing to the delayed vaccination of young people until July 2021. Due to a lack of complete population-level data, it may be premature to surmise the true protective effects of vaccination coverage on younger age groups. While the overall risk of death is lower for young people, COVID-19 still poses a threat, regardless of pre-existing medical conditions(Cunningham et al. 2021)(Taylor 2021).

Our estimates of vaccination coverage by state and age-group are limited by the accuracy of the projected population estimates. We were unable to retrieve the vaccination status of individual SARI patients and thus had to rely on overall vaccination coverage for each age group; this may have introduced discrepancies in the modelled relationship between vaccination coverage and the risk of death. Furthermore, human error affects the accuracy of vaccination records (e.g. date of vaccination could be replaced by the date when the information was entered into the system). Moreover, the lack of healthcare resources in some areas (e.g. Ceará) may have led to lower levels of case notification and notification delay. Self-identification of race, may be prone to bias, with wealthier Black Brazilian more inclined to self-identify and contribute race information than poorer Black Brazilians(Telles and Paschel 2014).

Despite some vaccine hesitancy among certain demographic groups, the overall intention to be vaccinated against COVID-19 is high(Gramacho and Turgeon 2021)(Moore et al. 2021). Vaccine hesitancy is a complex issue; a survey from late 2020 found that when certain countries of origin for the vaccine were mentioned (e.g. China and Brazil), the

For peer review only - http://bmjopen.bmj.com/site/about/guidelines.xhtml

willingness to vaccinate among Brazilians decreased. However, our study shows that individuals that were vaccinated with the first dose of Coronavac were less likely to skip the second dose when compared with individuals that were vaccinated with other vaccines, such Covishield or Pfizer. This may be partially attributed to the fact that the optimal interval between the first and second doses for most vaccines is 90 days, while the interval for Coronavac is only 21 to 28 days(Ministério da Saúde 2021). The disparities in vaccination coverage may have amplified the inequalities in risk of death during the epidemic in Brazil, as the Gamma variant is associated with an increased infection fatality rate(Prete et al. 2022).

The third booster dose has been controversial as parts of the population were still awaiting their second dose. Vaccination has a protective effect on populations(Rossman et al. 2021) but the details of how this is communicated to the general public are complex(Rossman et al. 2021). Our findings on inequalities in coverage and on demographic groups that are more willing to miss the second dose should be considered when designing media campaigns and interventions to increase vaccine access and uptake.

Funding

SLL is supported by the Oxford Martin Programme on Pandemic Genomics and the Canadian Social Sciences and Humanities Research Council (SSHRC) Doctoral Fellowship. CAPJ was supported by FAPESP (2019/21858-0 and 2022/15985-1). AEZ is supported by the Oxford Martin School Programme on Pandemic Genomics. CAPJ and VHN were supported by Coordenação de Aperfeicoamento de Pessoal de Nível Superior – Brasil (CAPES) – Finance Code 001. VHN is supported by the Brazilian National Council for Scientific and Technological Development (CNPq: 304714/2018-6). ECS is supported by a Medical Research Council-S˜ao Paulo Research Foundation (FAPESP) CADDE partnership award (MR/S0195/1 and FAPESP 18/14389-0) (http://caddecentre.org/).

For peer review only

For peer review only - http://bmjopen.bmj.com/site/about/guidelines.xhtml

References

Buss, Lewis F., Carlos A. Prete, Claudia M. M. Abrahim, Alfredo Mendrone, Tassila Salomon, Cesar de Almeida-Neto, Rafael F. O. França, et al. 2021. "Three-Quarters Attack Rate of SARS-CoV-2 in the Brazilian Amazon During a Largely Unmitigated Epidemic." *Science* 371 (6526): 288–92. https://doi.org/10.1126/science.abe9728.

Censo Demográfico IBGE. 2020. "Censo Demográfico." Available at https://www.ibge.gov.br/estatisticas/sociais/populacao/9662-censo-demografico-2010.html?=&t=o-que-e (2020/11/20).

Confederação Nacional de Municípios. 2021. "Pesquisa CNM — Covid-19." Available at https://www.cnm.org.br/cms/biblioteca/Pesquisa-CNM-Covid-19_Ed28.pdf.

Cunningham, Jonathan W., Muthiah Vaduganathan, Brian L. Claggett, Karola S. Jering, Ankeet S. Bhatt, Ning Rosenthal, and Scott D. Solomon. 2021. "Clinical Outcomes in Young US Adults Hospitalized With COVID-19." *JAMA Internal Medicine* 181 (3): 379–81. https://doi.org/10.1001/jamainternmed.2020.5313.

de Araújo Freire, Flávio Henrique Miranda, Marcos Roberto Gonzaga, and Marília Miranda Forte Gomes. 2020. "Projeções Populacionais Por Sexo E Idade Para Pequenas áreas No Brasil." *Revista Latinoamericana de Población* 14 (26): 124–49. https://doi.org/10.31406/relap2020.v14.i1.n26.6.

de Souza, William Marciel, Lewis Fletcher Buss, Darlan da Silva Candido, Jean-Paul Carrera, Sabrina Li, Alexander E. Zarebski, Rafael Henrique Moraes Pereira, et al. 2020. "Epidemiological and Clinical Characteristics of the COVID-19 Epidemic in Brazil." *Nature Human Behaviour* 4 (8): 856–65. https://doi.org/10.1038/s41562-020-0928-4.

Domingues, Carla Magda Allan Santos. 2021. "Desafios Para a Realização Da Campanha de Vacinação Contra a COVID-19 No Brasil." *Cadernos de Saúde Pública* 37 (1).

Gramacho, Wladimir G., and Mathieu Turgeon. 2021. "When Politics Collides with Public Health: COVID-19 Vaccine Country of Origin and Vaccination Acceptance in Brazil." *Vaccine* 39 (19): 2608–12. https://doi.org/10.1016/j.vaccine.2021.03.080.

For peer review only

For peer review only - http://bmjopen.bmj.com/site/about/guidelines.xhtml

Hallal, Pedro C., and Cesar G. Victora. 2021. "Overcoming Brazil's Monumental COVID-19 Failure: An Urgent Call to Action." *Nature Medicine* 27 (6): 933–33. https://doi.org/10.1038/s41591-021-01353-2.

Hitchings, Matt D. T., Otavio T. Ranzani, Mario Sergio Scaramuzzini Torres, Silvano Barbosa de Oliveira, Maria Almiron, Rodrigo Said, Ryan Borg, et al. 2021. "Effectiveness of CoronaVac Among Healthcare Workers in the Setting of High SARS-CoV-2 Gamma Variant Transmission in Manaus, Brazil: A Test-Negative Case-Control Study." *The Lancet Regional Health - Americas* 1: 100025. https://doi.org/10.1016/j.lana.2021.100025.

Li, Sabrina L, Rafael H M Pereira, Carlos A Prete Jr, Alexander E Zarebski, Lucas Emanuel, Pedro J H Alves, Pedro S Peixoto, et al. 2021. "Higher Risk of Death from COVID-19 in Low-Income and Non-White Populations of São Paulo, Brazil." *BMJ Global Health* 6 (4). https://doi.org/10.1136/bmjgh-2021-004959.

Ministério da Saúde. 2021. "Plano Nacional de Operatcionalização Da Vacinação Contra a COVID-19." Brasília - DF.

Moore, Daniella Campelo Batalha Cox, Marcio Fernandes Nehab, Karla Gonçalves Camacho, Adriana Teixeira Reis, Maria de Fátima Junqueira-Marinho, Dimitri Marques Abramov, Zina Maria Almeida de Azevedo, et al. 2021. "Low COVID-19 Vaccine Hesitancy in Brazil." *Vaccine* 39 (42): 6262–8. https://doi.org/10.1016/j.vaccine.2021.09.013.

Naveca, F. G., V. Nascimento, V. Souza, F. Nascimento, M. Mejia, M. J. Brandão, A. V. da Silva, et al. 2022. "The Dissemination of the Omicron Variant in the Highly Seroprevalent Amazonas State, Brazil, Is Associated with a Rapid Upsurge of SARS-CoV-2 Cases." *Virological*.

O'Driscoll, Megan, Gabriel Ribeiro Dos Santos, Lin Wang, Derek A. T. Cummings, Andrew S. Azman, Juliette Paireau, Arnaud Fontanet, Simon Cauchemez, and Henrik Salje. 2021. "Age-Specific Mortality and Immunity Patterns of SARS-CoV-2." *Nature* 590 (7844): 140–45. https://doi.org/10.1038/s41586-020-2918-0.

For peer review only - http://bmjopen.bmj.com/site/about/guidelines.xhtml

Prete, Carlos A, Jr, Lewis F Buss, Charles Whittaker, Tassila Salomon, Marcio K Oikawa, Rafael H M Pereira, Isabel C G Moura, et al. 2022. “SARS-CoV-2 Antibody Dynamics in Blood Donors and COVID-19 Epidemiology in Eight Brazilian State Capitals: A Serial Cross-Sectional Study.” *eLife* 11.

Rocha, Rudi, Rifat Atun, Adriano Massuda, Beatriz Rache, Paula Spinola, Letícia Nunes, Miguel Lago, and Marcia C Castro. 2021. “Effect of Socioeconomic Inequalities and Vulnerabilities on Health-System Preparedness and Response to COVID-19 in Brazil: A Comprehensive Analysis.” *The Lancet Global Health* 9 (6): e782–e792.

Rossman, Hagai, Smadar Shilo, Tomer Meir, Malka Gorfine, Uri Shalit, and Eran Segal. 2021. “COVID-19 Dynamics After a National Immunization Program in Israel.” *Nature Medicine* 27 (6): 1055–61. https://doi.org/10.1038/s41591-021-01337-2.

Taylor, Luke. 2021. “Covid-19: Brazil’s Spiralling Crisis Is Increasingly Affecting Young People.” *BMJ* 373. https://doi.org/10.1136/bmj.n879.

Telles, Edward, and Tianna Paschel. 2014. “Who Is Black, White, or Mixed Race? How Skin Color, Status, and Nation Shape Racial Classification in Latin America.” *American Journal of Sociology* 120 (3): 864–907. https://doi.org/10.1086/679252.

For peer review only

For peer review only - http://bmjopen.bmj.com/site/about/guidelines.xhtml

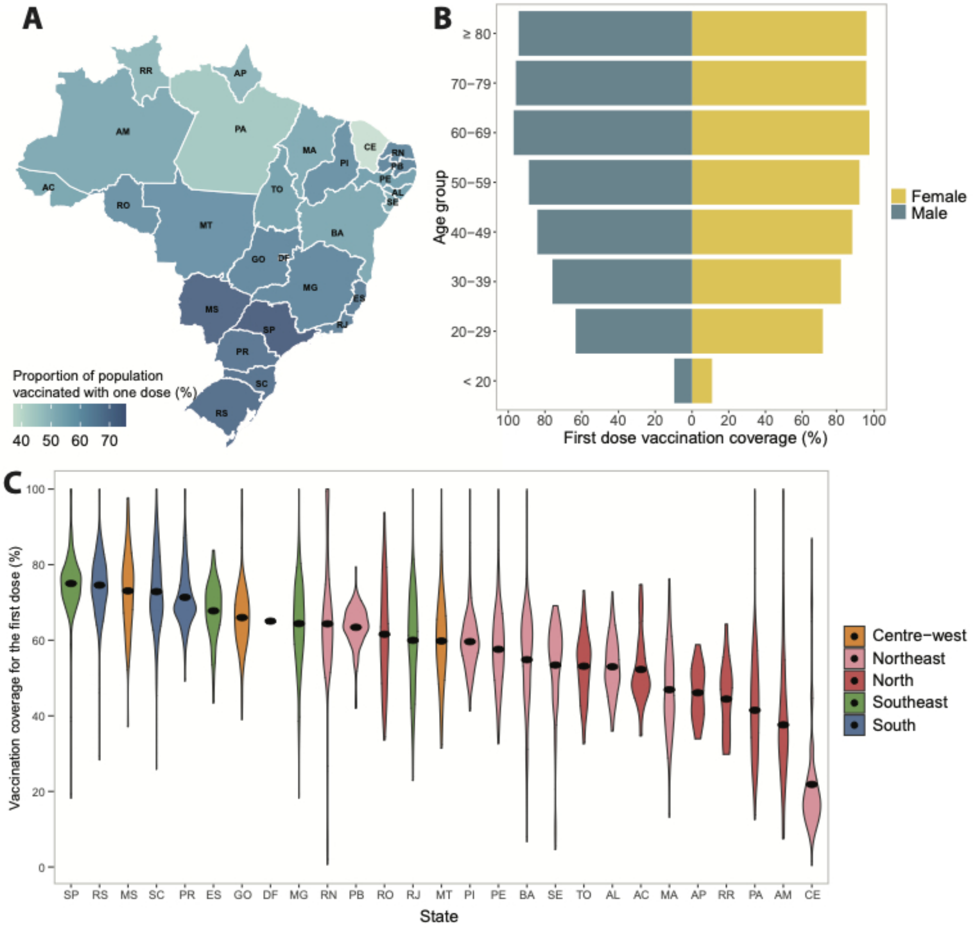

Fig 1

274x259mm (72 x 72 DPI)

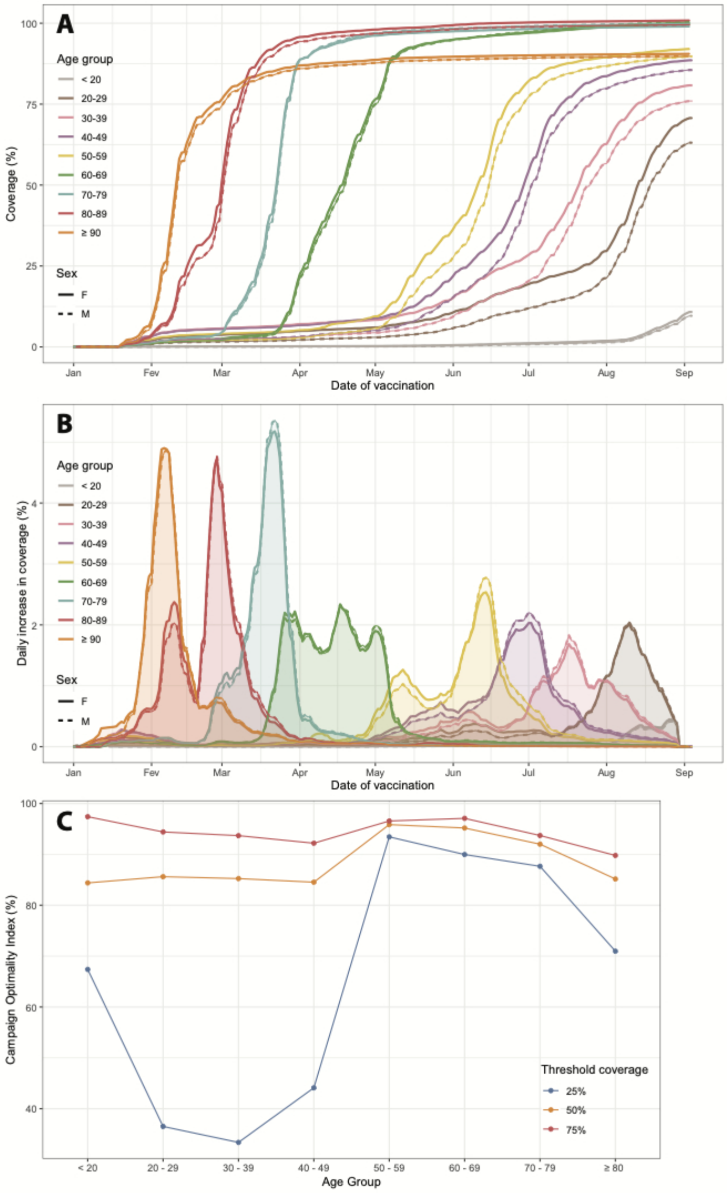

Fig 2

234x381mm (72 x 72 DPI)

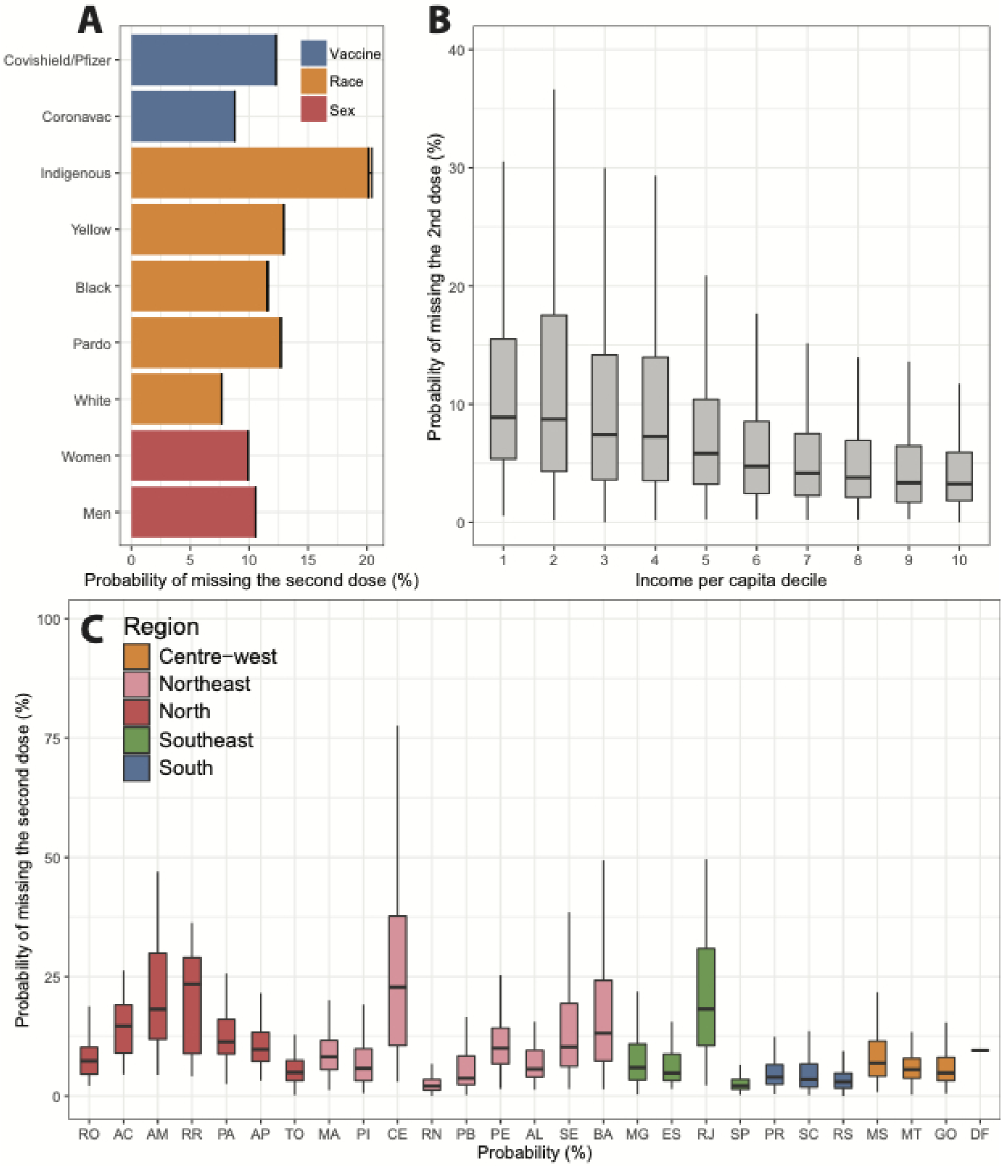

Fig 3

219x257mm (72 x 72 DPI)

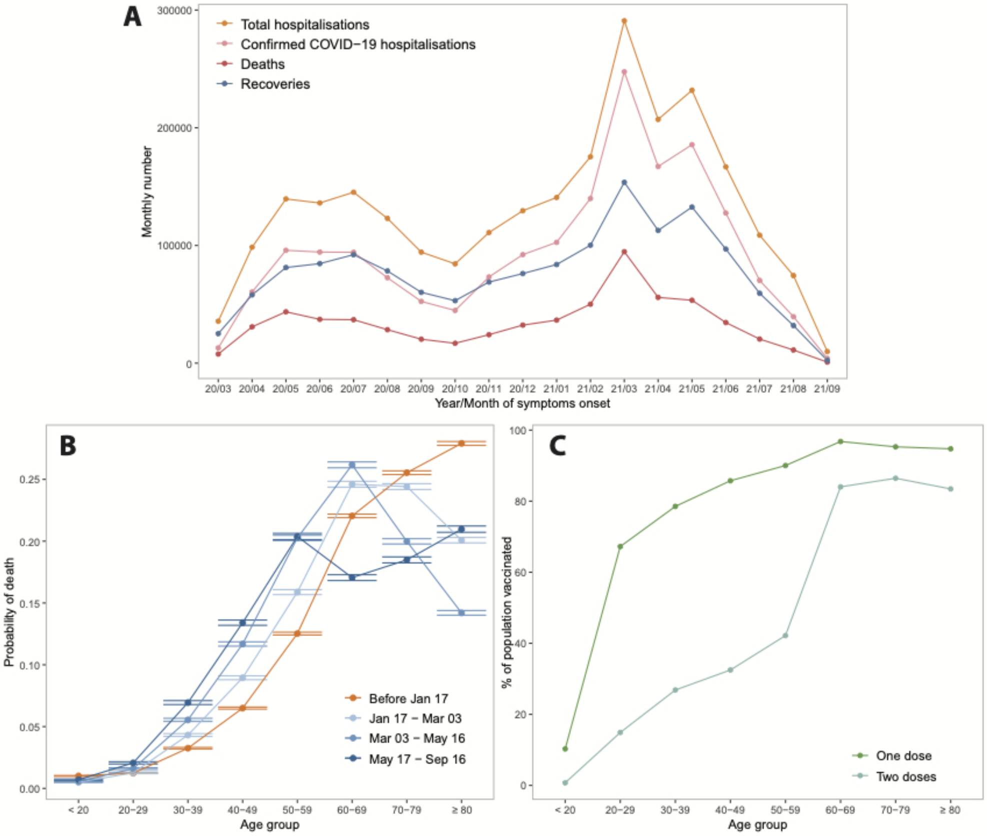

Fig 4

302x255mm (72 x 72 DPI)

Supplementary Information: The Brazilian COVID-19 vaccination campaign: A modelling analysis of socio-demographic factors on uptake

For peer review only

Risk of COVID-19 death by age-group

The equation describing the log-odds of death among SARI patients is expressed as

$$\begin{aligned}\operatorname{logit}(p_i) = \beta_0 &+ \beta_1 \cdot (\text{Age group of } i)\\ &+ \beta_2 \cdot (\text{Vaccination coverage in age group of } i)\\ &+ \beta_3 \cdot (\text{Campaign status for } i)\\ &+ \beta_4 \cdot (\text{Unemployment for age group of } i)\\ &+ \beta_5 \cdot (\text{Low education attainment for age group of } i) + \epsilon_i,\end{aligned} \tag{1}$$

where $p_i = \mathbb{P}(Y_i = 1)$ is the log-odds of death where Y_i is an indicator variable for whether the ith SARI patient's outcome: dead = 1, and alive = 0. The $beta_j$ are the coefficients of the model and ϵ_i is the error term. For reference, the logit function is defined as $\operatorname{logit}(p) = \log(p/(1-p))$.

As a form of sensitivity analysis, we fit the model at the individual level by including only patients that had a recorded vaccination status (e.g. vaccinated with at least one dose, or no vaccination). Here we included vaccination status as a binary indicator in the model to replace the age-group specific vaccination coverage. While this avoids the potential for an ecological fallacy (i.e. assuming population level vaccination levels are indicative of the individuals represented in the data), we cannot rule out the possibility that data may not be missing at random, which may introduce biases in the estimates.

The resulting parameter estimates from this model and their confidence intervals are given in Table S1.

Risk of COVID-19 hospitalisation by age group

The equation describing the expected number of hospitalisations in this model is

Variable	Coefficient (95% CI)	Significance
Intercept	-3.60 (-3.63, -3.57)	*
Age group#: 20-29 years old	1.17 (1.14, 1.21)	*
Age group: 30-39 years old	1.51 (1.14, 1.21)	*
Age group: 40-49 years old	1.91 (1.88, 1.94)	*
Age group: 50-59 years old	2.35 (2.32, 2.37)	*
Age group: 60-69 years old	2.87 (2.84, 2.90)	*
Age group: 70-79 years old	3.21 (3.18, 3.24)	*
Age group: 80 years and older	3.55 (3.52, 3.58)	*
Age-group specific coverage	-0.00414 (-0.00568, -0.00261)	*
Age-group specific campaign (yes)	-0.182 (-0.186, -0.178)	*
Unemployed	0.0241 (0.0221, 0.0261)	*
Primary education or lower	-0.0737 (-0.0758, -0.0716)	*
Income per capita	-0.0579 (-0.0602,-0.0556)	*

Baseline age group for comparison is under 20 years old
* statistically significant p-value < 0.05

Table S1: Logistic regression model analysis — death risk of SARI patient given age group, socioeconomic status, age group-specific vaccination coverage, and indication of age-specific vaccination campaign.

$$\begin{aligned}\log(\mathbb{E}[Y_i]) = \beta_0 &+ \beta_1 \cdot (\text{Age group of } i) \\ &+ \beta_2 \cdot (\text{Vaccination coverage in age group of } i) \\ &+ \beta_3 \cdot (\text{Campaign status for } i) \\ &+ \beta_4 \cdot (\text{Unemployment for age group of } i) \\ &+ \beta_5 \cdot (\text{Low education attainment for age group of } i) \\ &+ \beta_6 \cdot (\text{Income percapita for age group of } i) + \epsilon_i,\end{aligned} \tag{2}$$

with the variance $\text{Var}(Y_i) = \phi(Y_i)$ where ϕ is the overdispersion parameter and ϵ_i is the error term.

The resulting parameter estimates from this model and their confidence intervals are given in Table S2.

For peer review only

Variable	Coefficient (95% CI)	Significance
Intercept	-6.67 (-7.45, -5.89)	*
Age group#: 20-29 years old	-0.0427(-0.161, -0.246)	
Age group: 30-39 years old	0.884 (0.721, 1.05)	*
Age group: 40-49 years old	1.39 (1.24, 1.54)	*
Age group: 50-59 years old	1.84 (1.69, 1.98)	*
Age group: 60-69 years old	2.19 (2.04, 2.34)	*
Age group: 70-79 years old	2.62 (2.47, 2.77)	*
Age group 80 years and older	3.16 (3.02, 3.30)	*
Age-group specific coverage	0.00875(0.000612, 0.0169)	*
Age-group specific campaign (yes)	0.0533 (-0.000682, 0.115)	
Unemployed	-3.98 (-5.65, -2.31)	*
Primary education or lower	1.94 (0.502, 3.37)	*
Income per capita	-0.000363 (0.0000902,0.0000982)	*

Baseline age group for comparison is under 20 years old
* statistically significant p-value < 0.05

Table S2: Quasi-Poisson regression model analysis — hospitalisation risk given age group, socioeconomic status, age group-specific vaccination coverage, and indication of age-specific vaccination campaign

For peer

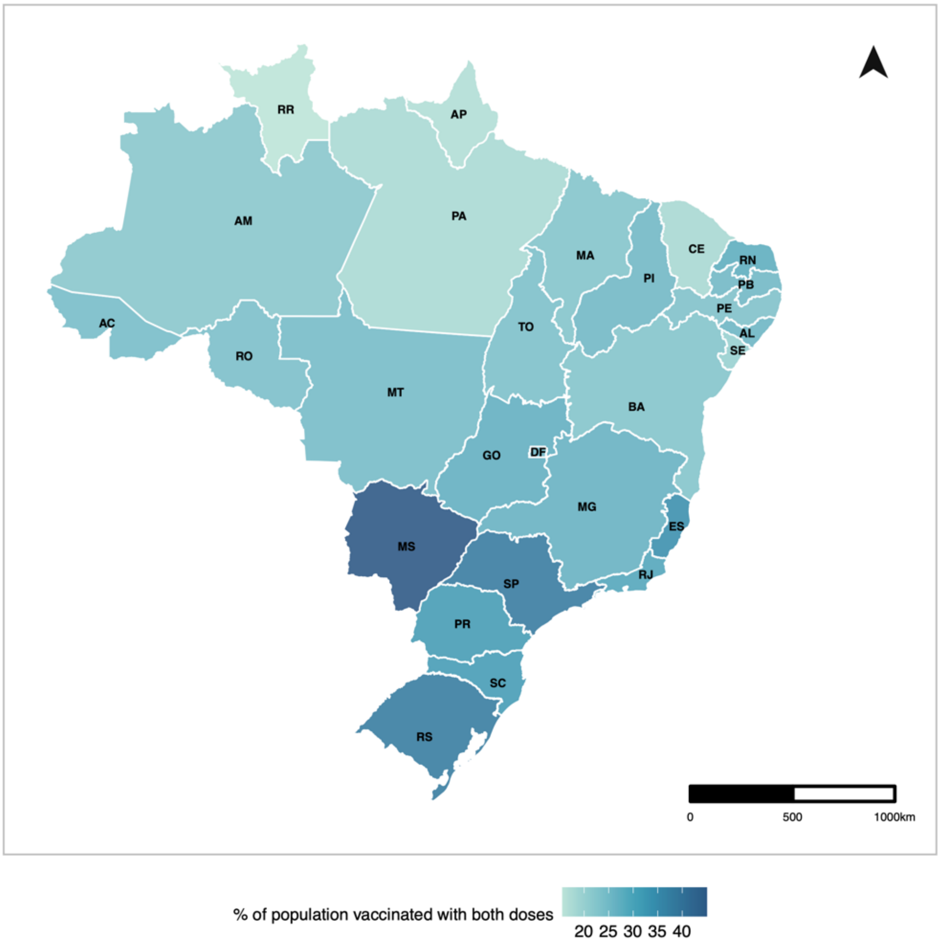

Figure S1: Proportion of population fully vaccinated.

Reporting checklist for cross sectional study.

Based on the STROBE cross sectional guidelines.

Instructions to authors

Complete this checklist by entering the page numbers from your manuscript where readers will find each of the items listed below.

Your article may not currently address all the items on the checklist. Please modify your text to include the missing information. If you are certain that an item does not apply, please write "n/a" and provide a short explanation.

Upload your completed checklist as an extra file when you submit to a journal.

In your methods section, say that you used the STROBE cross sectionalreporting guidelines, and cite them as:

von Elm E, Altman DG, Egger M, Pocock SJ, Gotzsche PC, Vandenbroucke JP. The Strengthening the Reporting of Observational Studies in Epidemiology (STROBE) Statement: guidelines for reporting observational studies.

		Reporting Item	Page Number
**Title and abstract**			
Title	#1a	Indicate the study's design with a commonly used term in the title or the abstract	1
Abstract	#1b	Provide in the abstract an informative and balanced summary of what was done and what was found	1
**Introduction**			
Background / rationale	#2	Explain the scientific background and rationale for the investigation being reported	2
Objectives	#3	State specific objectives, including any prespecified hypotheses	2
**Methods**			
Study design	#4	Present key elements of study design early in the paper	3-6
Setting	#5	Describe the setting, locations, and relevant dates, including periods of	3-6

For peer review only

		recruitment, exposure, follow-up, and data collection	
Eligibility criteria	#6a	Give the eligibility criteria, and the sources and methods of selection of participants.	3-6
	#7	Clearly define all outcomes, exposures, predictors, potential confounders, and effect modifiers. Give diagnostic criteria, if applicable	3-6
Data sources / measurement	#8	For each variable of interest give sources of data and details of methods of assessment (measurement). Describe comparability of assessment methods if there is more than one group. Give information separately for for exposed and unexposed groups if applicable.	3-6
Bias	#9	Describe any efforts to address potential sources of bias	3-6
Study size	#10	Explain how the study size was arrived at	3-6
Quantitative variables	#11	Explain how quantitative variables were handled in the analyses. If applicable, describe which groupings were chosen, and why	3-6
Statistical methods	#12a	Describe all statistical methods, including those used to control for confounding	3-6
Statistical methods	#12b	Describe any methods used to examine subgroups and interactions	3-6
Statistical methods	#12c	Explain how missing data were addressed	3-6
Statistical methods	#12d	If applicable, describe analytical methods taking account of sampling strategy	3-6
Statistical methods	#12e	Describe any sensitivity analyses	3-6
**Results**			
Participants	#13a	Report numbers of individuals at each stage of study—eg numbers potentially eligible, examined for eligibility, confirmed eligible, included in the study, completing follow-up, and analysed. Give information separately for for exposed and unexposed groups if applicable.	3-6
Participants	#13b	Give reasons for non-participation at each stage	3-6
Participants	#13c	Consider use of a flow diagram	3-6

For peer review only

Descriptive data	#14a	Give characteristics of study participants (eg demographic, clinical, social) and information on exposures and potential confounders. Give information separately for exposed and unexposed groups if applicable.	3-6
Descriptive data	#14b	Indicate number of participants with missing data for each variable of interest	6
Outcome data	#15	Report numbers of outcome events or summary measures. Give information separately for exposed and unexposed groups if applicable.	6-7
Main results	#16a	Give unadjusted estimates and, if applicable, confounder-adjusted estimates and their precision (eg, 95% confidence interval). Make clear which confounders were adjusted for and why they were included	6-13
Main results	#16b	Report category boundaries when continuous variables were categorized	6-13
Main results	#16c	If relevant, consider translating estimates of relative risk into absolute risk for a meaningful time period	6-13
Other analyses	#17	Report other analyses done—e.g., analyses of subgroups and interactions, and sensitivity analyses	6-13
**Discussion**			
Key results	#18	Summarise key results with reference to study objectives	13-14
Limitations	#19	Discuss limitations of the study, taking into account sources of potential bias or imprecision. Discuss both direction and magnitude of any potential bias.	15
Interpretation	#20	Give a cautious overall interpretation considering objectives, limitations, multiplicity of analyses, results from similar studies, and other relevant evidence.	13-16
Generalisability	#21	Discuss the generalisability (external validity) of the study results	16
**Other Information**			
Funding	#22	Give the source of funding and the role of the funders for the present study and, if applicable, for the original study on which the present article is based	16

The STROBE checklist is distributed under the terms of the Creative Commons Attribution License CC-BY.
This checklist was completed on 16. May 2023 using https://www.goodreports.org/, a tool made by the EQUATOR Network in collaboration with Penelope.ai

For peer review only

BMJ Open

The Brazilian COVID-19 vaccination campaign: A modelling analysis of socio-demographic factors on uptake

Journal:	*BMJ Open*
Manuscript ID	bmjopen-2023-076354.R1
Article Type:	Original research
Date Submitted by the Author:	02-Nov-2023
Complete List of Authors:	Li, Sabrina; University of Nottingham,<br>Prete Junior, Carlos Augusto; Universidade de São Paulo,<br>Zarebski, Alexander; Oxford University; The University of Melbourne<br>Santos, Andreza ; University of Oxford<br>Sabino, Ester; Universidade de São Paulo<br>Nascimento, Vitor; Universidade de Sao Paulo<br>Wu, Chieh-Hsi ; University of Southampton,<br>Messina, Jane; University of Oxford,
**Primary Subject Heading**:	Public health
Secondary Subject Heading:	Epidemiology, Global health, Infectious diseases
Keywords:	COVID-19, PUBLIC HEALTH, Health Equity, Vaccination

SCHOLARONE™
Manuscripts

I, the Submitting Author has the right to grant and does grant on behalf of all authors of the Work (as defined in the below author licence), an exclusive licence and/or a non-exclusive licence for contributions from authors who are: i) UK Crown employees; ii) where BMJ has agreed a CC-BY licence shall apply, and/or iii) in accordance with the terms applicable for US Federal Government officers or employees acting as part of their official duties; on a worldwide, perpetual, irrevocable, royalty-free basis to BMJ Publishing Group Ltd ("BMJ") its licensees and where the relevant Journal is co-owned by BMJ to the co-owners of the Journal, to publish the Work in this journal and any other BMJ products and to exploit all rights, as set out in our licence.

The Submitting Author accepts and understands that any supply made under these terms is made by BMJ to the Submitting Author unless you are acting as an employee on behalf of your employer or a postgraduate student of an affiliated institution which is paying any applicable article publishing charge ("APC") for Open Access articles. Where the Submitting Author wishes to make the Work available on an Open Access basis (and intends to pay the relevant APC), the terms of reuse of such Open Access shall be governed by a Creative Commons licence – details of these licences and which Creative Commons licence will apply to this Work are set out in our licence referred to above.

Other than as permitted in any relevant BMJ Author's Self Archiving Policies, I confirm this Work has not been accepted for publication elsewhere, is not being considered for publication elsewhere and does not duplicate material already published. I confirm all authors consent to publication of this Work and authorise the granting of this licence.

For peer review only

The Brazilian COVID-19 vaccination campaign: A modelling analysis of socio-demographic factors on uptake

Abstract

Objective: Dose shortages delayed access to COVID-19 vaccination. We aim to characterize inequality in two-dose vaccination by sociodemographic group across Brazil.

Design: This is a cross-sectional study.

Setting: We utilise data retrieved from the Brazilian Ministry of Health databases published between 17 January – 6 September 2021.

Methods: We assessed geographical inequalities in full vaccination coverage and dose by age, sex, race, and socioeconomic status. We developed a campaign optimality index to characterize inequality in vaccination access due to premature vaccination towards younger populations before older and vulnerable populations were fully vaccinated. Generalized linear regression was used to investigate the risk of death and hospitalisation by age group, socioeconomic status, and vaccination coverage.

Results: Vaccination coverage is higher in the wealthier South and Southeast. Men, people of colour, and low-income groups were more likely to be only partially vaccinated due to missing or delaying a second dose. Vaccination started prematurely for age groups under 50 years which may have hindered uptake in older age groups. Vaccination coverage was associated with a lower risk of death, especially in older age groups (OR: 9.7–29.0, 95% CI: 9.4, 29.9). Risk of hospitalisation was greater in areas with higher vaccination rates due to higher access to care and reporting.

Conclusions: Vaccination inequality persists between states, age and demographic groups despite increasing uptake. The association between hospitalisation rates and vaccination is attributed to preferential delivery to areas of greater transmission and access to healthcare.

For peer review only

Strengths and Limitations

- A key strength of this study is that data were collected from the Brazilian Ministry of Health's national immunization system which included information on every individual that participated in the vaccination programme.
- We combined spatial data on vaccination, cases of severe acute respiratory illnesses and confirmed COVID-19 cases (due to low rates of COVID-19 testing in Brazil), socioeconomic conditions, and recent population estimates from 2020 to account for excess COVID-19 deaths.
- We built a spatially explicit model to robustly infer vaccination uptake and health risk across Brazil.
- We developed a vaccination Campaign Optimality Index (COI) which describes how vaccination was rolled out, estimating the extent to which older groups were and were not prioritized during the campaign.
- Our results are limited by being unable to retrieve the vaccination status of individuals infected with COIVD-19 and therefore relied on overall vaccination for each age group and municipality.

For peer review only

Introduction

During the COVID-19 pandemic, Brazil experienced the third highest number of confirmed COVID-19 deaths globally. Despite having a universal healthcare system that is experienced with managing public health emergencies [1], Brazil was unable to contain COVID-19 [2]and intensive care unit (ICU) beds consistently exceeded capacity in many states [3]. This was complicated by the emergence of the Gamma (P.1) and Omicron (B.1.1.529) variants [4].The COVID-19 vaccination campaign was launched across Brazil on January 17, 2021 and initially prioritised healthcare workers, individuals aged 90 years or older, indigenous communities, and institutionalised individuals. Four types of vaccines have been offered during the pandemic. However only Coronavac (Sinovac, China) and Covishield AZD1222 (Oxford-

AstraZeneca, UK) vaccines were offered during the early phase of the campaign. Vaccine shortages and delays have caused unanticipated interruptions in the administration of doses after the first dose [5].

Equitable and ethical administration of vaccines should be strived for; however, this remains an ongoing challenge [6,7]. During the campaign, dose allocation was determined by the total population of each state without adjusting for demographic differences. While older age is associated with varying risk of SARS-CoV-2 infection [8], it is associated with increasing risk of hospitalisation and death [9,10]. Thus, understanding vaccine uptake across age groups is critical for reducing mortality and health inequalities, and optimizing the distribution of limited vaccine doses. More importantly, little is known about how the vaccination programme impacts age-specific hospitalisations and COVID-19- related death rates [11]. To optimise vaccine distribution, further investigation is needed to understand the age-specific reduction in the risk of severe COVID-19 and death, especially among those most vulnerable.

We investigated inequalities in vaccination coverage across Brazil in 2021 and assessed how factors such as age, sex, race, and socioeconomic status, influenced uptake. We found several states delayed full vaccination for older populations to prioritise allocation for younger individuals. Among the population that was vaccinated, we estimated the proportion of individuals that did not take the second dose, finding large variation with income, race, sex, and across states. We also estimated the effect of vaccination on the risk of hospitalisation and death from COVID-19 and its associations with socioeconomic conditions. We further assessed the effects of vaccination coverage on death across age groups.

Methods

Patient and Public Involvement

None.

For peer review only - http://bmjopen.bmj.com/site/about/guidelines.xhtml

3

Data sources

Vaccination information

Patient level vaccination data notified between 17 January and 6 September, 2021 were retrieved from the Brazilian Ministry of Health's national immunization system, Sistema de Informação do Programa Nacional de Imunizações (SI-PNI) (https://opendatasus.saude.gov.br/dataset/covid-19-vacinacao). For every individual that received a dose, we retrieved their age, sex, municipality and state of residence, vaccination facility, dose number (e.g. first or second), and the vaccine type. We did not consider the booster dose campaign in our study, which started on September 15, 2021.

Cases of severe acute respiratory illness

We retrieved data on hospitalisations and deaths in patients with severe acute respiratory illness (SARI) between 1 March, 2020 and 6 September, 2021, from the SIVEP-Gripe database, curated by the Ministry of Health of Brazil. Patient information on age, sex, and municipality of residence were included. All SARI cases and deaths were notified in the SRAG database regardless of hospitalisation. SARI can be caused by SARS-CoV-2 and is defined by the Ministry of Health as influenza-like illness plus one of the following: dyspnea, persistent chest pain or hypoxia. We selected SARI hospitalisations and deaths, disregarding non-hospitalised individuals that did not die. All SARI cases confirmed to be caused by other respiratory viruses were excluded from the analysis. We included both confirmed COVID-19 cases and SARI cases with unknown etiology, as those were likely related to COVID-19 but not lab-confirmed [2,12]. This might be due to two reasons: the low rates of COVID-19 testing in Brazil, especially in poorer areas as shown by de Souza et al. [12]; and high proportion of false negative tests that were likely applied after the time window where SARS-CoV-2 is detectable by PCR. From all SARI cases with unknown or missing etiology with symptom onset during the study period, 24.9% did not have a PCR test result reported in SIPEP-Gripe, and most of the remaining 75.1% had likely a false negative test result.

For peer review only - http://bmjopen.bmj.com/site/about/guidelines.xhtml

Socioeconomic conditions and population size

Measurements of socioeconomic indicators at the municipality level were retrieved from the latest population census (2010) compiled by the Brazilian Institute of Geography and Statistics [13]. We selected indicators based on their relevance to the social determinants of health, such as household income per capita, proportion of residents with only a primary education or lower, and unemployment rates. Projected distributions of age and sex for the population in 2020 were obtained for each municipality and state [14]. Incomes were converted from Reais 2010 to US$ Purchase Power Parity (PPP) 2010 using the conversion rate of 1.388, available at https://data.oecd.org/conversion/purchasing-power-parities-ppp.htm.

Data analysis

Age-group specific vaccination coverage

We estimated the proportion of the population vaccinated with at least one dose using the SI-PNI data and the projected population in each municipality, and for each age group within that municipality: under 20, 20–29, ..., 70–79, and 80 years and above [14].

To compare the levels of first dose coverage between older and younger populations, we defined a *campaign optimality index* (COI). The COI for age group A at threshold T is denoted as $\eta(A,T)$. The COI measures the proportion of the subsequent (older) age group at the time when the age group A first reached its threshold relative coverage of T. The relative coverage at time t is defined as the coverage at time t divided by the final coverage on September 6, 2021. To compute the optimality index for threshold T and age group $A = [a_{\min},a_{\max}]$ (which includes individuals aged between $a_{\min}$ and $a_{\max}$), we calculated the date when the relative cov$T$ $\eta(A,T)$ as the relative coverage of the subsequent $A_{\text{older}} = [a_{\min} +10,a_{\max} +10]$. We use relative coverages instead of the absolute coverages to avoid biasing COI due to errors in population estimate.

If vaccination was only offered to an age group A when everyone in the subsequent age group was vaccinated, then the COI would be 1. Smaller values of the COI for a particular age group

For peer review only - http://bmjopen.bmj.com/site/about/guidelines.xhtml

indicates that fewer people in the older age group were vaccinated. In an ideal scenario where the vaccination campaign for younger groups only begins after older groups achieve high coverage, the coverage of the older group A_{older} should be close to 100% when the coverage of the younger group reaches T, in which case $\eta(A,T) = 1$. The campaign optimality index was computed for all age groups and using thresholds of 25%, 50% and 75%.

Population that missed their second dose

We estimated the proportion of the population that delayed the second dose, i.e., the proportion did not receive their second dose within the suggested timeframe (21–28 day interval for Coronavac, and 90 days for all other approved 2-dose COVID-19 vaccines). To estimate the proportion of the population that missed the second dose entirely, we determined (i) the proportion of all first dose vaccinated individuals that did not have a second dose recorded, and (ii) from this, selected only those for which the time interval between the first dose and the date of the last entry in the dataset (September 06, 2021) is higher than the suggested time interval between doses for the corresponding vaccine plus a tolerance of 30 days. In other words, this is the proportion of individuals that did not take the second dose and whose second dose is delayed by at least 30 days. Both proportions were estimated for each age, sex, race and municipality. We excluded the single-dose vaccine of Janssen-Cilag from this analysis.

Risk of COVID-19 death by age-group

We used logistic regression to estimate the probability that a SARI patient would die with COVID-19. Due to substantial amount of missing vaccination data (54% of all SARI patients with symptoms onset before September 6th, reaching as high as 87% before March 1st), and uncertainty about the reason the data was missing, we used age group and sex specific vaccination coverage in the individual's municipality of residence as a proxy. For each SARI patient, we used their age group, the socioeconomic conditions, and vaccination coverage of their municipality of residence. We also included a binary indicator for whether the SARI cases' symptoms began before a substantial amount of vaccination was administered to their age group (i.e., if the symptoms occurred before half of the relevant group had been vaccinated.). Supplementary Information 1 have details on the model. We computed odds

For peer review only - http://bmjopen.bmj.com/site/about/guidelines.xhtml

ratio for each regression coefficient β as $OR = e^{\beta}$. Odds ratio for a continuous covariate can be interpreted as the increased risk of death for a unit increase in the covariate.

Risk of COVID-19 hospitalisation by age group

We used negative binomial regression (GLM with a log-link function) to model the risk of SARI hospitalisations per person per day in each state adjusting for age group, whether symptom onset occurred before or after a substantial level of vaccine had been administered (i.e. 50% for the age group and state), and each state's average socioeconomic factors such as the level of unemployment, education, and per capita income. Given data availability, we used the aggregated values for socioeconomic status across each state as a proxy for the individual's values. Supplementary Information 2 have full details of the model.

Age distribution of death by vaccination coverage

To assess the potential protective effect of vaccination coverage by age, we looked at the distribution of ages among COVID-19 deaths in the SARI database. We modelled the ages as a sample from a multinomial distribution with parameter $\theta = (\theta_1,\ldots,\theta_n)$ where θ_j is the probability of a death belonging to the jth age group. The posterior distribution of θ is a Dirichlet distribution with parameters $\alpha = (1 + D_1,\ldots,1 + D_n)$, where D_j is the number of deaths observed in age group j, assuming a non-informative Dirichlet prior distribution with parameters $(1,\ldots,1)$. Sampling from the posterior distribution provided the marginal credible intervals for the proportion of COVID-19 deaths in each age group at four points in the epidemic: Before January 17, January 17 to March 16, March 17 to May 16, and May 17 to September 16, 2021.

Results

Geographical inequalities in vaccination coverage

Between January 17 and September 6, 2021, 133.4 million people were recorded as having had a first dose, corresponding to 84.4% of the population above 18 years of age. We determined the proportion of the population vaccinated with at least one dose in each

For peer review only - http://bmjopen.bmj.com/site/about/guidelines.xhtml

For peer review only

municipality and mapped the distribution of coverage across states (Figure 1A). Similar trends can be observed for the proportion of the population that is fully vaccinated (Figure S1). At the national level, older age groups have higher vaccination coverage, which decreases with age (Figure 1B). A greater proportion of women were vaccinated than men; this difference is more evident among younger age groups below the age of 50 years. Figure 1C shows that there is a clear regional divide in vaccination coverage, with the South and Southeast states having the highest coverage, and the North and Northeast states having the lowest coverage, although there is a substantial amount of variability between the municipalities within each state.

Figure 1. Inequalities in COVID-19 vaccination coverage across Brazil from January 17–September 6, 2021. (A) Percentage of population vaccinated with at least one dose across states as of September 6, 2021. (B) Distribution of vaccination uptake for the first dose between men and women by age group. (C) Vaccination coverage across municipalities (black dot represents the mean coverage).

Premature advancement of the vaccination campaign towards younger groups

Figure 2A shows the proportion of the population vaccinated with a first dose in Brazil, by age group and sex over time. Due to uncertainty in total population size, coverage exceeds 100% for some groups. Similarly, coverages smaller than 100% may not imply the remaining population is not immunized. At the start of the campaign, both healthcare workers and older populations were prioritised for vaccination, hence delayed vaccination for younger groups. The vaccination coverage by sex shows the coverage for men remained consistently lower than women in all age groups over time.

We observed an unanticipated increase in coverage among younger age groups before older groups were fully vaccinated (Figure 2B). This pattern is accentuated in populations below 60 years of age. Using the Campaign Optimality Index (COI), we defined the coverage of older groups when the coverage of younger groups reached a threshold coverage of 25%, 50% and

For peer review only - http://bmjopen.bmj.com/site/about/guidelines.xhtml

8

75%. Larger values indicate a more staggered distribution indicating stronger prioritisation towards older age groups, while lower values indicate that age groups are being vaccinated together (i.e., weaker prioritisation). In Figure 2C, COI is higher for age groups above 50 years old and drops substantially for younger groups. The difference between age groups is greatest for the threshold of 25% and decreases with higher thresholds, with COI being above 80% for all age groups for the threshold of 75%. Even though the vaccination campaign for younger groups started prematurely, the coverage of younger groups crossed the threshold of 75% when coverage for older groups was higher than 80%.

Figure 2. COVID-19 vaccination uptake over time among age groups. (A) Vaccination coverage for the first dose over time. (B) Daily change in vaccination coverage by age group and sex. (C) Campaign optimality index (COI) by age group using threshold vaccination coverage of 25%, 50% and 75%.

Probability of missing the second dose varies by sex, race, and income

By September 6, 2021, 11.9% and 10.2% of the individuals vaccinated with the first dose had their second dose, respectively, delayed and missed. Only 14.3% of individuals that did not take the second dose after 30 days past the recommended date ended up taking a delayed second dose. Among the individuals that were vaccinated with a first dose of Coronavac, 8.78% (95% CI 8.77%–8.80%) missed their second dose. This is low compared to the 12.27% of individuals that missed the second dose (95% CI 12.25%–12.29%) of other vaccines (Covishield and Pfizer), where the optimal interval between the first and second dose is 90 days.

The probability of missing the second dose by race and sex is shown in Figure 3A. Men were more likely to miss (Relative Risk (RR) 1.065, 95% CI 1.063–1.067) the second dose than women. Indigenous Brazilians were also 2.64 (95% CI 2.62–2.66) times more likely to miss the second dose than White Brazilians, followed by East Asian Brazilians (RR 1.68, 95% CI 1.68–1.69), Pardos (RR 1.65, 95% CI 1.65–1.65) and Black Brazilians (RR 1.51, 95% CI 1.50–1.51).

For peer review only - http://bmjopen.bmj.com/site/about/guidelines.xhtml

For peer review only

The probability of missing the second dose varies with income per capita (Figure B). Individuals in municipalities with a lower income per capita are more likely to miss their second dose. The median income per capita for municipalities with a 2 % probability of skipping the second dose is 394.95 US$ Purchasing Power Parity (PPP) 2010 (IQR 303.75 - 468.66). This decreases to 194.81 US$ PPP 2010 (IQR 153.17 - 303.17) when the probability of skipping is more than 10%. Figure C shows the variation in the probability of missing the second dose by state.

Figure 3. Probability of missing the second dose of the COVID-19 vaccine. (A) Probability of missing the second dose based on three factors: vaccine type, race and sex. (B) Probability of having a second dose missed calculated at municipality level in terms of the income per capita decile. (C) Probability of missing the second dose calculated for each municipality and grouped by state.

Vaccination decreases age-adjusted risk of hospitalization and death

Vaccination decreases the risk of hospitalisation or death due to COVID-19 after adjusting for age group and socioeconomic conditions. Using the age group, 20 years of age and under as a baseline, the risk of COVID-19 death increased with age. For instance, the odds of death are almost ten times greater for someone above the age of 50 years (OR: 9.7–29.0, 95% CI: 9.4, 29.9) than an individual under 20 years of age. In general, patients living in municipalities with higher first-dose vaccination coverage had lower risk of death (OR: 0.820, 95% CI: 0.812, 0.827). Patients living in municipalities with higher proportion of primary education attainment (OR: 0.231, 95% CI: 0.217, 0.246) had reduced risk of death. Patients from municipalities with higher unemployment rates were at a higher risk of death (OR: 2.29, 95% CI: 2.14, 2.45). An increase of income per capita of 100 US$ PPP decreased the risk of death (OR: 0.961, 95% CI: 0.960 - 0.963). Our sensitivity analysis at the individual level which includes only patients with a recorded vaccination status, showed that patients were at a lower risk of death after being vaccinated with at least one dose. While similar patterns for age groups were observed for hospitalisations, a higher proportion of primary education attainment was associated with higher risk of hospitalisations. Detailed

For peer review only - http://bmjopen.bmj.com/site/about/guidelines.xhtml

information on model outputs can be found in Supplementary Information 3, Table S1 and Table S2.

To verify the relationship between vaccination coverage and risk of death by age groups, we plotted the probability of a patient dying from COVID-19 being in a particular age group prior to January 17, 2021 and after the start of the vaccination campaign (including and after January 17, 2021) (Figure 4A). There is a higher probability that those dying before the start of the vaccination campaign were from an older age group. After vaccination began, those dying were more likely to be from age groups with ages below 60 years. This can be attributed to vaccination, where the proportion of vaccination is much higher among people in older age groups due to earlier uptake (Figure 4B). However, individuals under 20 years of age showed a reverse trend, where the proportion of deaths was higher prior to the vaccination campaign.

Figure 4. Risk of SARI death and vaccination coverage by age distribution. (A) Distribution of SARI hospitalisations over time (March 2020–September 2021). (B) The distribution of age among SARI-linked deaths at four points in time indicates that as the epidemic progressed SARI deaths were more likely to be younger individuals. (C) Proportion of population vaccinated with at least one dose by age group during study period.

Discussion

The Brazilian COVID-19 vaccination campaign led to a decrease in severe cases and deaths. This is further supported by real-world cohort data (C.V.B Dos Santos et al., 2023). Notwithstanding, the campaign faced issues with rollout delays and vaccine shortages which effected timing and implementation [15], curtailing effectiveness. Inequalities exist in COVID-19 vaccination coverage, especially between states and different age, sex, and socioeconomic groups. Among patients diagnosed with COVID-19, the risks of hospitalisation and death are significantly greater among older individuals and those with lower socioeconomic status. Increasing vaccination reduces the risk of death, especially

For peer review only - http://bmjopen.bmj.com/site/about/guidelines.xhtml

297 among older age groups. Vaccine uptake is greater in areas where hospitalisation rates were
298 greater, suggesting better access to healthcare and improved reporting of infections.

299 Variation in vaccination between population groups was mainly driven by the unequal
300 distribution of vaccines between states. States from the North and Northeast regions, such
301 as Amazonas, Amapá, and Ceará, were prioritised for vaccination in January 2021 due to high
302 transmission rates of the P.1 (Gamma) variant, which was first detected in Manaus (the
303 capital of Amazonas) in January 2021 (Buss et al. 2021). Despite this, lower overall coverage
304 for the first dose was observed in the North and Northeast regions of Brazil. These regions
305 are economically less developed and have experienced scare hospital resources during the
306 pandemic (Rocha et al. 2021). Delays in notification of vaccinations had an important impact
307 on coverage between states. Even though we determined a 37.5% first-dose coverage for the
308 northeastern state of Ceará, official statistics obtained directly from the Brazilian state
309 secretariats on September 6, 2021 found this was 59.2%. The low COI obtained with a
310 threshold of 25% for age groups younger than 50 shows the vaccination campaign started
311 prematurely for these groups, which may have impeded vaccination of older groups. It is
312 worth noting that the proposed COI only considers age as a priority criterion and does not
313 include other criteria such as comorbidities or employment (e.g., being a healthcare worker).

314 Inequalities in uptake are evident between the sexes. Women were more likely to have
315 received both doses than men. This may be owing to the early stages of the vaccination
316 campaign, when healthcare workers were prioritised: women make up a significant
317 proportion of healthcare workers in Brazil, accounting for 62.5% of all vaccinated healthcare
318 workers, which corresponds to 10.6% of all vaccinated individuals in our dataset. Moreover,
319 women seek out healthcare more frequently than men. Based on a survey study with over
320 170,000 responses, men showed greater hesitancy towards COVID-19 vaccines, indicating
321 the main reason being fear of severe clinical reactions [16]. We also found a longer delay for
322 taking the second dose in municipalities with lower income per capita, which aligns with
323 previous findings that low incomes were associated with higher rates of vaccine hesitancy
324 during the pandemic [16]. This has implications for death risk, as our findings show that SARI
325 patients from poor municipalities are more likely to die from COVID-19 than those from
326 wealthier municipalities.

For peer review only - http://bmjopen.bmj.com/site/about/guidelines.xhtml

Vaccination reduces the risk of hospitalization and death. The positive association between education and hospitalisation could suggest greater accessibility to healthcare in areas with more COVID-19 infection awareness. To further investigate whether vaccination can prevent COVID-19 related deaths for each age group, we considered the age distribution of those that died, across multiple periods of time. We observed a reduction in the proportion of mortality in older age groups once vaccination became available. This trend was the least evident with young people under the age of 20. This was likely owing to the delayed vaccination of young people until July 2021. Due to a lack of complete population-level data, it may be premature to surmise the true protective effects of vaccination coverage on younger age groups. While the overall risk of death is lower for young people, COVID-19 could still pose a threat, regardless of pre-existing medical conditions [17,18].

Our estimates of vaccination coverage by state and age-group are limited by the accuracy of the projected population estimates. Our data for socioeconomic conditions came from the 2011 census, since the 2020 census was not collected and the delayed 2022 census results are not yet available. Further, current socioeconomic conditions may have diverged within states since 2011 due to the variation in state-level funding for social welfare in Brazil [19]. Comprehensive vaccination data of individual SARI patients was not available so we had to rely on overall vaccination coverage for each age group and municipality; this may have introduced discrepancies in the modelled relationship between vaccination coverage and the risk of death. Due to these limitations, we were limited to an ecological study. Human error affects the accuracy of vaccination records (e.g. date of vaccination could be replaced by the date when the information was entered into the system rather than administration date). Moreover, the lack of healthcare resources in some areas (e.g. Ceará) may have led to lower levels of case notification and notification delay. Self-identification of race may be prone to bias, with wealthier Black Brazilian more inclined to self-identify and contribute race information than poorer Black Brazilians [20].

Although there exists some vaccine hesitancy among certain demographic groups, the overall intention to be vaccinated against COVID-19 is high [16,21]. Vaccine hesitancy is a complex issue; a survey from late 2020 found that when certain countries of origin for the vaccine were mentioned (e.g., China and Brazil), the willingness to vaccinate among

For peer review only - http://bmjopen.bmj.com/site/about/guidelines.xhtml

Brazilians decreased. However, our study shows that individuals that were vaccinated with the first dose of Coronavac were less likely to skip the second dose when compared with individuals that were vaccinated with other vaccines, such Covishield or Pfizer. This may be partially attributed to the fact that the optimal interval between the first and second doses for most vaccines is 90 days, while the interval for Coronavac is only 21 to 28 days [22]. The disparities in vaccination coverage may have amplified the inequalities in risk of death during the epidemic in Brazil, as the Gamma variant is associated with an increased infection fatality rate [8].

Despite the allocation of vaccines nationwide and the consideration of priority groups, it is evident from our study that there were several inequality-generating processes within the Brazilian COVID-19 vaccination programme. In general, national vaccine programmes typically focus on controlling for medical conditions between population groups [23]. Our findings demonstrate that to reduce health inequalities, equitable vaccine distribution should address demographic and socioeconomic inequalities [24]. To tackle inequality in uptake, vaccination campaigns should aim to understand the mechanisms that lead to vaccine hesitancy. For instance, reasons for missing the second dose could be attributed to perceived politicization of the campaign, distrust in pharmaceutical services, and the lack of awareness of the long-term efficacy in Brazil [25].

The third booster dose has been controversial, as parts of the population were still awaiting their second dose. Vaccination has a protective effect on populations but the details of how this is communicated to the public are complex. Our findings on inequalities in coverage and on demographic groups that are more willing to miss the second dose should be considered when designing media campaigns and interventions to increase vaccine access and uptake.

For peer review only

Contributorship statement

Sabrina L. Li - Conceptualization, Data Curation, Formal Analysis, Investigation, Methodology, Software, Validation, Visualization, Writing – Original Draft Preparation, Writing – Review & Editing

For peer review only - http://bmjopen.bmj.com/site/about/guidelines.xhtml

Carlos A. Prete Jr - Conceptualization, Data Curation, Formal Analysis, Investigation, Methodology, Software, Validation, Visualization, Writing – Original Draft Preparation, Writing – Review & Editing

Alexander E. Zarebski - Conceptualization, Formal Analysis, Methodology, Software, Validation, Writing – Original Draft Preparation, Writing – Review & Editing

Andreza A de Souza Santos – Investigation, Validation, Writing – Review & Editing

Ester C Sabino - Investigation, Validation, Writing – Review & Editing

Vitor H Nascimento - Formal Analysis, Validation, Writing – Review & Editing

Chieh-Hsi Wu - Formal Analysis, Validation, Writing – Review & Editing

Jane P Messina - Conceputalization, Writing – Review & Editing, Supervision

Competing Interests

None declared.

Funding

SLL is supported by the Oxford Martin Programme on Pandemic Genomics and the Canadian Social Sciences and Humanities Research Council (SSHRC) Doctoral Fellowship. CAPJ was supported by FAPESP (2019/21858-0 and 2022/15985-1). AEZ is supported by the Oxford Martin School Programme on Pandemic Genomics. CAPJ and VHN were supported by Coordenação de Aperfeicoamento de Pessoal de Nível Superior – Brasil (CAPES) – Finance Code 001. VHN is supported by the Brazilian National Council for Scientific and Technological Development (CNPq: 304714/2018-6). ECS is supported by a

For peer review only

For peer review only - http://bmjopen.bmj.com/site/about/guidelines.xhtml

UKRI Medical Research Council-São Paulo Research Foundation (FAPESP) CADDE partnership award (MR/S0195/1 and FAPESP 18/14389-0) (http://caddecentre.org/).

Data sharing statement

Data are available upon reasonable written request to the authors.

References

1 Domingues CMAS. Desafios para a realização da campanha de vacinação contra a COVID-19 no Brasil. *Cad Saúde Pública*. 2021;37:e00344620.

2 Li SL, Pereira RHM, Jr CAP, *et al.* Higher risk of death from COVID-19 in low-income and non-White populations of São Paulo, Brazil. *BMJ Glob Health*. 2021;6:e004959.

3 Hallal PC, Victora CG. Overcoming Brazil's monumental COVID-19 failure: an urgent call to action. *Nat Med*. 2021;27:933–933. doi: 10.1038/s41591-021-01353-2

4 The dissemination of the Omicron variant in the highly seroprevalent Amazonas state, Brazil, is associated with a rapid upsurge of SARS-CoV-2 cases - SARS-CoV-2 coronavirus / nCoV-2019 Genomic Epidemiology. Virological. 2022. https://virological.org/t/the-dissemination-of-the-omicron-variant-in-the-highly-seroprevalent-amazonas-state-brazil-is-associated-with-a-rapid-upsurge-of-sars-cov-2-cases/785 (accessed 1 November 2023)

5 Confederação Nacional de Municípios. "Pesquisa CNM — Covid-19." www.cnm.org.br/cms/biblioteca/Pesquisa-CNM-Covid-19_Ed28.pdf.

6 Bolcato M, Rodriguez D, Feola A, *et al.* COVID-19 Pandemic and Equal Access to Vaccines. *Vaccines*. 2021;9:538. doi: 10.3390/vaccines9060538

7 Ye Y, Zhang Q, Wei X, *et al.* Equitable access to COVID-19 vaccines makes a life-saving difference to all countries. *Nat Hum Behav*. 2022;6:207–16. doi: 10.1038/s41562-022-01289-8

8 Prete CA Jr, Buss LF, Whittaker C, *et al.* SARS-CoV-2 antibody dynamics in blood donors and COVID-19 epidemiology in eight Brazilian state capitals: A serial cross-sectional study. *eLife*. 2022;11:e78233.

9 Lana RM, Freitas LP, Codeço CT, *et al.* Identificação de grupos prioritários para a vacinação contra COVID-19 no Brasil. *Cad Saúde Pública*. 2021;37:e00049821.

10 O'Driscoll M, Ribeiro Dos Santos G, Wang L, *et al.* Age-specific mortality and immunity patterns of SARS-CoV-2. *Nature*. 2021;590:140–5. doi: 10.1038/s41586-020-2918-0

For peer review only - http://bmjopen.bmj.com/site/about/guidelines.xhtml

11 Hitchings MDT, Ranzani OT, Torres MSS, *et al.* Effectiveness of CoronaVac among healthcare workers in the setting of high SARS-CoV-2 Gamma variant transmission in Manaus, Brazil: A test-negative case-control study. *Lancet Reg Health - Am*. 2021;1:100025.

12 de Souza WM, Buss LF, Candido D da S, *et al.* Epidemiological and clinical characteristics of the COVID-19 epidemic in Brazil. *Nat Hum Behav*. 2020;4:856–65. doi: 10.1038/s41562-020-0928-4

13 Instituto Brasileiro de Geografia e Estatística. Censo Demográfico. https://www.ibge.gov.br/estatisticas/sociais/populacao/9662-censo-demografico-2010.html?=&t=o-que-e (accessed 1 November 2023)

14 Freire FHM de A, Gonzaga MR, Gomes MMF. Projeções populacionais por sexo e idade para pequenas áreas no Brasil. *Rev Latinoam Poblac*. 2020;14:124–49. doi: 10.31406/relap2020.v14.i1.n26.6

15 Ferreira LS, Darcie Marquitti FM, Paixão da Silva RL, *et al.* Estimating the impact of implementation and timing of the COVID-19 vaccination programme in Brazil: a counterfactual analysis. *Lancet Reg Health Am*. 2023;17:100397.

16 Moore DCBC, Nehab MF, Camacho KG, *et al.* Low COVID-19 vaccine hesitancy in Brazil. *Vaccine*. 2021;39:6262–8.

17 Cunningham JW, Vaduganathan M, Claggett BL, *et al.* Clinical Outcomes in Young US Adults Hospitalized With COVID-19. *JAMA Intern Med*. 2021;181:379–81.

18 Taylor L. Covid-19: Brazil's spiralling crisis is increasingly affecting young people. *BMJ*. 2021;373:n879.

19 Stankiewicz Serra A, Yalonetzky GI, Maia AG. Multidimensional Poverty in Brazil in the Early 21st Century: Evidence from the Demographic Census. *Soc Indic Res*. 2021;154:79–114.

20 Telles E, Paschel T. Who Is Black, White, or Mixed Race? How Skin Color, Status, and Nation Shape Racial Classification in Latin America. *Am J Sociol*. 2014;120:864–907.

21 Gramacho WG, Turgeon M. When politics collides with public health: COVID-19 vaccine country of origin and vaccination acceptance in Brazil. *Vaccine*. 2021;39:2608–12.

22 Ministério da Saúde. Plano Nacional de Operatcionalização Da Vacinação Contra a COVID-19. Brasília - DF. 2021.

23 Mamelund S-E, Dimka J. Social inequalities in infectious diseases. *Scand J Public Health*. 2021;49:675–80.

For peer review only

478 24 Rydland HT, Friedman J, Stringhini S, *et al.* The radically unequal distribution of Covid-19 vaccinations: a predictable yet avoidable symptom of the fundamental causes of inequality. *Humanit Soc Sci Commun*. 2022;9:1–6. doi: 10.1057/s41599-022-01073-z

481 25 Santos KCO dos, Junqueira-Marinho M de F, Reis AT, *et al.* Social Representations of Hesitant Brazilians about Vaccination against COVID-19. *Int J Environ Res Public Health*. 2023;20:6204. doi: 10.3390/ijerph20136204

484

For peer review only

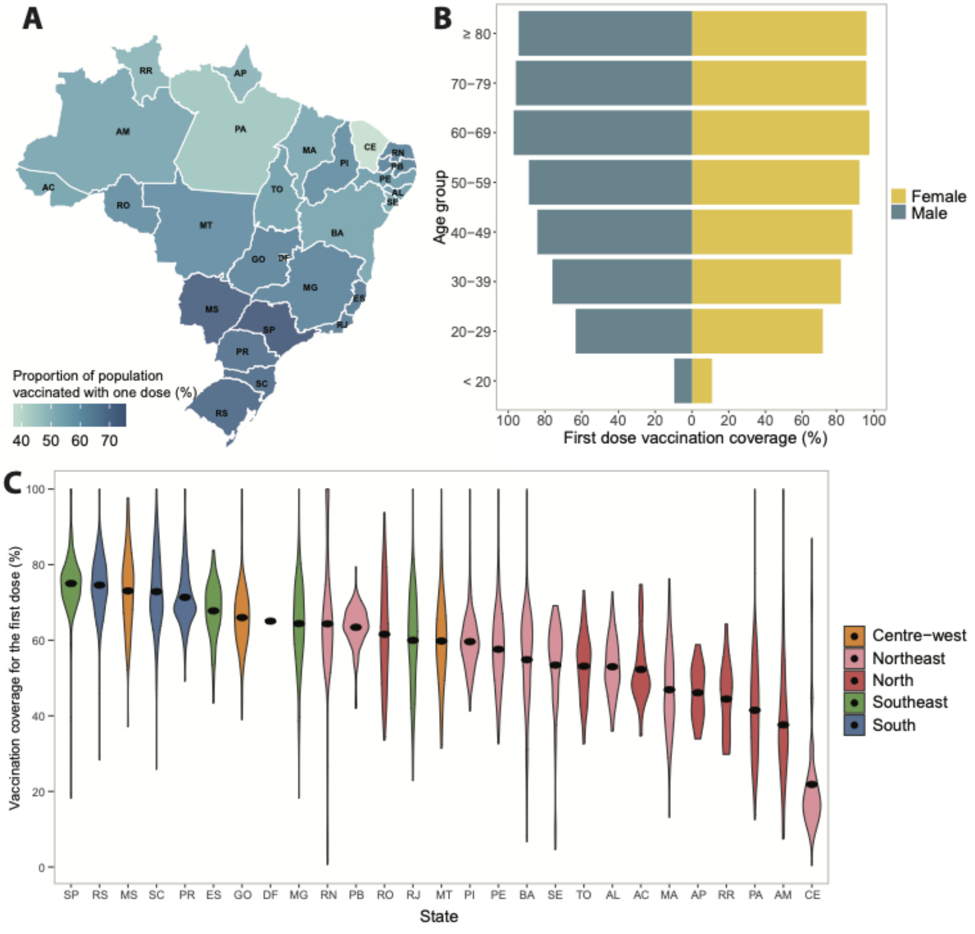

Fig 1

274x259mm (72 x 72 DPI)

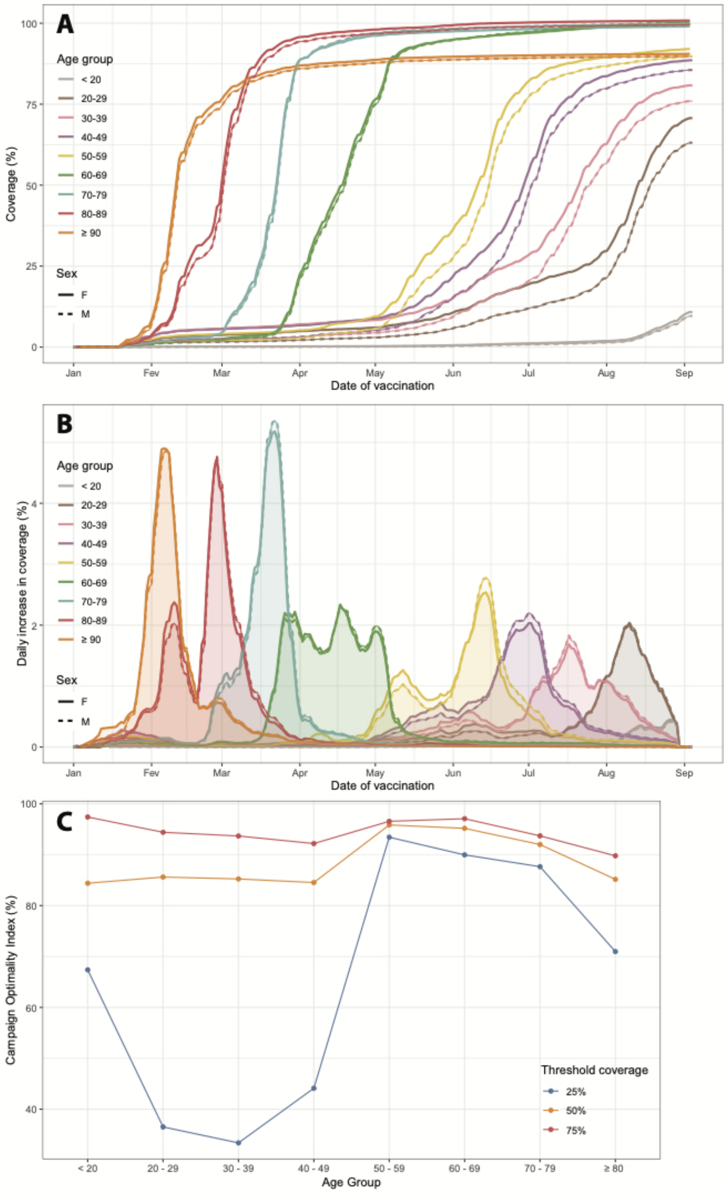

Fig 2

234x381mm (72 x 72 DPI)

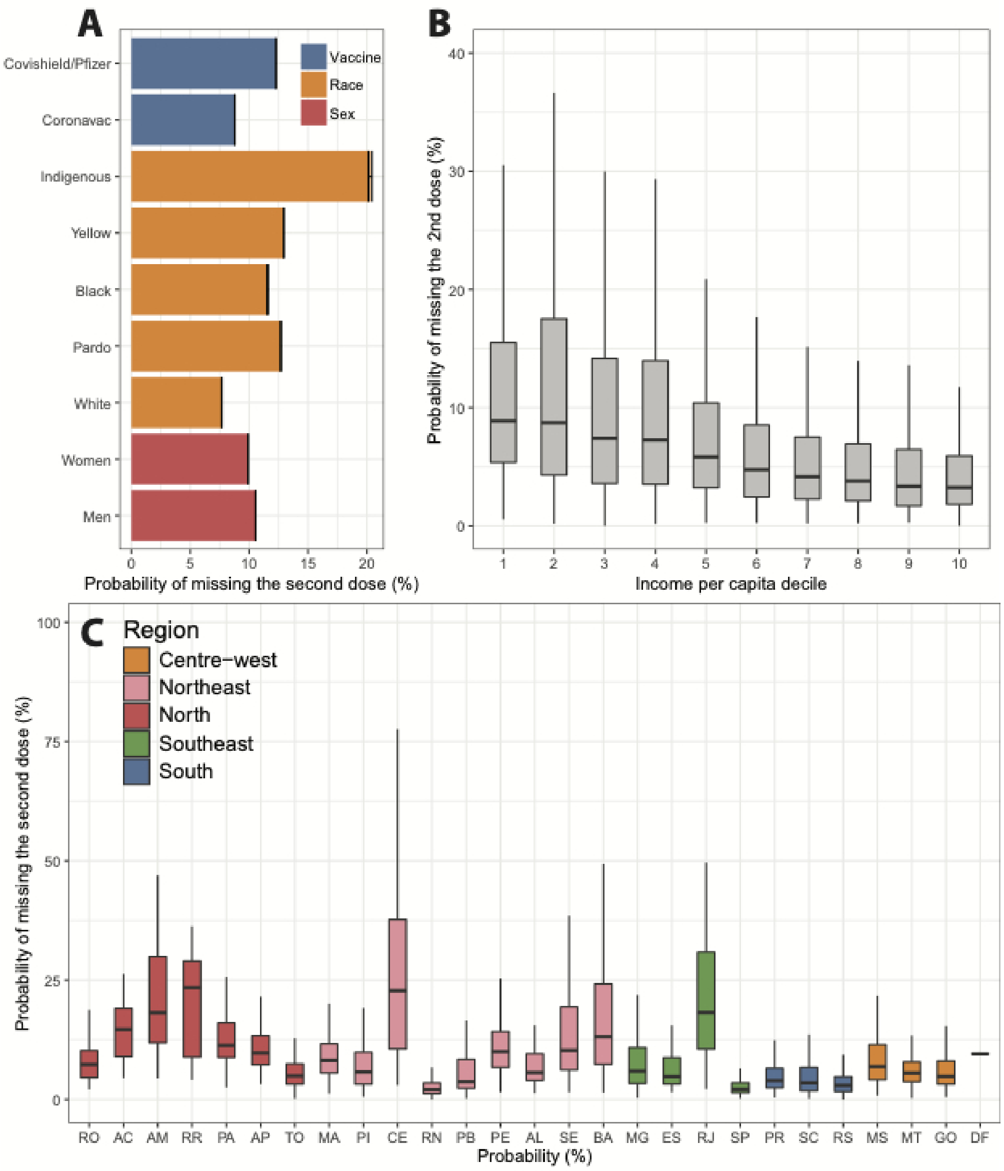

Fig 3

219x257mm (72 x 72 DPI)

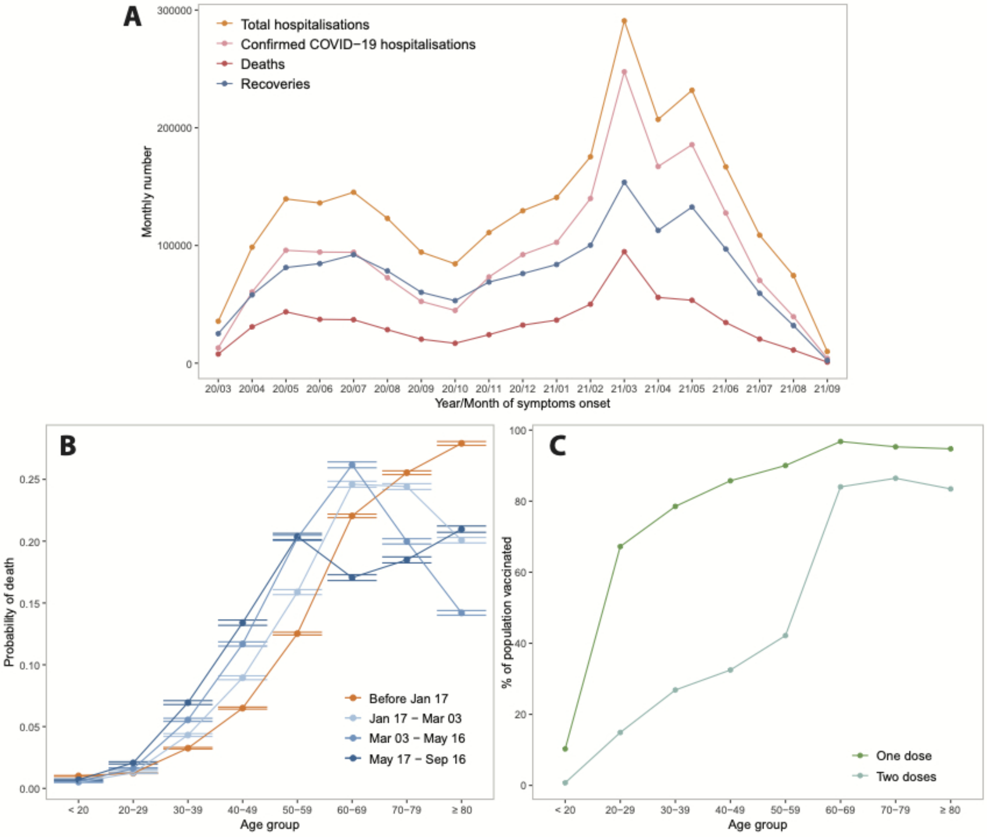

Fig 4

302x255mm (72 x 72 DPI)

Mortality

Logistic regression analysis of mortality

2023-10-27

For peer review only

Table of contents

Packages . . . 1
Data files . . . 2
    Output files . . . 2
    Input files . . . 2
    Check data files exist . . . 2
Satellite cities and excluded municipality codes . . . 3
Socio-economic (census) data . . . 4
Vaccination campaign data . . . 5
    Old preprocessing steps . . . 5
Vaccination coverage data . . . 6
    Old preprocessing steps . . . 6
SIVEP-Gripe dataset . . . 7
    Preprocessing function . . . 7
    Preparing the data . . . 8
Combined dataframe . . . 9
GLM . . . 11
    LaTeX table summarising the model fit . . . 13
Session information . . . 14

Packages

```
library(dplyr)
library(readr)
library(lubridate)
library(car)
```


For peer review only - http://bmjopen.bmj.com/site/about/guidelines.xhtml

```
library(xtable)
```

- The `readr` package provides a function, `read_delim` that is faster than the built-in `read.csv` function and provides a progress bar.
- The `car` package provides the `vif` function which we use to check for a concerning about of covariation between the covariates of our model.
- The `xtable` package is needed to make a LaTeX table summarising the final model fit.

Data files

Output files

```
output_files <- list(
  final_dataset = "out/final-mortality-dataset.csv",
  final_model_table = "out/final-mortality-model.tex"
)
```

Input files

Data files live in `data/`.

```
input_data <- list(
  sivep_2020 = "data/sivep_2020.csv",
  sivep_2021 = "data/sivep_20092021.csv",
  socio_econ_2010 = "data/census2010_muni_covariates.csv",
  vaccination_dates = "out/vaccination-halfway-dates.rds",
  vaccination_coverage = "out/vaccination-age-muni-date-counts.rds"
  )
```

Check data files exist

```
assert_file_exists <- function(fp) {
  if (!file.exists(fp)) {
    stop(sprintf("Cannot find file: %s", fp))
  }
}
```


For peer review only - http://bmjopen.bmj.com/site/about/guidelines.xhtml

```
lapply(X = input_data, FUN = assert_file_exists)
```

```
$sivep_2020
NULL

$sivep_2021
NULL

$socio_econ_2010
NULL

$vaccination_dates
NULL

$vaccination_coverage
NULL
```

If you want to check that the files you have match the ones used, here are some checksums (which can be obtained with `sha256sum *` from within `data/`.

```
9116d21e84b8ab4d4e8b2437644248618dc39affc7f6101d8ac7c631950c2e8e  agesex_coverage_muni_new.cs
7a5a6d07ab0beadd1c015d087802192aa02a227fad72ab33d8e0299600db29b0  census2010_muni_covariates
e7db11cdf762b65b51df22ab4a71bf6f57f20d503ec568f34788c62bd5e0073f  dates_50.rds
2ee48a45bb88d3b78fa9c5571918d5bce23aa135fc3234ff1e1cc3836cfb4f32  sivep_20092021.csv
4c7bfe45057871a354ef00ab7a905b1daafff844dbd0d89045f8ed8c5f06c67a  sivep_2020.csv
```

Satellite cities and excluded municipality codes

The following `aggregated_satellites` function is taken from the preprocessing script.

> We exclude the municipality with code 431453 as it has a transient existence and 999999 since it is a malformed value. Since the codes are variously integers or characters we will filter these out on a case by case basis.
>
> Municipality codes starting with 53 are cities from DF. They are cities but not municipalities (they are called satellite cities). We substitute Brasilia's code (530010) for all of them. The following function helps with this.

```
aggregated_satellites <- function(muni_codes) {
  code_class <- class(muni_codes)
```


For peer review only - http://bmjopen.bmj.com/site/about/guidelines.xhtml

```
  stopifnot(
    is.element(el = code_class, set = c("integer", "character"))
  )
  if (code_class == "integer") {
    ifelse(round(muni_codes / 10000) == 53, 530010, muni_codes)
  } else {
    gsub(x = muni_codes,
         pattern = "53[0-9]{4}",
         replacement = "530010")
  }
}
```

Socio-economic (census) data

Remove the final digit from the municipality code associated with the municipality. The first digits are sufficient to uniquely identify them.

This dataset contains the measurements of the following quantities:

- unemployed,
- informal,
- edu_primary_lower,
- avg_household_income_capita,
- and patient_address_muni_code which is used for the join.

```
socio_econ_df <-
  input_data$socio_econ_2010 |>
  read_csv() |>
  mutate(patient_address_muni_code = aggregated_satellites(
           gsub("[0-9]$", "", code_muni)
         )) |>
  filter(patient_address_muni_code != "431453",
         patient_address_muni_code != "999999") |>
  select(-code_muni)
```

```
Rows: 5565 Columns: 5
-- Column specification --------------------------------------------------------
Delimiter: ","
dbl (5): code_muni, unemployed, informal, edu_primary_lower, avg_household_i...
```

For peer review only

For peer review only - http://bmjopen.bmj.com/site/about/guidelines.xhtml

```
i Use `spec()` to retrieve the full column specification for this data.
i Specify the column types or set `show_col_types = FALSE` to quiet this message.
```

Vaccination campaign data

Because in the preprocessing we used integers to represent age ranges, and in this script we use strings describing the range, it is helpful to have a function to change from the integers to the strings

```
age_group_int_to_string <- function(age_groups) {
  case_when(age_groups == 19 ~ "ageunder20",
            age_groups == 20 ~ "age20to29",
            age_groups == 30 ~ "age30to39",
            age_groups == 40 ~ "age40to49",
            age_groups == 50 ~ "age50to59",
            age_groups == 60 ~ "age60to69",
            age_groups == 70 ~ "age70to79",
            age_groups == 80 ~ "age80_plus")
}
```

We can read the data of when the vaccination campaign reached half coverage (for an age group in a specific municipality) with the following and rename the columns to match the rest of the code.

```
vac_dates_df <-
  input_data$vaccination_dates |>
  readRDS() |>
  mutate(age_group = age_group_int_to_string(age_group),
         municipality_code = as.character(municipality_code)) |>
  rename(patient_address_muni_code = municipality_code,
         vac_start_date = half_vac_date)
```

N.b. since we already handled the questionable municipality codes in the preprocessing script we do not need to worry about this again.

Old preprocessing steps

Here is an older version of the code to do this processing step, but the values are coming out slightly different which is a bit concerning.

For peer review only - http://bmjopen.bmj.com/site/about/guidelines.xhtml

```r
vac_dates_df <-
  "dates_50.rds" |>
  readRDS() |>
  filter(age.lower != 90) |>
  mutate(age_group = case_when(age.lower == "< 20" ~ "ageunder20",
                               age.lower == 20 ~ "age20to29",
                               age.lower == 30 ~ "age30to39",
                               age.lower == 40 ~ "age40to49",
                               age.lower == 50 ~ "age50to59",
                               age.lower == 60 ~ "age60to69",
                               age.lower == 70 ~ "age70to79",
                               age.lower == 80 ~ "age80_plus"),
         patient_address_muni_code = as.character(mun_vac)) |>
  rename(vac_start_date = t50) |>
  as.data.frame() |>
  select(age_group,
         patient_address_muni_code,
         vac_start_date)
```

We need to coerce this to a data frame towards the end because otherwise it complains that the grouping column `mun_vac` is no longer present, even though it is, we have just renamed it.

Vaccination coverage data

Because the vaccination coverage was calculated from estimates of population size it is possible that it takes values greater than `1.0`. When this happens we need to clip the values down to `1.0`.

```r
vac_cov_df <-
  input_data$vaccination_coverage |>
  readRDS() |>
  mutate(age_group = age_group_int_to_string(age_group),
         municipality_code = as.character(municipality_code)) |>
  rename(patient_address_muni_code = municipality_code)
```

Old preprocessing steps

Here is an older version of the code to do this processing step:

For peer review only - http://bmjopen.bmj.com/site/about/guidelines.xhtml

```
vac_cov_df <-
  "data/agesex_coverage_muni_new.csv" |>
  read.csv() |>
  mutate(agesex_coverage = pmin(agesex_coverage, 1),
         patient_address_muni_code = as.character(patient_address_muni_code)) |>
  select(patient_address_muni_code,
         patient_sex,
         age_group,
         agesex_coverage)
```

SIVEP-Gripe dataset

Preprocessing function

There are a couple of transformations that we want to apply to both the 2020 and 2021 SIVEP-Gripe datasets, so we should abstract them into a function to do this. In the filter we have the following conditions:

- CLASSI_FIN %in% c(4, 5, 9, NA) to select the cases that are probably COVID-19. Note that an earlier version of the code excluded 9 even though they only make up a very small number of cases.
- SEM_PRI >= 10 to only keep instances where the symptom onset is after epi-week 10.
- SG_UF means that we require the patient to have their address state known.

```
preprocess_sivep <- function(sivep_df) {
  sivep_df |>
    filter(CLASSI_FIN %in% c(4, 5, 9, NA),
           SEM_PRI >= 10,
           SG_UF != "") |>
    rename(patient_address_muni_code = CO_MUN_RES,
           patient_sex = CS_SEXO) |>
    mutate(date_symptoms = dmy(DT_SIN_PRI),
           died_boolean = case_when(EVOLUCAO == 2 ~ 1, TRUE ~ 0),
           age_group = case_when(
             NU_IDADE_N < 20 ~ "ageunder20",
             NU_IDADE_N  >= 20 & NU_IDADE_N < 30 ~ "age20to29",
             NU_IDADE_N  >= 30 & NU_IDADE_N < 40 ~ "age30to39",
             NU_IDADE_N  >= 40 & NU_IDADE_N < 50 ~ "age40to49",
             NU_IDADE_N  >= 50 & NU_IDADE_N < 60 ~ "age50to59",
             NU_IDADE_N  >= 60 & NU_IDADE_N < 70 ~ "age60to69",
             NU_IDADE_N  >= 70 & NU_IDADE_N < 80 ~ "age70to79",
```


For peer review only - http://bmjopen.bmj.com/site/about/guidelines.xhtml

```
                NU_IDADE_N  >= 80                  ~ "age80_plus"),
              patient_address_muni_code = aggregated_satellites(
                as.character(patient_address_muni_code)
              )) |>
      filter(patient_address_muni_code != "431453",
              patient_address_muni_code != "999999") |>
      select(age_group,
              date_symptoms,
              died_boolean,
              patient_address_muni_code,
              patient_sex)
  }
```

N.b. we need to do the processing on the municipality codes again here because this is raw data that has not been processed at all yet.

Preparing the data

The only difference in the processing of these data is that for the 2021 dataset we limit ourselves to cases with a symptom onset before 7 September 2021. This preprocessing step takes about a minute on my laptop.

```
sivep_2020_df <-
  input_data$sivep_2020 |>
  read_delim(delim = ";", show_col_types = FALSE) |>
  preprocess_sivep()
```

```
Warning: One or more parsing issues, call `problems()` on your data frame for details,
e.g.:
  dat <- vroom(...)
  problems(dat)
```

```
sivep_2021_df <-
  input_data$sivep_2021 |>
  read_delim(delim = ";", show_col_types = FALSE) |>
  preprocess_sivep() |>
  filter(date_symptoms < ymd("2021-09-07"))
```


For peer review only - http://bmjopen.bmj.com/site/about/guidelines.xhtml

```
Warning: One or more parsing issues, call `problems()` on your data frame for details,
e.g.:
  dat <- vroom(...)
  problems(dat)
```

```
sivep_df <- rbind(sivep_2020_df, sivep_2021_df)
```

The data frames `sivep_2020_df` and `sivep_2021_df` are about 50Mb each, this isn't a huge strain on my laptop, but since I have managed to crash R using these data before, I think it is probably a good idea to remove them since they are no longer needed.

```
rm(sivep_2020_df)
rm(sivep_2021_df)
gc()
```

```
           used  (Mb) gc trigger   (Mb)  max used   (Mb)
Ncells   985279  52.7    2178226  116.4   1781855   95.2
Vcells 60690315 463.1  395988225 3021.2 641652642 4895.5
```

Combined dataframe

We want to be able to filter the cases based on the state of the vaccination campaign so we need to join these data frames so we have the information linked properly.

First we start by joining the data frame that contains a Boolean reflecting if at least 50% of the age group in that municipality had been vaccinated.

```
tmp <-
  left_join(
    sivep_df,
    vac_dates_df,
    by = c("patient_address_muni_code", "age_group")
  ) |>
  mutate(vac_campaign = ifelse(date_symptoms < vac_start_date,
                               "pre", "post"))
stopifnot(!any(is.na(tmp)))
```

The next step is to join in the vaccination coverage as a proportion of the age group in the municipality on the date of symptom onset.

For peer review only - http://bmjopen.bmj.com/site/about/guidelines.xhtml

```
vac_cov_renamed_df <- rename(vac_cov_df, date_symptoms = date)
tmp2 <- left_join(
  tmp, vac_cov_renamed_df,
  by = c("age_group", "patient_address_muni_code", "date_symptoms")
)
rm(vac_cov_renamed_df)
pre_vac_mask <- tmp2$date_symptoms <= ymd("2021-01-16")
tmp2[pre_vac_mask, ]$proportion_vaccinated <- 0.0
```

Because there are not going to be any coverages from before the start of vaccination and there will be cases from this time, those records will get an `NA` for that variable so we need to fill those in with zero because that is the true value in that case. There are only 134 so it is a extremely small amount of corrupted data.

```
print(nrow( filter(vac_cov_df, date < as.Date("2021-01-17"))))
```

```
[1] 134
```

Finally we put all of the data into a single data frame so we can give it to the `glm` function later.

```
final_data_df <-
  left_join(tmp2, socio_econ_df, by = c("patient_address_muni_code")) |>
  mutate(age_group = relevel(as.factor(age_group), ref = "ageunder20"),
         vac_campaign = relevel(as.factor(vac_campaign), ref = "pre"))
```

We will save a copy of this data frame, not to use in the analysis, but so there is a record of it in case we need to check it later.

```
write.table(x = final_data_df,
            file = output_files$final_dataset,
            sep = ",",
            row.names = FALSE)
```

For future reference, the hash of this file is given below.

```
52b1bb1ec523b67f093d5e8bd9cb5821221729921e0cc104526f2d015f2b6325  out/final-mortality-datase
```


For peer review only - http://bmjopen.bmj.com/site/about/guidelines.xhtml

GLM

We remove the `vac_campaign` variable because it is highly colinear with the proportion vaccinated. Including it flips the effect of vaccination because of the colinearity, this is a cleaner model.

```
logit_fit <- glm(died_boolean ~ age_group +
                   proportion_vaccinated +
                   ## vac_campaign +
                   edu_primary_lower +
                   avg_household_income_capita +
                   unemployed,
                 family=binomial(link = "logit"),
                 data=final_data_df)
```

We want a summary table and the confidence intervals on the estimates for the manuscript so we should print these.

```
summary(logit_fit)
```

```
Call:
glm(formula = died_boolean ~ age_group + proportion_vaccinated +
    edu_primary_lower + avg_household_income_capita + unemployed,
    family = binomial(link = "logit"), data = final_data_df)

Coefficients:
                             Estimate Std. Error z value Pr(>|z|)
(Intercept)                -3.011e+00  2.015e-02 -149.41   <2e-16 ***
age_groupage20to29          1.071e+00  1.863e-02   57.46   <2e-16 ***
age_groupage30to39          1.432e+00  1.619e-02   88.50   <2e-16 ***
age_groupage40to49          1.846e+00  1.553e-02  118.83   <2e-16 ***
age_groupage50to59          2.275e+00  1.525e-02  149.16   <2e-16 ***
age_groupage60to69          2.763e+00  1.519e-02  181.87   <2e-16 ***
age_groupage70to79          3.087e+00  1.525e-02  202.42   <2e-16 ***
age_groupage80_plus         3.367e+00  1.531e-02  219.90   <2e-16 ***
proportion_vaccinated      -1.992e-01  4.498e-03  -44.29   <2e-16 ***
edu_primary_lower          -1.465e+00  3.226e-02  -45.40   <2e-16 ***
avg_household_income_capita -2.831e-04  6.329e-06  -44.73   <2e-16 ***
unemployed                  8.289e-01  3.390e-02   24.45   <2e-16 ***
---
Signif. codes:  0 '***' 0.001 '**' 0.01 '*' 0.05 '.' 0.1 ' ' 1
```


For peer review only - http://bmjopen.bmj.com/site/about/guidelines.xhtml

```
(Dispersion parameter for binomial family taken to be 1)

    Null deviance: 2561369  on 2263367  degrees of freedom
Residual deviance: 2338211  on 2263356  degrees of freedom
  (481 observations deleted due to missingness)
AIC: 2338235

Number of Fisher Scoring iterations: 6
```

Since we want to be able to report confidence intervals on these parameters we should also print these values.

```
confint.default(logit_fit)
```

```
                                  2.5 %        97.5 %
(Intercept)                 -3.0501491223 -2.9711607418
age_groupage20to29           1.0342129643  1.1072583584
age_groupage30to39           1.4007062008  1.4641515127
age_groupage40to49           1.8152694329  1.8761546475
age_groupage50to59           2.2454608015  2.3052587953
age_groupage60to69           2.7331468864  2.7926970935
age_groupage70to79           3.0569069010  3.1166836845
age_groupage80_plus          3.3371796759  3.3972028349
proportion_vaccinated       -0.2079929412 -0.1903628320
edu_primary_lower           -1.5280528065 -1.4015799558
avg_household_income_capita -0.0002955328 -0.0002707235
unemployed                   0.7624772639  0.8953497544
```

Since some of the socio-economic variables in particular are candidates for having a concerning level of colinearity, we should print the variance inflation factors (as computed by the `car` package). These values all come out small enough that we don't need to worry further.

```
vif(logit_fit)
```

```
                                GVIF Df GVIF^(1/(2*Df))
age_group                   1.074374  7        1.005137
proportion_vaccinated       1.073312  1        1.036008
edu_primary_lower           2.096518  1        1.447936
avg_household_income_capita 2.507043  1        1.583365
unemployed                  1.810226  1        1.345447
```


For peer review only

LaTeX table summarising the model fit

The following function is useful for generating a LaTeX table summarising the fitted model to avoid transcribing the estimates by hand.

The following helper function is used to make sure that when we are generating the table a sensible format is used for the display of numbers.

```
fmt_est_and_ci <- function(est, ci_min, ci_max) {
  sci_mask <-
    abs(pmin(est, ci_min, ci_max)) < 0.001 | abs(pmax(est, ci_min, ci_max)) > 1000
  ifelse(sci_mask,
         sprintf("%.3e (%.3e, %.3e)", est, ci_min, ci_max),
         sprintf("%.3f (%.3f, %.3f)", est, ci_min, ci_max))
}
```

We can now use that function in the definition of the function that makes the `xtable` object.

```
glm_fit_as_xtable <- function(fit, ...) {

  ns <- c(var = "Variable",
          est_and_ci = "Estimate and CI (95%)",
          sig = "Significance",
          p_val = "Pr(>|z|)")

  fs_df <- as.data.frame(summary(fit)$coefficient)[, c(1,4)]

  tmp <- confint.default(fit)
  stopifnot(ns['p_val'] %in% names(fs_df))
  fs_df[[ns['var']]] <- rownames(fs_df)
  fs_df[[ns['est_and_ci']]] <-
    fmt_est_and_ci(fs_df$Estimate, tmp[,1], tmp[,2])
  fs_df[[ns['sig']]] <-
    ifelse(fs_df[[ns['p_val']]] < 0.05 , "*", "")

  rownames(fs_df) <- NULL

  # Return xtable object
  return(xtable(fs_df[ns[c(1,2,3)]],
                align = c("l", "l", "r", "c"),
                ...))
}
```


For peer review only - http://bmjopen.bmj.com/site/about/guidelines.xhtml

Then we can use this to write the table to disk.

```
print.xtable(glm_fit_as_xtable(logit_fit),
             include.rownames = FALSE,
             type = "latex",
             file = output_files$final_model_table)
```

Session information

To compile this document, the following command could be used:

```
quarto render analysis-mortality.qmd
```

We should record the session information for posterity.

```
sessionInfo()
```

```
R version 4.3.0 (2023-04-21)
Platform: x86_64-pc-linux-gnu (64-bit)
Running under: Ubuntu 22.04.3 LTS

Matrix products: default
BLAS:   /usr/local/lib/R/lib/libRblas.so
LAPACK: /usr/lib/x86_64-linux-gnu/lapack/liblapack.so.3.10.0

locale:
 [1] LC_CTYPE=en_AU.UTF-8       LC_NUMERIC=C
 [3] LC_TIME=en_AU.UTF-8        LC_COLLATE=en_AU.UTF-8
 [5] LC_MONETARY=en_AU.UTF-8    LC_MESSAGES=en_AU.UTF-8
 [7] LC_PAPER=en_AU.UTF-8       LC_NAME=C
 [9] LC_ADDRESS=C               LC_TELEPHONE=C
[11] LC_MEASUREMENT=en_AU.UTF-8 LC_IDENTIFICATION=C

time zone: Australia/Melbourne
tzcode source: system (glibc)

attached base packages:
[1] stats     graphics  grDevices utils     datasets  methods   base

other attached packages:
```


For peer review only - http://bmjopen.bmj.com/site/about/guidelines.xhtml

```
[1] xtable_1.8-4    car_3.1-2       carData_3.0-5   lubridate_1.9.2
[5] readr_2.1.4     dplyr_1.1.2

loaded via a namespace (and not attached):
 [1] crayon_1.5.2      vctrs_0.6.3       cli_3.6.1         knitr_1.43
 [5] rlang_1.1.1       xfun_0.39         generics_0.1.3    jsonlite_1.8.7
 [9] bit_4.0.5         glue_1.6.2        htmltools_0.5.5   hms_1.1.3
[13] fansi_1.0.4       rmarkdown_2.23    abind_1.4-5       evaluate_0.21
[17] tibble_3.2.1      tzdb_0.4.0        fastmap_1.1.1     yaml_2.3.7
[21] lifecycle_1.0.3   compiler_4.3.0    timechange_0.2.0  pkgconfig_2.0.3
[25] rstudioapi_0.15.0 digest_0.6.33     R6_2.5.1          tidyselect_1.2.0
[29] utf8_1.2.3        parallel_4.3.0    vroom_1.6.3       pillar_1.9.0
[33] magrittr_2.0.3    withr_2.5.0       bit64_4.0.5       tools_4.3.0
```

For peer review only

Hospitalisation

Negative binomial GLM of hospitalisation

2023-10-27

Table of contents

Packages . . . 1
Data files . . . 2
Output files . . . 2
Input files . . . 2
Check data files exist . . . 2
(Previous) mortality analysis data . . . 3
(Previous) population data . . . 4
Aggregated socio-economic data . . . 5
Combined dataframe . . . 8
GLM . . . 10
(Quasi-)Poisson regression . . . 11
Negative binomial . . . 13
LaTeX table summarising the model fit . . . 15
Session information . . . 16

Packages

```
library(MASS)
library(dplyr)
library(readr)
library(lubridate)
library(car)
library(xtable)
```


For peer review only

- The `readr` package provides a function, `read_delim` that is faster than the built-in `read.csv` function and provides a progress bar.
- The `car` package provides the `vif` function which we use to check for a concerning about of covariation between the covariates of our model.
- The `MASS` package is needed for the negative binomial GLM model.
- The `xtable` package is needed to make a LaTeX table summarising the final model fit.

Data files

Output files

```
output_files <- list(
  final_dataset = "out/final-hospitalisation-dataset.csv",
  final_nb_model_table = "out/final-hospitalisation-model.tex"
)
```

Input files

Data files live in `data/`. Note that we will use the data set prepared for the hospitalisation analysis since this is still at the individual level and this saves us a non-trivial amount of preprocessing.

Note the `pop_estimates_2020_muni.csv` came from the covariates ZIP XSLX and contains municipality level population estimates.

```
input_data <- list(
  socio_econ_2010 = "data/census2010_muni_covariates.csv",
  mortality_dataset = "out/final-mortality-dataset.csv",
  population_data = "out/cleaned-population.rds"
  )
```

Check data files exist

```
assert_file_exists <- function(fp) {
  if (!file.exists(fp)) {
    stop(sprintf("Cannot find file: %s", fp))
  }
  return(0)
```


For peer review only - http://bmjopen.bmj.com/site/about/guidelines.xhtml

```
}

lapply(X = input_data, FUN = assert_file_exists)
```

```
$socio_econ_2010
[1] 0

$mortality_dataset
[1] 0

$population_data
[1] 0
```

If you want to check that the files you have match the ones used, here are some checksums (which can be obtained with `sha256sum *` from within `data/`.

```
9116d21e84b8ab4d4e8b2437644248618dc39affc7f6101d8ac7c631950c2e8e  agesex_coverage_muni_new.c
7a5a6d07ab0beadd1c015d087802192aa02a227fad72ab33d8e0299600db29b0  census2010_muni_covariates
e7db11cdf762b65b51df22ab4a71bf6f57f20d503ec568f34788c62bd5e0073f  dates_50.rds
2ee48a45bb88d3b78fa9c5571918d5bce23aa135fc3234ff1e1cc3836cfb4f32  sivep_20092021.csv
4c7bfe45057871a354ef00ab7a905b1daafff844dbd0d89045f8ed8c5f06c67a  sivep_2020.csv
```

For the population sizes in each of the municipalities there is `covariates.zip` in `data/`

```
e06625739f8c0a3e41c41a23a82a4cb3c6ca51782872a12d839d12483ecd7229  covariates.zip
```

which contains an XLSX which appears to have population sizes mapped to municipality codes. We save to CSV in `out/` which has the hash

```
bb947d91b30496b6252d4bee7ef6ff98eeef48bbc3bbd445068455a56b82275e  pop_estimates_2020_muni.cs
```

(Previous) mortality analysis data

Because we don't want to do all the preprocessing from the mortality analysis again, we can just read in the data set that we prepared in that analysis. We do remove the socio economic variables so that they cannot accidentally be used instead of the other versions of these which are aggregated up to the state level below.

For peer review only

For peer review only - http://bmjopen.bmj.com/site/about/guidelines.xhtml

```r
mortality_df <- input_data$mortality_dataset |>
  read_csv(show_col_types = FALSE) |>
  mutate(patient_address_muni_code = as.character(patient_address_muni_code)) |>
  select(-died_boolean,
         -patient_sex,
         -proportion_vaccinated,
         -vac_campaign,
         -unemployed,
         -informal,
         -edu_primary_lower,
         -avg_household_income_capita)
```

(Previous) population data

We redefine this function because we need it to get the population data into a more convenient format.

```r
age_group_int_to_string <- function(age_groups) {
  case_when(age_groups == 19 ~ "ageunder20",
            age_groups == 20 ~ "age20to29",
            age_groups == 30 ~ "age30to39",
            age_groups == 40 ~ "age40to49",
            age_groups == 50 ~ "age50to59",
            age_groups == 60 ~ "age60to69",
            age_groups == 70 ~ "age70to79",
            age_groups == 80 ~ "age80_plus")
}
```

The state code is the first two digits of the six digits used to identify the municipality. Rather than process the population data again, we just read this in from the RDS saved in the previous processing.

```r
pop_df <-
  input_data$population_data |>
  readRDS() |>
  mutate(muni_code = as.character(muni_code),
         state_code = gsub("[0-9]{4}$", "", muni_code),
         age_group = age_group_int_to_string(grouped_age)) |>
  select(muni_code,
         age_group,
```


For peer review only - http://bmjopen.bmj.com/site/about/guidelines.xhtml

```
        population,
        state_code)
```

N.b. Note that this dataframe keeps the population values age structured.

Aggregated socio-economic data

We start be reading in and setting up the socio economic data at the municipality level, including the state population size. Including the population size here makes it easier to do a state level aggregation later.

```
tmp <- input_data$socio_econ_2010 |>
  read_csv(show_col_types = FALSE) |>
  mutate(muni_code = gsub("[0-9]{1}$", "", as.character(code_muni))) |>
  select(-code_muni)

tmp_muni_lvl_socio_econ_df <- left_join(pop_df, tmp, by = "muni_code")
rm(pop_df)
rm(tmp)
gc()
```

```
           used (Mb) gc trigger  (Mb) max used  (Mb)
Ncells   892917 47.7    1710237  91.4  1349802  72.1
Vcells 10990195 83.9   36463450 278.2 31318311 239.0
```

Some of the municipalities are missing socio-economic data, but this only happens for a small number (four) of states, so in these instances we will replace the missing values by the weighted state average.

The following painful loop goes across the states with a municipality with missing data and fills in the missing values with the weighted average of the value of that variable across the other municipalities for which the data is available. It also prints out a description of how many records it is modifying which comes in at a very small proportion.

```
for (state in unique(tmp_muni_lvl_socio_econ_df$state_code)) {
  state_mask <- tmp_muni_lvl_socio_econ_df$state_code == state
  state_df <- tmp_muni_lvl_socio_econ_df[state_mask, ]
  if (any(is.na(state_df))) {
    print(sprintf("working on state %s", state))
    na_unemployed_mask <- is.na(tmp_muni_lvl_socio_econ_df$unemployed)
```


For peer review only - http://bmjopen.bmj.com/site/about/guidelines.xhtml

```
    na_informal_mask <- is.na(tmp_muni_lvl_socio_econ_df$informal)
    na_edu_primary_lower_mask <- is.na(tmp_muni_lvl_socio_econ_df$edu_primary_lower)
    na_income_mask <- is.na(tmp_muni_lvl_socio_econ_df$avg_household_income_capita)

    tmp <- state_df |>
      group_by(muni_code) |>
      summarise(muni_pop = sum(population),
                unq_unemployed = unique(unemployed),
                unq_informal = unique(informal),
                unq_edu_primary_lower = unique(edu_primary_lower),
                unq_income = unique(avg_household_income_capita))

    wm_unemployed <- weighted.mean(x = tmp$unq_unemployed,
                                   w = tmp$muni_pop,
                                   na.rm = TRUE)
    wm_informal <- weighted.mean(x = tmp$unq_informal,
                                 w = tmp$muni_pop,
                                 na.rm = TRUE)
    wm_edu_primary_lower <- weighted.mean(x = tmp$unq_edu_primary_lower,
                                          w = tmp$muni_pop,
                                          na.rm = TRUE)
    wm_income <- weighted.mean(x = tmp$unq_income,
                               w = tmp$muni_pop,
                               na.rm = TRUE)

    tmp_muni_lvl_socio_econ_df[state_mask & na_unemployed_mask,]$unemployed <- wm_unemploy
    print(sprintf("  filling in %d missing values with %f for unemployed",
              sum(state_mask & na_unemployed_mask), wm_unemployed))
    tmp_muni_lvl_socio_econ_df[state_mask & na_informal_mask,]$informal <- wm_informal
    print(sprintf("  filling in %d missing values with %f for informal",
              sum(state_mask & na_informal_mask), wm_informal))
    tmp_muni_lvl_socio_econ_df[state_mask & na_edu_primary_lower_mask,]$edu_primary_lower
    print(sprintf("  filling in %d missing values with %f for edu_primary_lower",
              sum(state_mask & na_edu_primary_lower_mask), wm_edu_primary_lower))
    tmp_muni_lvl_socio_econ_df[state_mask & na_income_mask,]$avg_household_income_capita <
    print(sprintf("  filling in %d missing values with %f for for avg_household_income_cap
              sum(state_mask & na_income_mask), wm_income))
    }
}
```

```
[1] "working on state 15"
```


For peer review only - http://bmjopen.bmj.com/site/about/guidelines.xhtml

```
[1] "  filling in 8 missing values with 0.235284 for unemployed"
[1] "  filling in 8 missing values with 0.092493 for informal"
[1] "  filling in 8 missing values with 0.307297 for edu_primary_lower"
[1] "  filling in 8 missing values with 424.206534 for for avg_household_income_capita"
[1] "working on state 42"
[1] "  filling in 16 missing values with 0.112102 for unemployed"
[1] "  filling in 16 missing values with 0.038951 for informal"
[1] "  filling in 16 missing values with 0.289139 for edu_primary_lower"
[1] "  filling in 16 missing values with 979.963089 for for avg_household_income_capita"
[1] "working on state 43"
[1] "  filling in 8 missing values with 0.137998 for unemployed"
[1] "  filling in 8 missing values with 0.050790 for informal"
[1] "  filling in 8 missing values with 0.280631 for edu_primary_lower"
[1] "  filling in 8 missing values with 947.221227 for for avg_household_income_capita"
[1] "working on state 50"
[1] "  filling in 8 missing values with 0.199913 for unemployed"
[1] "  filling in 8 missing values with 0.062054 for informal"
[1] "  filling in 8 missing values with 0.263784 for edu_primary_lower"
[1] "  filling in 8 missing values with 788.500330 for for avg_household_income_capita"
```

```
stopifnot(!any(is.na(tmp_muni_lvl_socio_econ_df)))
```

These values are very similar to the country wide unweighted averages shown by the following:

```
tmp_muni_lvl_socio_econ_df |>
  select(unemployed,
         informal,
         edu_primary_lower,
         avg_household_income_capita ) |>
  colMeans()
```

```
                 unemployed                    informal
                 0.24181327                  0.06512755
          edu_primary_lower avg_household_income_capita
                 0.36311907                484.56685182
```

Since we do not have the socio economic data at an age group level, we need to aggregate this up to be able to work at the state level. We will do this respecting the population sizes in each municipality.

For peer review only - http://bmjopen.bmj.com/site/about/guidelines.xhtml

```
tmp_state_age_socio_econ_df <-
  tmp_muni_lvl_socio_econ_df |>
  group_by(muni_code, age_group) |>
  summarise(muni_pop = sum(population),
            state_code = unique(state_code),
            unemployed = unique(unemployed),
            informal = unique(informal),
            edu_primary_lower = unique(edu_primary_lower),
            avg_household_income_capita = unique(avg_household_income_capita),
            .groups = "drop") |>
  group_by(state_code, age_group) |>
  summarise(state_pop = sum(muni_pop),
            s_unemployed = weighted.mean(x = unemployed,
                                         w = muni_pop),
            s_informal = weighted.mean(x = informal,
                                       w = muni_pop),
            s_edu_primary_lower = weighted.mean(x = edu_primary_lower,
                                                w = muni_pop),
            s_avg_household_income_capita = weighted.mean(x = avg_household_income_capita,
                                                          w = muni_pop),
            .groups = "keep")
```

Combined dataframe

When combining up to state level we compute the average vaccination start date across each age group within each state. This will introduce some bias but it captures the intended effect. We want to have a dataframe with one record for each hospitalisation but it also needs to have the age group and a shared vaccination start date for this cohort so we have a sensible shared pre/post vaccination divide.

```
disaggregated_data_df <-
  left_join(
    mortality_df,
    rename(tmp_muni_lvl_socio_econ_df,
           patient_address_muni_code = muni_code),
    by = c("patient_address_muni_code", "age_group")
  ) |>
  group_by(age_group, state_code) |>
  summarise(date_symptoms = date_symptoms,
            patient_address_muni_code = patient_address_muni_code,
```


For peer review only - http://bmjopen.bmj.com/site/about/guidelines.xhtml

```r
             vac_start_date = rep(mean(vac_start_date), length(date_symptoms))) |>
  mutate(vac_campaign =  ifelse(date_symptoms < vac_start_date,
                               "pre", "post"))
```

```
Warning: Returning more (or less) than 1 row per `summarise()` group was deprecated in
dplyr 1.1.0.
i Please use `reframe()` instead.
i When switching from `summarise()` to `reframe()`, remember that `reframe()`
  always returns an ungrouped data frame and adjust accordingly.
```

```
`summarise()` has grouped output by 'age_group', 'state_code'. You can override
using the `.groups` argument.
```

N.b. The populations in this dataframe are the municipality populations. And there are some municipalities that are appear in the socio economic data but not in the case data so these get removed in the left join above. So we do not rely on this join to produce a sensible population size.

N.b. Don't worry about the warning here, provided you are using a version of `dplyr` that has not removed the deprecated function this should work fine.

Now we need to aggregate these hospitalisations.

```r
aggregated_data_df <-
  disaggregated_data_df |>
  group_by(age_group,
           ## patient_address_muni_code,
           state_code,
           vac_campaign) |>
  summarise(num_hosp = length(patient_address_muni_code),
            vac_start_date = unique(vac_start_date),
            .groups = "drop") |>
  left_join(tmp_state_age_socio_econ_df, by = c("state_code", "age_group")) |>
  mutate(age_group = relevel(as.factor(age_group), ref = "ageunder20"))
```

N.b. there are no "post" vaccination records for the under 20s because vaccination hadn't reached the halfway mark by the end of the study period. As a result, quick attempts to count the population size by grouping by state codes will give a under estimate, you need to select just the records from the pre-vaccination time epoch.

For peer review only - http://bmjopen.bmj.com/site/about/guidelines.xhtml

Finally, we need to include the duration across which we are aggregating so that we can offset by both the population and number of people so that the regression gives us the rate of hospitalisations per person per day.

```
pre_vac_mask <- aggregated_data_df$vac_campaign == "pre"
final_data_df <- aggregated_data_df
final_data_df$obs_duration <- NA
tmp <- ymd(final_data_df$vac_start_date)
final_data_df[pre_vac_mask,]$obs_duration <-
  as.numeric(tmp[pre_vac_mask] - ymd("2020-03-01"))
final_data_df[!pre_vac_mask,]$obs_duration <-
  as.numeric(ymd("2021-09-06") - tmp[!pre_vac_mask])
```

Because it makes more sense to talk about the change in rate in response to substantial vaccination levels, we make the "pre" interval as the reference.

```
final_data_df$vac_campaign <- relevel(as.factor(final_data_df$vac_campaign), ref = "pre")
```

We will save a copy of this data frame, not to use in the analysis, but so there is a record of it in case we need to check it later.

```
write.table(x = final_data_df,
            file = output_files$final_dataset,
            sep = ",",
            row.names = FALSE)
```

GLM

The GLM uses a log-link function with the (log) population size and the (log) observation duration as offsets. This means that the expected number of hospitalisations, μ is modelled by

$$\mu = N_{\text{pop}} T_{\text{days}} \exp\{\mathbf{X}\beta\}$$

where N_{pop} is the relevant population size, T_{days} is the number of days during which the hospitalisations were observed and $\mathbf{X}\beta$ is a linear combination of the remaining covariates.

The advantage of including these variables as offsets in this way is that we get a *daily per capita* rate out of the model.

For peer review only - http://bmjopen.bmj.com/site/about/guidelines.xhtml

(Quasi-)Poisson regression

Because unemployment has substantial correlations with the average household income variable, we remove this to avoid issues with colinearity. This was discovered via the presence of a large VIF.

```
q_poisson_fit <- glm(num_hosp ~ age_group +
                       vac_campaign +
                       s_avg_household_income_capita +
                       s_edu_primary_lower +
                       ## s_unemployed +
                       offset(log(obs_duration)) +
                       offset(log(state_pop)),
                     data = final_data_df,
                     family = quasipoisson())
```

```
summary(q_poisson_fit)
```

```
Call:
glm(formula = num_hosp ~ age_group + vac_campaign + s_avg_household_income_capita +
    s_edu_primary_lower + offset(log(obs_duration)) + offset(log(state_pop)),
    family = quasipoisson(), data = final_data_df)

Coefficients:
                               Estimate Std. Error t value Pr(>|t|)
(Intercept)                  -1.251e+01  2.225e-01 -56.199  < 2e-16 ***
age_groupage20to29            1.285e-01  7.827e-02   1.642  0.10139
age_groupage30to39            9.107e-01  6.406e-02  14.218  < 2e-16 ***
age_groupage40to49            1.424e+00  6.013e-02  23.685  < 2e-16 ***
age_groupage50to59            1.849e+00  5.828e-02  31.722  < 2e-16 ***
age_groupage60to69            2.201e+00  5.877e-02  37.453  < 2e-16 ***
age_groupage70to79            2.657e+00  6.057e-02  43.867  < 2e-16 ***
age_groupage80_plus           3.196e+00  6.260e-02  51.061  < 2e-16 ***
vac_campaignpost             -9.467e-02  3.530e-02  -2.682  0.00763 **
s_avg_household_income_capita  5.901e-04  8.338e-05   7.077 6.82e-12 ***
s_edu_primary_lower          -7.536e-01  5.965e-01  -1.263  0.20722
---
Signif. codes:  0 '***' 0.001 '**' 0.01 '*' 0.05 '.' 0.1 ' ' 1

(Dispersion parameter for quasipoisson family taken to be 381.1353)
```


For peer review only - http://bmjopen.bmj.com/site/about/guidelines.xhtml

```
    Null deviance: 2304187  on 403  degrees of freedom
Residual deviance:  151951  on 393  degrees of freedom
AIC: NA

Number of Fisher Scoring iterations: 4
```

```
confint.default(q_poisson_fit)
```

```
                                   2.5 %        97.5 %
(Intercept)                  -12.942330792 -1.207001e+01
age_groupage20to29            -0.024887847  2.819238e-01
age_groupage30to39             0.785202963  1.036297e+00
age_groupage40to49             1.306210093  1.541899e+00
age_groupage50to59             1.734511728  1.962961e+00
age_groupage60to69             2.085776824  2.316136e+00
age_groupage70to79             2.538251013  2.775678e+00
age_groupage80_plus            3.073515518  3.318888e+00
vac_campaignpost              -0.163856323 -2.548826e-02
s_avg_household_income_capita  0.000426641  7.534905e-04
s_edu_primary_lower           -1.922664681  4.155395e-01
```

Since some of the socio-economic variables in particular are candidates for having a concerning level of colinearity, we should print the variance inflation factors (as computed by the car package). These values all come out small enough that we don't need to worry further.

```
vif(q_poisson_fit)
```

```
                                  GVIF Df GVIF^(1/(2*Df))
age_group                     1.109441  7        1.007446
vac_campaign                  1.076342  1        1.037469
s_avg_household_income_capita 2.847989  1        1.687599
s_edu_primary_lower           2.842682  1        1.686026
```

However, we don't want to use the Poisson model so we will also fit a negative binomial model.

For peer review only - http://bmjopen.bmj.com/site/about/guidelines.xhtml

Negative binomial

To address the substantial amount of overdispersion in the dataset, while sticking to a likelihood framework, we will use the negative binomial distribution to model.

Recall that negative binomial GLM regression is provided by the MASS package.

```
nb_fit <- glm.nb(num_hosp ~ age_group +
                   vac_campaign +
                   s_avg_household_income_capita +
                   s_edu_primary_lower +
                   ## s_unemployed +
                   offset(log(obs_duration)) +
                   offset(log(state_pop)),
                 data = final_data_df)
```

```
summary(nb_fit)
```

```
Call:
glm.nb(formula = num_hosp ~ age_group + vac_campaign + s_avg_household_income_capita +
    s_edu_primary_lower + offset(log(obs_duration)) + offset(log(state_pop)),
    data = final_data_df, init.theta = 5.432893292, link = log)

Coefficients:
                               Estimate Std. Error z value Pr(>|z|)
(Intercept)                  -1.311e+01  2.553e-01 -51.361  < 2e-16 ***
age_groupage20to29            9.368e-02  1.048e-01   0.894    0.371
age_groupage30to39            8.491e-01  1.040e-01   8.163 3.26e-16 ***
age_groupage40to49            1.400e+00  1.038e-01  13.481  < 2e-16 ***
age_groupage50to59            1.917e+00  1.038e-01  18.469  < 2e-16 ***
age_groupage60to69            2.493e+00  1.037e-01  24.032  < 2e-16 ***
age_groupage70to79            3.008e+00  1.037e-01  29.005  < 2e-16 ***
age_groupage80_plus           3.558e+00  1.038e-01  34.291  < 2e-16 ***
vac_campaignpost             -3.732e-01  4.460e-02  -8.368  < 2e-16 ***
s_avg_household_income_capita  8.038e-04  1.032e-04   7.786 6.92e-15 ***
s_edu_primary_lower           5.821e-01  6.858e-01   0.849    0.396
---
Signif. codes:  0 '***' 0.001 '**' 0.01 '*' 0.05 '.' 0.1 ' ' 1

(Dispersion parameter for Negative Binomial(5.4329) family taken to be 1)
```


For peer review only - http://bmjopen.bmj.com/site/about/guidelines.xhtml

```
    Null deviance: 3054.42  on 403  degrees of freedom
Residual deviance:  427.16  on 393  degrees of freedom
AIC: 6446.3

Number of Fisher Scoring iterations: 1

              Theta:  5.433
          Std. Err.:  0.387

 2 x log-likelihood:  -6422.323
```

```
confint.default(nb_fit)
```

```
                                   2.5 %        97.5 %
(Intercept)                 -1.361435e+01 -12.613486653
age_groupage20to29          -1.116569e-01   0.299016717
age_groupage30to39           6.452518e-01   1.053000486
age_groupage40to49           1.196463e+00   1.603540085
age_groupage50to59           1.713302e+00   2.120111970
age_groupage60to69           2.289520e+00   2.696123827
age_groupage70to79           2.805180e+00   3.211764274
age_groupage80_plus          3.354731e+00   3.761466202
vac_campaignpost            -4.606533e-01  -0.285816886
s_avg_household_income_capita  6.014233e-04   0.001006087
s_edu_primary_lower         -7.620310e-01   1.926271435
```

Since some of the socio-economic variables in particular are candidates for having a concerning level of colinearity, we should print the variance inflation factors (as computed by the `car` package). These values all come out small enough that we don't need to worry further.

```
vif(nb_fit)
```

```
                                  GVIF Df GVIF^(1/(2*Df))
age_group                     1.077055  7        1.005316
vac_campaign                  1.066380  1        1.032657
s_avg_household_income_capita 1.772904  1        1.331504
s_edu_primary_lower           1.785122  1        1.336084
```


For peer review only - http://bmjopen.bmj.com/site/about/guidelines.xhtml

LaTeX table summarising the model fit

The following function is useful for generating a LaTeX table summarising the fitted model to avoid transcribing the estimates by hand.

The following helper function is used to make sure that when we are generating the table a sensible format is used for the display of numbers.

```
fmt_est_and_ci <- function(est, ci_min, ci_max) {
  sci_mask <-
    abs(pmin(est, ci_min, ci_max)) < 0.001 | abs(pmax(est, ci_min, ci_max)) > 1000
  ifelse(sci_mask,
         sprintf("%.3e (%.3e, %.3e)", est, ci_min, ci_max),
         sprintf("%.3f (%.3f, %.3f)", est, ci_min, ci_max))
}
```

We can now use that function in the definition of the function that makes the `xtable` object.

```
glm_fit_as_xtable <- function(fit, ...) {

  ns <- c(var = "Variable",
          est_and_ci = "Estimate and CI (95%)",
          sig = "Significance",
          p_val = "Pr(>|z|)")

  fs_df <- as.data.frame(summary(fit)$coefficient)[, c(1,4)]

  tmp <- confint.default(fit)
  stopifnot(ns['p_val'] %in% names(fs_df))
  fs_df[[ns['var']]] <- rownames(fs_df)
  fs_df[[ns['est_and_ci']]] <-
    fmt_est_and_ci(fs_df$Estimate, tmp[,1], tmp[,2])
  fs_df[[ns['sig']]] <-
    ifelse(fs_df[[ns['p_val']]] < 0.05 , "*", "")

  rownames(fs_df) <- NULL

  # Return xtable object
  return(xtable(fs_df[ns[c(1,2,3)]],
                align = c("l", "l", "r", "c"),
                ...))
}
```


For peer review only - http://bmjopen.bmj.com/site/about/guidelines.xhtml

Then we can use this to write the table to disk.

```
print.xtable(glm_fit_as_xtable(nb_fit),
             include.rownames = FALSE,
             type = "latex",
             file = output_files$final_nb_model_table)
```

Session information

To compile this document, the following command could be used:

```
quarto render analysis-hospitalisation.qmd
```

We should record the session information for posterity.

```
sessionInfo()
```

```
R version 4.3.0 (2023-04-21)
Platform: x86_64-pc-linux-gnu (64-bit)
Running under: Ubuntu 22.04.3 LTS

Matrix products: default
BLAS:   /usr/local/lib/R/lib/libRblas.so
LAPACK: /usr/lib/x86_64-linux-gnu/lapack/liblapack.so.3.10.0

locale:
 [1] LC_CTYPE=en_AU.UTF-8       LC_NUMERIC=C
 [3] LC_TIME=en_AU.UTF-8        LC_COLLATE=en_AU.UTF-8
 [5] LC_MONETARY=en_AU.UTF-8    LC_MESSAGES=en_AU.UTF-8
 [7] LC_PAPER=en_AU.UTF-8       LC_NAME=C
 [9] LC_ADDRESS=C               LC_TELEPHONE=C
[11] LC_MEASUREMENT=en_AU.UTF-8 LC_IDENTIFICATION=C

time zone: Australia/Melbourne
tzcode source: system (glibc)

attached base packages:
[1] stats     graphics  grDevices utils     datasets  methods   base

other attached packages:
```


For peer review only - http://bmjopen.bmj.com/site/about/guidelines.xhtml

```
[1] xtable_1.8-4    car_3.1-2       carData_3.0-5   lubridate_1.9.2
[5] readr_2.1.4     dplyr_1.1.2     MASS_7.3-58.4

loaded via a namespace (and not attached):
 [1] bit_4.0.5         jsonlite_1.8.7    compiler_4.3.0    crayon_1.5.2
 [5] tidyselect_1.2.0  parallel_4.3.0    yaml_2.3.7        fastmap_1.1.1
 [9] R6_2.5.1          generics_0.1.3    knitr_1.43        tibble_3.2.1
[13] pillar_1.9.0      tzdb_0.4.0        rlang_1.1.1       utf8_1.2.3
[17] xfun_0.39         bit64_4.0.5       timechange_0.2.0  cli_3.6.1
[21] withr_2.5.0       magrittr_2.0.3    digest_0.6.33     vroom_1.6.3
[25] rstudioapi_0.15.0 hms_1.1.3         lifecycle_1.0.3   vctrs_0.6.3
[29] evaluate_0.21     glue_1.6.2        abind_1.4-5       fansi_1.0.4
[33] rmarkdown_2.23    tools_4.3.0       pkgconfig_2.0.3   htmltools_0.5.5
```

For peer review only

For peer review only - http://bmjopen.bmj.com/site/about/guidelines.xhtml

Supplementary Information: The Brazilian COVID-19 vaccination campaign: A modelling analysis of socio-demographic factors on uptake

Sabrina L. Li[1,2,*,=], Carlos A. Prete Jr[3,=], Alexander E. Zarebski[4,5,=], Andreza A. de Souza Santos[6], Ester C. Sabino[7], Vitor H. Nascimento[3], Chieh-Hsi Wu[8], and Jane P. Messina[1,9]

[1]School of Geography and the Environment, University of Oxford, Oxford, UK
[2]School of Geography, University of Nottingham, Nottingham, UK
[3]Department of Electronic Systems Engineering, University of São Paulo, São Paulo, Brazil
[4]Department of Biology, University of Oxford, Oxford, UK
[5]School of Mathematics and Statistics, University of Melbourne, Melbourne, Australia
[6]Oxford School of Global and Area Studies, Latin American Centre, University of Oxford, Oxford, UK
[7]Departamento de Molestias Infecciosas e Parasitarias & Instituto de Medicina Tropical da Faculdade de Medicina da Universidade de São Paulo, São Paulo, Brazil
[8]Mathematical Sciences, University of Southampton, Southampton, UK
[9]Oxford School of Global and Area Studies, University of Oxford, Oxford, UK
[=]These authors contributed equally
[*]Corresponding author: Sabrina.Li@nottingham.ac.uk

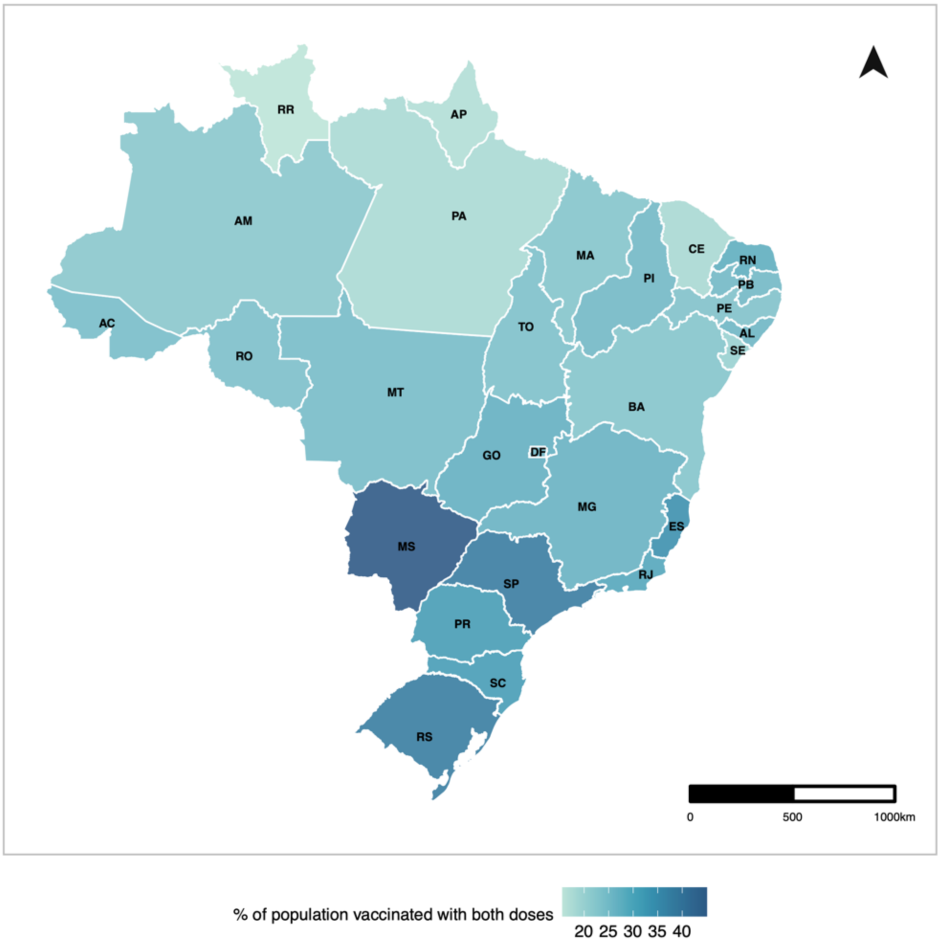

Figure S1: Proportion of population fully vaccinated.

For peer review only - http://bmjopen.bmj.com/site/about/guidelines.xhtml

Risk of COVID-19 death by age-group

The equation describing the log-odds of death among SARI patients is expressed as

$$\begin{aligned}\text{logit}(p_i) = \beta_0 &+ \beta_1 \cdot (\text{Age group of } i) \\ &+ \beta_2 \cdot (\text{Vaccination coverage in age group of } i \text{ in their municipality}) \\ &+ \beta_3 \cdot (\text{Low education attainment in municipality of } i) \\ &+ \beta_4 \cdot (\text{Average income per capita in municipality of } i) \\ &+ \beta_5 \cdot (\text{Unemployment in municipality of } i) + \epsilon_i,\end{aligned} \tag{1}$$

where $p_i = \mathbb{P}(Y_i = 1)$ is the log-odds of death where Y_i is an indicator variable for whether the ith SARI patient's outcome: dead = 1, and alive = 0. The β_j are the coefficients of the model and ϵ_i is the error term. For reference, the logit function is defined as $\text{logit}(p) = \log(p/(1-p))$.

As a form of sensitivity analysis, we fit the model at the individual level by including only patients that had a recorded vaccination status (e.g. vaccinated with at least one dose, or no vaccination). Here we included vaccination status as a binary indicator in the model to replace the age-group specific vaccination coverage. While this avoids the potential for an ecological fallacy (i.e. assuming population level vaccination levels are indicative of the individuals represented in the data), we cannot rule out the possibility that data may not be missing at random, which may introduce biases in the estimates.

The resulting parameter estimates from this model and their confidence intervals are given in Table S1.

Variable	Estimate and CI (95%)	Significance
Intercept	-3.011 (-3.050, -2.971)	*
Age group#: 20–29	1.071 (1.034, 1.107)	*
Age group: 30–39	1.432 (1.401, 1.464)	*
Age group: 40–49	1.846 (1.815, 1.876)	*
Age group: 50–59	2.275 (2.245, 2.305)	*
Age group: 60–69	2.763 (2.733, 2.793)	*
Age group: 70–79	3.087 (3.057, 3.117)	*
Age group: ≥ 80	3.367 (3.337, 3.397)	*
Proportion vaccinated	-0.199 (-0.208, -0.190)	*
Primary education or lower	-1.465 (-1.528, -1.402)	*
Income per capita (US$ PPP)	-3.930×10^{-4} (-4.101×10^{-4}, -3.757×10^{-4})	*
Unemployed	0.829 (0.762, 0.895)	*

\# Baseline age group for comparison is under 20 years old
* statistically significant p-value < 0.05

Table S1: Logistic regression model analysis — death risk of SARI patient given age group, age group-specific vaccination coverage, and socioeconomic indicators.

For peer review only - http://bmjopen.bmj.com/site/about/guidelines.xhtml

Risk of COVID-19 hospitalisation by age group

The equation describing the expected number of hospitalisations in this model is

$$\begin{aligned}\log(\mathbb{E}[Y_i]) = &\log(N_i) + \log(T_i)\\ &+ \beta_0 + \beta_1 \cdot (\text{Age group for } i)\\ &+ \beta_2 \cdot (\text{Campaign status indicator for age group of } i \text{ in that state})\\ &+ \beta_3 \cdot (\text{Average income per capita in state of } i)\\ &+ \beta_4 \cdot (\text{Low education attainment in state of } i) + \epsilon_i\end{aligned} \tag{2}$$

where Y_i is the number of hospitalisations in a particular age group within a particular state during the period of time before (or after) approximately half of that age group was vaccinated in that state. The distribution of Y_i given the mean is assumed to follow a negative binomial distribution (with estimated dispersion parameter of 5.43).

The variables N_i and T_i represent the number of people in that age group in that state, and the number of days in that period respectively. These offset variables are included so that the result can be interpreted as a daily per capita risk.

The resulting parameter estimates from this model and their confidence intervals are given in Table S2. The state level average unemployment was not included as a covariate because it was strongly correlated with the over socio-economic variables (as measured by VIF). This colinearity was less substantial at the municipality level.

Variable	Estimate and CI (95%)	Significance
Intercept	-13.114 (-13.614, -12.613)	*
Age group#: 20–29	0.094 (-0.112, 0.299)	
Age group: 30–39	0.849 (0.645, 1.053)	*
Age group: 40–49	1.400 (1.196, 1.604)	*
Age group: 50–59	1.917 (1.713, 2.120)	*
Age group: 60–69	2.493 (2.290, 2.696)	*
Age group: 70–79	3.008 (2.805, 3.212)	*
Age group: ≥ 80	3.558 (3.355, 3.761)	*
Vaccination campaign (yes)	-0.373 (-0.461, -0.286)	*
Income per capita (US$ PPP)	11.157×10^{-4} (8.345×10^{-4}, 1.396×10^{-3})	*
Primary education or lower	0.582 (-0.762, 1.926)	

Baseline age group for comparison is under 20 years old

* statistically significant p-value < 0.05

Table S2: Negative binomial regression model analysis — hospitalisation risk given age group, socioeconomic indicators, and indication of age-group specific vaccination campaign

For peer review only

Reporting checklist for cross sectional study.

Based on the STROBE cross sectional guidelines.

Instructions to authors

Complete this checklist by entering the page numbers from your manuscript where readers will find each of the items listed below.

Your article may not currently address all the items on the checklist. Please modify your text to include the missing information. If you are certain that an item does not apply, please write "n/a" and provide a short explanation.

Upload your completed checklist as an extra file when you submit to a journal.

In your methods section, say that you used the STROBE cross sectionalreporting guidelines, and cite them as:

von Elm E, Altman DG, Egger M, Pocock SJ, Gotzsche PC, Vandenbroucke JP. The Strengthening the Reporting of Observational Studies in Epidemiology (STROBE) Statement: guidelines for reporting observational studies.

		Reporting Item	Page Number
**Title and abstract**			
Title	#1a	Indicate the study’s design with a commonly used term in the title or the abstract	1
Abstract	#1b	Provide in the abstract an informative and balanced summary of what was done and what was found	1
**Introduction**			
Background / rationale	#2	Explain the scientific background and rationale for the investigation being reported	2
Objectives	#3	State specific objectives, including any prespecified hypotheses	2
**Methods**			
Study design	#4	Present key elements of study design early in the paper	3-6
Setting	#5	Describe the setting, locations, and relevant dates, including periods of	3-6

For peer review only

		recruitment, exposure, follow-up, and data collection	
Eligibility criteria	#6a	Give the eligibility criteria, and the sources and methods of selection of participants.	3-6
	#7	Clearly define all outcomes, exposures, predictors, potential confounders, and effect modifiers. Give diagnostic criteria, if applicable	3-6
Data sources / measurement	#8	For each variable of interest give sources of data and details of methods of assessment (measurement). Describe comparability of assessment methods if there is more than one group. Give information separately for for exposed and unexposed groups if applicable.	3-6
Bias	#9	Describe any efforts to address potential sources of bias	3-6
Study size	#10	Explain how the study size was arrived at	3-6
Quantitative variables	#11	Explain how quantitative variables were handled in the analyses. If applicable, describe which groupings were chosen, and why	3-6
Statistical methods	#12a	Describe all statistical methods, including those used to control for confounding	3-6
Statistical methods	#12b	Describe any methods used to examine subgroups and interactions	3-6
Statistical methods	#12c	Explain how missing data were addressed	3-6
Statistical methods	#12d	If applicable, describe analytical methods taking account of sampling strategy	3-6
Statistical methods	#12e	Describe any sensitivity analyses	3-6
**Results**			
Participants	#13a	Report numbers of individuals at each stage of study—eg numbers potentially eligible, examined for eligibility, confirmed eligible, included in the study, completing follow-up, and analysed. Give information separately for for exposed and unexposed groups if applicable.	3-6
Participants	#13b	Give reasons for non-participation at each stage	3-6
Participants	#13c	Consider use of a flow diagram	3-6

For peer review only

Descriptive data	#14a	Give characteristics of study participants (eg demographic, clinical, social) and information on exposures and potential confounders. Give information separately for exposed and unexposed groups if applicable.	3-6
Descriptive data	#14b	Indicate number of participants with missing data for each variable of interest	6
Outcome data	#15	Report numbers of outcome events or summary measures. Give information separately for exposed and unexposed groups if applicable.	6-7
Main results	#16a	Give unadjusted estimates and, if applicable, confounder-adjusted estimates and their precision (eg, 95% confidence interval). Make clear which confounders were adjusted for and why they were included	6-13
Main results	#16b	Report category boundaries when continuous variables were categorized	6-13
Main results	#16c	If relevant, consider translating estimates of relative risk into absolute risk for a meaningful time period	6-13
Other analyses	#17	Report other analyses done—e.g., analyses of subgroups and interactions, and sensitivity analyses	6-13
**Discussion**			
Key results	#18	Summarise key results with reference to study objectives	13-14
Limitations	#19	Discuss limitations of the study, taking into account sources of potential bias or imprecision. Discuss both direction and magnitude of any potential bias.	15
Interpretation	#20	Give a cautious overall interpretation considering objectives, limitations, multiplicity of analyses, results from similar studies, and other relevant evidence.	13-16
Generalisability	#21	Discuss the generalisability (external validity) of the study results	16
**Other Information**			
Funding	#22	Give the source of funding and the role of the funders for the present study and, if applicable, for the original study on which the present article is based	16

The STROBE checklist is distributed under the terms of the Creative Commons Attribution License CC-BY.
This checklist was completed on 16. May 2023 using https://www.goodreports.org/, a tool made by the EQUATOR Network in collaboration with Penelope.ai

For peer review only
